# Sacraoxides A–G, Bioactive Cembranoids from Gum Resin of *Boswellia sacra*

**DOI:** 10.3389/fchem.2021.649287

**Published:** 2021-03-30

**Authors:** Bingyang Zhang, Di Liu, Wenyue Ji, Kouharu Otsuki, Koji Higai, Feng Zhao, Wei Li, Kazuo Koike, Feng Qiu

**Affiliations:** ^1^ School of Chinese Materia Medica, Tianjin University of Traditional Chinese Medicine, Tianjin, China; ^2^ Faculty of Pharmaceutical Sciences, Toho University, Tokyo, Japan; ^3^ School of Pharmacy, Key Laboratory of Molecular Pharmacology and Drug Evaluation (Yantai University), Ministry of Education, Collaborative Innovation Center of Advanced Drug Delivery System and Biotech Drugs in Universities of Shandong, Yantai University, Yantai, China

**Keywords:** *Boswellia sacra*, olibanum, phytochemistry, cembranoids, structure elucidation, anti-inflammatory

## Abstract

Seven undescribed cembranoids, sacraoxides A–G (**1, 3**–**8**) were isolated from the gum resin of *Boswellia sacra*. Their structures were elucidated by extensive physicochemical and spectroscopic analysis, as well as ECD calculation, modified Mosher’s method and X-ray diffraction crystallography. Compounds **6** and **7** exhibited inhibitory activities on nitric oxide (NO) production induced by lipopolysaccharide in RAW264.7 cells with IC_50_ values of 24.9 ± 1.7 and 36.4 ± 2.9 *μ*M.

## Introduction

Cembranoids are a class of diterpenes biosynthesized from the cyclization of geranylgeranyl diphosphate to generate a 14-membered ring backbone decorated with a variety of oxidation patterns (Li and Pattenden, 2011). Natural occurrence of cembranoids has been found from both terrestrial and marine organisms, which showed not only a large structural diversity but also a wide range of biological activities (Yang et al., 2012). Plant-derived cembranoids have a relatively limited distribution, and have been reported mainly from tobacco (Yan et al., 2016), as well as the genera of *Croton*, *Euphorbia*, *Macaranga* (Euphorbiaceae), *Pinus* (Pinaceae), *Echinodorus* (Alismataceae), and *Boswellia* (Burseraceae), etc (Wahlberg et al., 1992; Yang et al., 2012).

Olibanum is an aromatic oleogum resin that exudes from incisions in the bark of *Boswellia* trees and has been used as incense and perfumes since antiquity. Olibanum has also been used in traditional medicines for the purpose of relieving pain and removing blood stasis. Cembranoids and triterpenes were reported as the bioactive constituents responsible for these effects (Banno et al., 2006; Takada et al., 2006; Al-Harrasi et al., 2019). *Boswellia sacra*, known as Olibanum-tree or Frankincense, is a small deciduous tree that is native to the Arabian Peninsula and northeastern Africa, and is one of the plants known to produce olibanum. Previous chemical investigations on the gum resin from *B. sacra* have reported the isolation of a number of cembranoids with neuroprotective, hepatoprotective, anti-inflammatory and anti-depression activities (Moussaieff et al., 2012; Pollastro et al., 2016; Wang et al., 2020).

As part of a continuing research for the discovery of bioactive natural products from medicinal plants (Xia et al., 2017; Zhang et al., 2020), a chemical investigation was carried out on the gum resin of *B. sacra*. Herein, we report the isolation and structural elucidation of nine cembranoids (**1**–**9**) as well as their inhibitory activities against lipopolysaccharide (LPS)-induced NO production in RAW 264.7 mouse monocyte-macrophages.

## Material and Methods

### General Experimental Procedures

Optical rotations were measured on a JASCO P-2200 polarimeter (JASCO Corp., Tokyo, Japan) in a 0.5 dm cell. The UV spectra were obtained with a Shimadzu UV 2201 spectrophotometer (Shimadzu Corp., Tokyo, Japan). The ECD spectra were measured on a JASCO J-1500 spectropolarimeter (JASCO Corp., Tokyo, Japan) in a 10 mm cell. The ^1^H and ^13^C NMR spectra were performed on Bruker AV-600 spectrometer (Bruker BioSpin, Zurich, Switzerland) or a JEOL ECA-500 spectrometer (JEOL Corp., Tokyo, Japan) with the measuring deuterated solvent as the internal reference. The chemical shifts are expressed in *δ* (ppm) and reported as s (singlet), d (doublet), t (triplet), dd (doublet of doublets), dt (doublet of triplets), ddd (doublet of doublet of doublets), ddt (doublet of doublet of triplets), hept (heptet), br (broad) and m (multiplet), respectively. HRESIMS was conducted using a Waters (Milford, MA) ACQUITY SYNAPTTM G2 high-definition mass spectrometer or a Q-Exactive Hybird Quadrupole Qrbitrap mass spectrometer (Thermo Electron Scientific Instrument Crop., WI, United States). Single-crystal X-ray diffraction measurements were conducted on a Bruker Smart Apex II diffractometer (Bruker BioSpin, Zurich, Switzerland) with a graphite monochromator. For preparative HPLC, a Waters 515 HPLC pump, equipped with a Shodex RI-101 Differential Refractometer detector and a JASCO UV-970 intelligent UV/VIS detector, was used. RP-HPLC separations were also conducted using an Shimadzu LC-6AD liquid chromatograph with a SPD-20A UV detector equipped with a YMC Pack ODS-A column (250 × 20 mm, 120 Å, 5 *μ*m). Silica gel GF_254_ (Qingdao Marine Chemical Factory, P. R. China) was used for TLC. Column chromatography (CC) was performed on Silica gel (200–300 mesh, Qingdao Marine Chemical Factory, P. R. China), Octadecyl silica gel (Merck Chemical Company Ltd., Germany), and Sephadex LH-20 (Amersham Pharmacia Biotech AB, Sweden). All reagents were of analytical grade (Concord Technology Co. Ltd., Tianjin, P. R. China).

### Plant Material

The gum resin of *Boswellia sacra* Flueck. syn. *Boswellia bhaw-dajiana* Birdw*.* originated in Ethiopia, was furnished by Tianjin Tongrentang Group Co., Ltd. The resin was authenticated by Professor Lin Ma (Tianjin University of Traditional Chinese Medicine). The voucher specimen (accession number: 11037Q) was deposited in the School of Chinese Materia Medica, Tianjin University of Traditional Chinese Medicine, P. R. China.

### Extraction and Isolation

The gum resin of *B. sacra* (6.8 kg) was powdered and extracted with 95% EtOH, then the extracts was evaporated to yield a residue (2.5 kg). The residue was separated by silica gel CC, and eluted sequentially with PE (petroleum ether), CH_2_Cl_2_ and MeOH. The CH_2_Cl_2_ fraction (513 g) was subjected to silica gel CC and eluted with a gradient of PE-EtOAc-MeOH to afford ten fractions (A-I).

Fraction B (12.9 g) was further subjected to an ODS CC, and eluted with a gradient of MeOH-H_2_O to afford subfractions B-1 to B-4. Subfraction B-3 (457 mg) was separated by preparative-HPLC with MeOH-H_2_O (9:1) to afford compound **5** (2.0 mg).

Fraction C (12.2 g) was subjected to ODS CC, and eluted with a gradient of MeOH-H_2_O to afford subfraction C-2. Subfraction C-2 (2.1 g) was subjected to Sephadex LH-20 CC and eluted with CH_2_Cl_2_-MeOH (1:1) to afford subfractions C-2-1 and C-2–2. Subfraction C-2-2 was separated by preparative-HPLC with MeOH-H_2_O (9:1) to afford compound **2** (15.4 mg).

Fraction D (23.5 g) was subjected to ODS CC and eluted with a gradient of MeOH-H_2_O to afford subfractions D-1 to D-7. Subfraction D-3 (651 mg) was separated by preparative-HPLC with MeCN-H_2_O (7:3) to afford compounds **9** (38.9 mg). Subfraction D-5 (1.2 g) was subjected to Sephadex LH-20 CC and eluted with CH_2_Cl_2_-MeOH (1:1), then the subfraction was separated by preparative-HPLC with MeCN-H_2_O (13:7) to afford compound **6** (4.0 mg). Subfraction D-6 (504 mg) was subjected to Sephadex LH-20 CC and eluted with CH_2_Cl_2_-MeOH (1:1), and then the subfraction was separated by preparative-HPLC with MeCN-H_2_O (7:3) to afford compound **4** (2.7 mg). Subfraction D-7 (336 mg) was separated by preparative-HPLC with MeCN-H_2_O (7:3) to afford compound **7** (2.5 mg).

Fraction F (18.4 g) was subjected to silica gel CC, and eluted with a gradient of PE-EtOAc to afford subfractions F-1 to F-3. Subfraction F-3 (1.1 g) was subjected to silica gel CC, eluted with PE-Acetone (4:1), and then separated by preparative-HPLC with MeOH-H_2_O (17:3) to afford compound **3** (11.3 mg).

Fraction H (8.3 g) was subjected to ODS CC, and eluted with a gradient of MeOH-H_2_O to afford subfractions H-1 and H-2. Subfraction H-2 (1.3 g) was subjected to Sephadex LH-20 CC, eluted with MeOH, and then separated by preparative-HPLC with MeCN-H_2_O (9:11) to afford compound **8** (9.4 mg).

Fraction I (10.6 g) was subjected to ODS CC and eluted with a gradient of MeOH-H_2_O to afford subfractions I-1 to I-3. Subfraction I-2 (2.5 g) was subjected to Sephadex LH-20 CC, eluted with CH_2_Cl_2_-MeOH (1:1), and then separated by preparative-HPLC with MeOH-H_2_O (7:3) to afford compound **1** (17.6 mg).

Sacraoxide A (**1**), colorless needles; mp 161–162°C [*α*]^25^_D_ +15.0 (*c* 0.12, MeOH); UV (MeOH) *λ*_max_ (log *ε*) 201 (3.88) nm; ECD (*c* 1.6 × 10^-4^ M, MeOH) *λ*_max_ (Δ*ε*) 204 (+30.89) nm; IR (film) *ν*_max_ 3357, 2959, 2921, 1655, 1448, 1383, 1089, 1061, 1033, 1021, 928, 892 cm^−1^; ^1^H and ^13^C NMR spectroscopic data, see Tables 1, 2; HRESIMS *m*/*z* 323.2574 [M + H]^+^ (calcd for C_20_H_35_O_3_, 323.2581).

**TABLE 1:** ^1^H NMR spectroscopic data of compounds **1**, **3**–**5** (*δ* in ppm, and *J* in Hz, in CDCl_3_).

Position	1^a^	3^b^	4^b^	5^b^
2	2.38, dd, (15.8, 8.5), *β*	2.52, dd, (14.4, 10.2), *β*	2.63, d (14.4), *β*	2.91, d, (12.6), *β*
	1.93^c^, *α*	1.89, dd, (14.4, 4.8), *α*	2.57, d (14.4), *α*	2.34, d, (12.6), *α*
3	5.27, dd, (8.5, 7.4)	5.40, dd, (10.2, 4.8)		
4			2.56, m, *α*	2.91, m, *α*
5	2.22, ddd, (14.0, 11.5, 2.3), *α*	2.39, td, (13.2, 3.6), *α*	1.87, m, *α*	1.65^c^, *β*
	2.05^c^, *β*	2.08, brd, (13.2), *β*	1.35, m, *β*	1.23, m, *β*
6	1.74, ddd (11.5, 7.8, 1.4), *β*	1.70, ddd, (13.2, 9.6, 3.6), *α*	2.12, m, *α*	2.19, m, *α*
	1.53, ddt, (14.0, 11.2, 3.2), *α*	1.36, ddd, (13.2, 13.2, 1.2), *β*	2.10, m, *β*	2.08^c^, *β*
7	4.63, dd, (7.8, 3.2)	4.93, dd, (9.6, 1.2)	5.08, t, (7.2)	5.42, dd, (9.6, 6.0)
9	5.34, t, (6.6)	5.09, ddd, (9.6, 5.4, 1.2)	2.24, m, *α*	1.98, m, *α*
			1.97, m, *β*	1.80, td, (13.8, 1.8), *β*
10	2.30, ddd, (15.8, 7.2, 2.6), *α*	2.53^c^, *α*	1.99, m, *α*	1.68, m, *α*
	2.09, dd, (15.8, 11.2), *β*	1.88, dd, (14.4, 12.0), *β*	1.42, m, *β*	1.65^c^, *β*
11	3.45, dd, (11.2, 2.6), *α*	5.13, dd, (12.0, 1.8), *α*	3.44, d, (10.2), *α*	5.13, d, (9.0), *α*
13	1.95, m, *α*	1.72, m, *α*	1.97, m, *α*	1.94, m, *α*
	1.78, m, *β*	1.69, m, *β*	1.80, m, *β*	1.60, m, *β*
14	1.83, m, *α*	2.01, td, (12.0, 7.8), *α*	1.48, m, *β*	1.95, m, *β*
	1.78, m, *β*	1.81, m, *β*	1.37, m, *α*	1.52, m, *α*
15	1.86, m	1.84, m	2.40, hept, (7.2)	1.61, m
16	0.98, d, (6.9)	0.88, d, (7.2)	0.87, d, (6.6)	0.91, d, (6.6)
17	0.86, d, (7.2)	1.00, d, (7.2)	0.90, d, (6.6)	0.87, d, (6.6)
18	1.60, s	1.59, s	1.04, d, (7.2)	1.03, d, (6.0)
19	1.70, s	1.65, s	1.67, s	1.66, s
20	1.22, s	1.22, s	1.09, s	1.11, s
Ac-2′		2.03, s		2.09, s

^a^ Recorded at 500 MHz.

^b^ Recorded at 600 MHz.

^c^ Overlapping resonances.

**TABLE 2:** ^13^C NMR spectroscopic data of compounds **1**, **3**–**8** (*δ* in ppm, in CDCl_3_).

Position	1^a^	3^b^	4^b^	5^b^	6^b^	7^b^	8^a^
1	88.5, C	89.5, C	88.9, C	89.6, C	89.3, C	89.2, C	88.2, C
2	28.5, CH_2_	28.1, CH_2_	47.3, CH_2_	49.6, CH_2_	34.0, CH_2_	33.1, CH_2_	128.8, CH
3	121.9, CH	121.8, CH	213.4, C	215.7, C	126.7, C	123.2, CH	137.6, CH
4	137.0, C	137.1, C	46.5, CH	42.5, CH	131.5, C	134.4, C	74.4, C
5	35.6, CH_2_	36.2, CH_2_	30.8, CH_2_	33.4, CH_2_	57.1, CH_2_	38.5, CH_2_	43.8, CH_2_
6	32.6, CH_2_	30.5, CH_2_	25.2, CH_2_	24.6, CH_2_	201.2, C	24.6, CH_2_	24.3, CH_2_
7	68.3, CH	66.1, CH	124.6, CH	125.8, CH	120.3, CH	143.7, CH	128.5, CH
8	140.7, C	143.6, C	135.6, C	135.8, C	154.8, C	135.7, C	133.6, C
9	121.9, CH	118.8, CH	32.5, CH_2_	34.1, CH_2_	33.8, CH_2_	202.2, C	35.2, CH_2_
10	28.1, CH_2_	25.5, CH_2_	31.8, CH_2_	29.1, CH_2_	25.8, CH_2_	40.6, CH_2_	29.6, CH_2_
11	76.9, CH	77.2, CH	77.3, CH	78.2, CH	77.2, CH	78.2, CH	76.2, CH
12	84.1, C	82.6, C	85.5, C	84.7, C	83.5, C	83.2, C	84.6, C
13	35.2, CH_2_	35.3, CH_2_	36.5, CH_2_	35.3, CH_2_	35.6, CH_2_	35.8, CH_2_	36.6, CH_2_
14	31.2, CH_2_	29.6, CH_2_	31.0, CH_2_	31.4, CH_2_	30.5, CH_2_	30.3, CH_2_	34.9, CH_2_
15	35.2, CH	35.3, CH	33.1, CH	35.2, CH	33.4, CH	33.1, CH	35.2, CH
16	16.1, CH_3_	19.3, CH_3_	18.7, CH_3_	18.2, CH_3_	18.8, CH_3_	18.5, CH_3_	18.5, CH_3_
17	19.1, CH_3_	16.1, CH_3_	17.3, CH_3_	17.2, CH_3_	17.5, CH_3_	17.4, CH_3_	17.5, CH_3_
18	15.8, CH_3_	15.4, CH_3_	16.8, CH_3_	14.2, CH_3_	16.3, CH_3_	14.8, CH_3_	29.2, CH_3_
19	17.0, CH_3_	16.9, CH_3_	19.3, CH_3_	17.0, CH_3_	20.6, CH_3_	11.5, CH_3_	16.6, CH_3_
20	23.6, CH_3_	22.3, CH_3_	19.6, CH_3_	21.2, CH_3_	21.3, CH_3_	21.6, CH_3_	20.0, CH_3_
Ac-1′		171.8, C		171.3, C	171.0, C	170.4, C	
Ac-2′		21.3, CH_3_		21.3, CH_3_	21.1, CH_3_	21.0, CH_3_	

^a^ Recorded at 125 MHz.

^b^ Recorded at 150 MHz

Sacraoxide B (**3**), colorless oil [*α*]^25^_D_ −4.1 (*c* 0.10, MeOH); UV (MeOH) *λ*_max_ (log *ε*) 202 (3.31) nm; ECD (*c* 1.6 × 10^-4^ M, MeOH) *λ*_max_ (Δ*ε*) 195 (−5.62), 206 (+9.27) nm; IR (film) *ν*_max_ 3467, 2958, 2928, 1726, 1448, 1372, 1236, 1099, 1027, 925, 890 cm^−1^; ^1^H and ^13^C NMR spectroscopic data, see Tables 1, 2; HRESIMS *m*/*z* 387.2513 [M + Na]^+^ (calcd for C_22_H_36_O_4_Na, 387.2511).

Sacraoxide C (**4**), colorless oil [*α*]^25^_D_ +10.4 (*c* 0.10, MeOH); UV (MeOH) *λ*_max_ (log *ε*) 201 (3.18) nm; ECD (*c* 7.8 × 10^-5^ M, MeOH) *λ*_max_ (Δ*ε*) 205 (+1.02), 239 (−0.04), 292 (+0.49) nm; IR (film) *ν*_max_ 3417, 2965, 2932, 2875, 1735, 1732, 1457, 1372, 1310, 1239, 1025, 1023, 923, 886 cm^−1^; ^1^H and ^13^C NMR spectroscopic data, see Tables 1, 2; HRESIMS *m*/*z* 323.2581 [M + H]^+^ (calcd for C_20_H_35_O_3_, 323.2586); *m/z* 345.2401 [M + Na]^+^ (calcd for C_20_H_34_O_3_Na, 345.2406).

Sacraoxide D (**5**), pale yellow oil [*α*]^25^_D_ +47.7 (*c* 0.10, MeOH); UV (MeOH) *λ*_max_ (log *ε*) 201 (3.09) nm; ECD (*c* 6.8 × 10^-5^ M, MeOH) *λ*_max_ (Δ*ε*) 207 (+1.48), 237 (−0.00), 295 (+2.00) nm; IR (film) *ν*_max_ 2965, 2931, 2875, 1732, 1728, 1457, 1372, 1238, 1110, 1023, 918, 886 cm^−1^; ^1^H and ^13^C NMR spectroscopic data, see Tables 1, 2; HRESIMS *m*/*z* 387.2515 [M + Na]^+^ (calcd for C_22_H_36_O_4_Na, 387.2511).

Sacraoxide E (**6**), colorless oil [*α*]^25^_D_ −64.0 (*c* 0.10, MeOH); UV (MeOH) *λ*_max_ (log *ε*) 201 (3.38), 234 (3.08) nm; ECD (*c* 6.9 × 10^-5^ M, MeOH) *λ*_max_ (Δ*ε*) 203 (+0.60), 241 (−2.01), 280 (+0.02), 348 (−0.72) nm; IR (film) *ν*_max_ 2960, 2926, 1733, 1684, 1373, 1236, 1240, 1097, 1039, 976, 891 cm^−1^; ^1^H and ^13^C NMR spectroscopic data, see Tables 2, 3; HRESIMS *m*/*z* 385.2354 [M + Na]^+^ (calcd for C_22_H_34_O_4_Na, 385.2355).

**TABLE 3:** ^1^H NMR spectroscopic data of compounds **6**–**8** (*δ* in ppm, and *J* in Hz, in CDCl_3_).

Position	6^a^	7^a^	8^b^
2	2.30, dd, (12.6, 4.2), *β*	2.29, dd, (12.6, 4.2), β	5.54, d, (15.4)
	1.85, dd, (12.6, 7.2), *α*	2.10, dd, (12.6, 5.4), α	
3	5.49, dd, (7.2, 4.2)	5.35, dd, (5.4, 4.2)	5.81, d, (15.4)
5	3.10, d, (14.4), *α*	2.31^c^, α	1.82^c^, α
	2.92, d, (14.4), *β*	2.30^c^, β	1.57, td, (11.4, 1.4), β
6		2.51, dd, 1 (2.6, 9.6), α	2.23, ddd, (15.2, 4.2, 1.4), α
		2.27, dd, (12.6, 1.8), β	2.15, dd, (15.2, 10.2), β
7	6.53, s	6.68, dd, (9.6, 1.8)	5.16, dd, (10.2, 4.2)
9	2.27^c^, *α*		2.07, m
	1.82, dd, (12.6, 7.2), *β*		
10	2.10, d, (14.4), *α*	3.60, dd, (12.6, 0.6), α	1.93, ddd, (15.8, 7.4, 1.8), α
	1.60^c^, *β*	2.25, dd, (12.6, 10.8), β	1.31, dd, (15.8, 9.8), β	
11	4.84, d, (10.8), *α*	4.87, dd, (10.8, 0.6), α	3.47, d, (9.8), α
13	1.88, m, *α*	1.82, ddd, (12.6, 10.8, 4.2), α	1.81, m, α
	1.64, m, *β*	1.69^c^, β	1.72, m, β
14	1.89, m, *β*	1.90, ddd, (12.6, 10.8, 4.2), β	1.88, dd, (11.2, 7.4), β
	1.49, m, *α*	1.53, ddd, (12.6, 7.8, 4.2), α	1.83, m, α	
15	2.10, m	2.21, hept, (7.2)	1.64, hept, (6.9)
16	0.93, d, (6.6)	0.91, d, (6.6)	0.82, d, (7.1)
17	0.96, d, (6.6)	0.94, d, (6.6)	0.79, d, (6.5)
18	1.60, s	1.57, s	1.27, s
19	2.05, s	1.69, s	1.66, s
20	1.12, s	1.13, s	1.04, s
Ac-2′	2.05, s	1.99, s	

^a^ Recorded at 600 MHz.

^b^ Recorded at 500 MHz.

^c^Overlapping resonances.

Sacraoxide F (**7**), pale yellow oil [*α*]^25^_D_ +10.2 (*c* 0.10, MeOH); UV (MeOH) *λ*_max_ (log *ε*) 201 (3.50), 226 (3.10) nm; ECD (*c* 7.8 × 10^-5^ M, MeOH) *λ*_max_ (Δ*ε*) 233 (−2.85), 287 (+0.18) nm; IR (film) *ν*_max_ 2960, 2929, 1733, 1684, 1374, 1236, 1028, 981, 854 cm^−1^; ^1^H and ^13^C NMR spectroscopic data, see Tables 2, 3; HRESIMS *m*/*z* 363.2504 [M + H]^+^ (calcd for C_22_H_35_O_4_, 363.2535).

Sacraoxide G (**8**), white amorphous powder [*α*]^25^_D_ +115.0 (*c* 0.11, MeOH); UV (MeOH) *λ*_max_ (log *ε*) 201 (3.29) nm; ECD (*c* 3.1 × 10^-4^ M, MeOH) *λ*_max_ (Δ*ε*) 203 (+22.82) nm; IR (film) *ν*_max_ 3358, 2919, 2849, 1658, 1632, 1469, 1382, 1075, 977, 944 cm^−1^; ^1^H and ^13^C NMR spectroscopic data, see Tables 2, 3; HRESIMS *m*/*z* 305.2583 [M − H_2_O + H]^+^ (calcd for C_20_H_33_O_2_, 305.2475).

Boscartins AD (**2**), colorless oil; ^1^H NMR (600 MHz, CDCl_3_) *δ* 2.41 (1H, dd, *J* = 15.6, 6.6 Hz, H-2*β*), 1.91 (1H, dd, *J* = 15.6, 4.8 Hz, H-2*α*), 5.11 (1H, dd, *J* = 6.6, 4.8 Hz, H-3), 2.15 (1H, dd, *J* = 14.4, 9.0 Hz, H-5*α*), 1.94 (1H, brd, *J* = 14.4 Hz, H-5*β*), 1.83 (1H, overlap, H-6*α*), 1.62 (1H, overlap, H-6*β*), 4.72 (1H, t, *J* = 6.6 Hz, H-7), 5.57 (1H, dd, *J* = 12.0, 4.2 Hz, H-9), 2.93 (1H, dd, *J* = 13.2, 12.0 Hz, H-10*α*), 1.69 (1H, ddd, *J* = 13.2, 10.8, 4.2 Hz, H-10*β*), 3.20 (1H, d, *J* = 10.8 Hz, H-11), 2.07 (1H, m, H-13*α*), 1.74 (1H, m, H-13*β*), 1.80 (2H, m, H-14), 1.83 (1H, m, H-15), 0.97 (3H, d, *J* = 6.6 Hz, H_3_-16), 0.88 (3H, d, *J* = 7.2 Hz, H_3_-17), 1.62 (3H, s, H_3_-18), 1.74 (3H, s, H_3_-19), 1.16 (3H, s, H_3_-20); ^13^C NMR (150 MHz, CDCl_3_) *δ* 88.2 (C-1), 30.1 (C-2), 121.2 (C-3), 134.2 (C-4), 34.7 (C-5), 31.3 (C-6), 66.9 (C-7), 138.0 (C-8), 125.6 (C-9), 29.0 (C-10), 78.4 (C-11), 84.3 (C-12), 35.4 (C-13), 30.9 (C-14), 35.8 (C-15), 17.1 (C-16), 18.8 (C-17), 16.4 (C-18), 17.1 (C-19), 20.8 (C-20); HRESIMS *m*/*z* 305.2471 [M − H_2_O + H]^+^ (calcd for C_20_H_33_O_2_, 305.2475).

Boscartins L (**10**), pale yellow oil; ^1^H NMR (600 MHz, CDCl_3_) *δ* 2.42 (1H, dd, *J* = 16.2, 7.2 Hz, H-2*β*), 1.89 (1H, overlap, H-2*α*), 5.22 (1H, dd, *J* = 7.2, 5.4 Hz, H-3), 2.15 (1H, m, H-5*β*), 1.98 (1H, m, H-5*α*), 1.82 (1H, m, H-6*α*), 1.68 (1H, overlap, H-6*β*), 4.68 (1H, t, *J* = 6.6 Hz, H-7), 5.18 (1H, dd, *J* = 11.4, 4.2 Hz, H-9), 2.90 (1H, dd, *J* = 13.2, 11.4 Hz, H-10*α*), 1.88 (1H, ddd, *J* = 13.2, 11.4, 4.2 Hz, H-10*β*), 4.72 (1H, d, *J* = 11.4 Hz, H-11), 1.78 (1H, m, H-13*α*), 1.64 (1H, m, H-13*β*), 1.86 (1H, m, H-14*β*), 1.81 (1H, m, H-14*α*), 1.82 (1H, m, H-15), 0.88 (3H, d, *J* = 7.2 Hz, H_3_-16), 0.96 (3H, d, *J* = 7.2 Hz, H_3_-17), 1.65 (3H, s, H_3_-18), 1.68 (3H, s, H_3_-19), 1.18 (3H, s, H_3_-20), 2.09 (3H, s, H_3_-2′); ^13^C NMR (150 MHz, CDCl_3_) *δ* 88.7 (C-1), 30.3 (C-2), 121.1 (C-3), 134.6 (C-4), 34.9 (C-5), 31.3 (C-6), 67.1 (C-7), 138.1 (C-8), 124.1 (C-9), 26.6 (C-10), 78.0 (C-11), 83.3 (C-12), 35.0 (C-13), 30.9 (C-14), 36.0 (C-15), 18.8 (C-16), 17.1 (C-17), 16.1 (C-18), 17.0 (C-19), 21.8 (C-20), 171.1 (C-1′), 21.3 (C-2′); HRESIMS *m*/*z* 387.2506 [M + Na]^+^ (calcd for C_22_H_36_O_4_Na, 387.2511).

### Crystal Structure Determination of 1

Crystal Data for C_20_H_36_O_4_ (*M* = 340.49 g/mol): monoclinic, space group I2 (no. 5), *a* = 23.3651 (6) Å, *b* = 10.1181 (2) Å, *c* = 67.6869 (15) Å, *β* = 91.113 (2), *V* = 15,998.9 (6) Å3, *Z* = 32, *T* = 100.01 (11) K, *μ*(Cu Kα) = 0.608 mm^-1^, *Dcalc* = 1.131 g/cm^3^, 58,130 reflections measured (4.026 ≤ 2*θ* ≤ 148.16), 25,373 unique (*R*_int_ = 0.1124, *R*_sigma_ = 0.1243) which were used in all calculations. The final R_1_ was 0.0818 [I > 2σ(I)] and *wR*_2_ was 0.2253 (all data).

### Preparation of (R)- and (S)-MTPA Esters of 2

As described in previous literature (Li et al., 2007), compound **2** (2.0 mg) was dissolved in anhydrous pyridine (0.5 ml) and transferred into a dried bottle, and then 10 *μ*L of *S*-(‒)-*α*-methoxy-*α*-(trifluoromethyl) phenylacetyl chloride (*S*-MTPA-Cl; Sigma-Aldrich Co., St. Louis, MO, United States) was added. After stirring at room temperature for 24 h, the reaction mixture was evaporated to give a residue, which was purified by semipreparative HPLC (MeOH-H_2_O 9:1, v/v, 3 ml/min) to give the *R*-MTPA ester derivative of **2** (**2a**, *t*_R_ = 10.2 min, 2.1 mg). The *S*-MTPA ester derivative of **2** (**2b**, 1.8 mg) was obtained by using the same procedure as described above but using *R*-MTPA-Cl.

(*R*)-MTPA ester of **2** (**2a**), colorless oil; ^1^H NMR (600 MHz, CDCl_3_) *δ* 2.45 (1H, dd, *J* = 15.6, 7.2 Hz, H-2a), 1.87 (1H, overlap, H-2b), 5.16 (1H, dd, *J* = 7.2, 5.4 Hz, H-3), 1.97 (1H, m, H-5a), 1.94 (1H, m, H-5b), 1.97 (1H, m, H-6a), 1.70 (1H, m, H-6b), 5.96 (1H, dd, *J* = 7.2, 6.6 Hz, H-7), 5.72 (1H, dd, *J* = 12.0, 3.6 Hz, H-9), 3.14 (1H, dd, *J* = 13.2, 12.0 Hz, H-10a), 1.77 (1H, overlap, H-10b), 3.19 (1H, d, *J* = 10.8 Hz, H-11), 2.05 (1H, m, H-13a), 1.75 (1H, m, H-13b), 1.83 (2H, m, H-14), 1.81 (1H, m, H-15), 1.00 (3H, d, *J* = 7.2 Hz, H_3_-16), 0.89 (3H, d, *J* = 6.6 Hz, H_3_-17), 1.62 (3H, s, H_3_-18), 1.69 (3H, s, H_3_-19), 1.17 (3H, s, H_3_-20); HRESIMS *m*/*z* 561.2809 [M + Na]^+^ (calcd for C_30_H_41_O_5_F_3_Na, 561.2804).

(*S*)-MTPA ester of **2** (**2b**), colorless oil; ^1^H NMR (600 MHz, CDCl_3_) *δ*: 2.45 (1H, dd, *J* = 16.2, 7.2 Hz, H-2a), 1.88 (1H, dd, *J* = 16.2, 5.4 Hz, H-2b), 5.16 (1H, dd, *J* = 7.2, 5.4 Hz, H-3), 2.07 (1H, m, H-5a), 1.99 (1H, m, H-5b), 2.05 (1H, m, H-6a), 1.78 (1H, m, H-6b), 5.91 (1H, dd, *J* = 6.6, 6.0 Hz, H-7), 5.69 (1H, dd, *J* = 12.0, 3.6 Hz, H-9), 3.14 (1H, dd, *J* = 13.2, 12.0 Hz, H-10a), 1.78 (1H, ddd, *J* = 13.2, 10.8, 4.2 Hz, H-10b), 3.19 (1H, d, *J* = 10.8 Hz, H-11), 2.06 (1H, m, H-13a), 1.74 (1H, m, H-13b), 1.83 (2H, m, H-14), 1.81 (1H, m, H-15), 0.99 (3H, d, *J* = 6.6 Hz, H_3_-16), 0.89 (3H, d, *J* = 7.2 Hz, H_3_-17), 1.50 (3H, s, H_3_-18), 1.63 (3H, s, H_3_-19), 1.17 (3H, s, H_3_-20); HRESIMS *m*/*z* 561.2804 [M + Na]^+^ (calcd for C_30_H_41_O_5_F_3_Na, 561.2804).

### ECD Calculations

The details of the quantum chemical ECD calculations for compounds **4, 6-8** are provided in Supplementary data.

### NO Inhibitory Assay

The RAW 264.7 (ATCCTIB-71) mouse monocyte-macrophages were cultured in RPMI 1640 medium supplemented with penicillin G (100 units/mL), streptomycin (100 mg/ml) and 10% FBS. The cells were seeded in 96-well plastic plates with 1 × 10^5^cells/well and allowed to adhere for 24 h at 37°C in a humidified atmosphere containing 5% CO_2_. Then the medium was replaced with fresh medium, containing LPS (1 *μ*g/ml) together with test compounds at various concentrations and then incubated for 24 h. NO production was determined by measuring the accumulation of nitrite in the culture supernatant using Griess reagent. Briefly, 100 *μ*L of the supernatant from incubates were mixed with equal volume of Griess reagent (1% sulfanilamide and 0.1% naphthylene-diamide dihydrochloride in 2.5% H_3_PO_4_) and were allowed to stand for 10 min at 37°C in a humidified atmosphere containing 5% CO_2_. Absorbance at 540 nm was measured using microplate reader. The nitrite concentrations were calculated according to the literature (Jin et al., 2016).

### Cell Viability

Cell viability was determined using the mitochondrial respiration-dependent MTT reduction method. After transferring the required supernatant to another plate for the Griess assay, the remaining supernatant was aspirated from the 96-well plates, 100 *μ*L of fresh medium and 10 *μ*L of MTT (5 mg/ml PBS) were added to each well. The cells were incubated at 37°C in a humidified atmosphere containing 5% CO_2_. After incubating for 4 h, the medium was removed and the violet crystals of formazan in viable cells were dissolved in DMSO. Absorbance at 570 nm was measured using a microplate reader.

## Results

**FIGURE 1:**
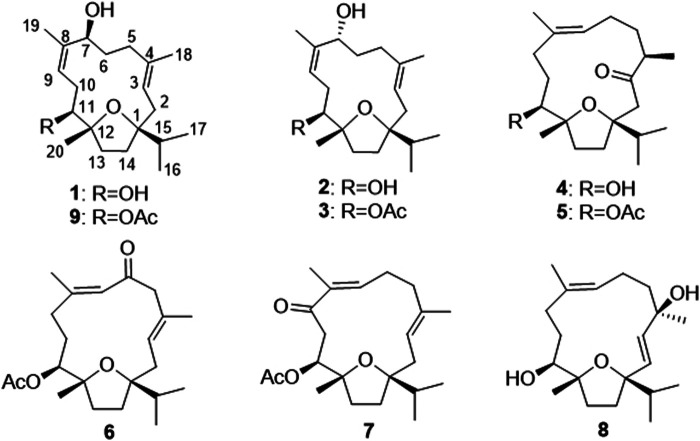
Chemical structures of compounds **1**–**9**.

A 95% EtOH extract of the gum resin of *B. sacra* was separated by multiple column chromatography (CC) including silica gel, Sephadex LH-20 and ODS CC, as well as preparative HPLC to afford nine cembranoids (**1**–**9**, Figure 1). The known compounds were identified as boscartin AD (**2**) and boscartin L (**9**) by detailed spectroscopic and physicochemical analyses and comparison of literature data (Wang J. J. et al., 2019; Wang Y. G. et al., 2019). The new compounds were named as sacraoxides A–G (**1**, **3**–**8**), and their structures were elucidated as follows.

**FIGURE 2:**
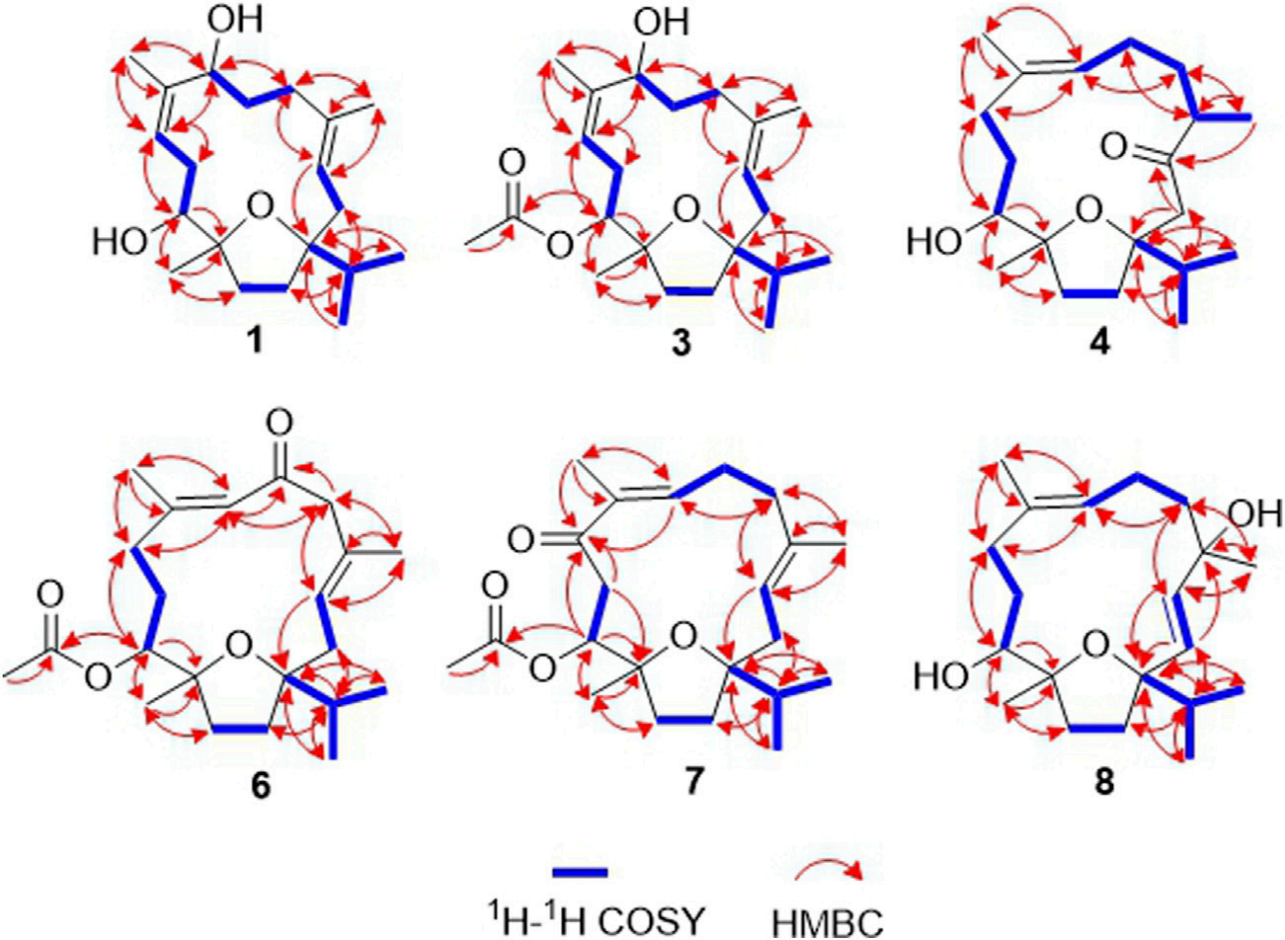
Key ^1^H–^1^H COSY and HMBC correlations of compounds **1**, **3**–**4**, **6**–**8**.

Sacraoxide A (**1**) was isolated as colorless needles (MeOH-H_2_O) [*α*]^25^_D_ +15.0 (*c* 0.12, MeOH). The molecular formula of **1** was established as C_20_H_34_O_3_ from a protonated molecule at *m/z* 323.2574 [M + H]^+^ (calculated for C_20_H_35_O_3_, 323.2581) in the HRESIMS spectrum. The cembranoid skeleton of **1** was indicated by the characteristic resonances for an isopropyl moiety at *δ*_H_ 1.86 (m, H-15), 0.98 (d, *J* = 6.9 Hz, H_3_-16) and 0.86 (d, *J* = 7.2 Hz, H_3_-17) in the ^1^H NMR spectrum (Table 1), as well as their corresponding carbon resonances at *δ*_C_ 35.2 (C-15), 16.1 (C-16) and 19.1 (C-17) in the ^13^C NMR spectrum (Table 2) (Wang, et al., 2020). Compound **1** showed superimposable ^1^H and ^13^C NMR resonances as the known compound **9**, but lacked resonances for an acetyl moiety. In the ^13^C NMR spectrum of **1**, two oxygenated tertiary carbons resonances at *δ*_C_ 88.5 (C-1) and 84.1 (C-12) were assignable to a tetrahydrofuran structure formed by cyclization between C-1 and C-12 through an ether bond, which was indicated by the HMBC correlations between H-15/C-14, H-15/C-1, H_3_-20/C-12 and H_3_-20/C-13 (Figure 2) (Wang J. J. et al., 2019; Wang Y. G. et al., 2019; Wang et al., 2020). Further functionalities were suggested to be two tri substituted olefins [*δ*_H_ 5.27 (H-3), *δ*_C_ 121.9 (C-3), 130.7 (C-4); *δ*_H_ 5.34 (H-9), *δ*_C_ 121.9 (C-9), 140.7 (C-8)] and two oxygenated methines [*δ*_H_ 4.63 (H-7), *δ*_C_ 68.3 (C-7); *δ*_H_ 3.45 (H-11), *δ*_C_ 76.9 (C-11)]. The assignment of a ∆^3,4^ olefin moiety in **1** was accomplished by the HMBC correlations between H-3/C-1, H-3/C-18, and H_3_-18/C-3, while a hydroxy group at C-11 was evident by the HMBC correlations between H-11/C-20 and H_3_-20/C-11. On the other hand, the ∆^8,9^ olefin and the second hydroxy group at C-7 were evident by the HMBC correlations between H-11/C-9, H-9/C-11, H_3_-19/C-9, H_3_-19/C-7, H-7/C-19, and H-9/C-19. Thus, the planar structure of compound **1** was concluded as 1:12-epoxy-cembra-3,8-dien-7,11-diol.

**FIGURE 3:**
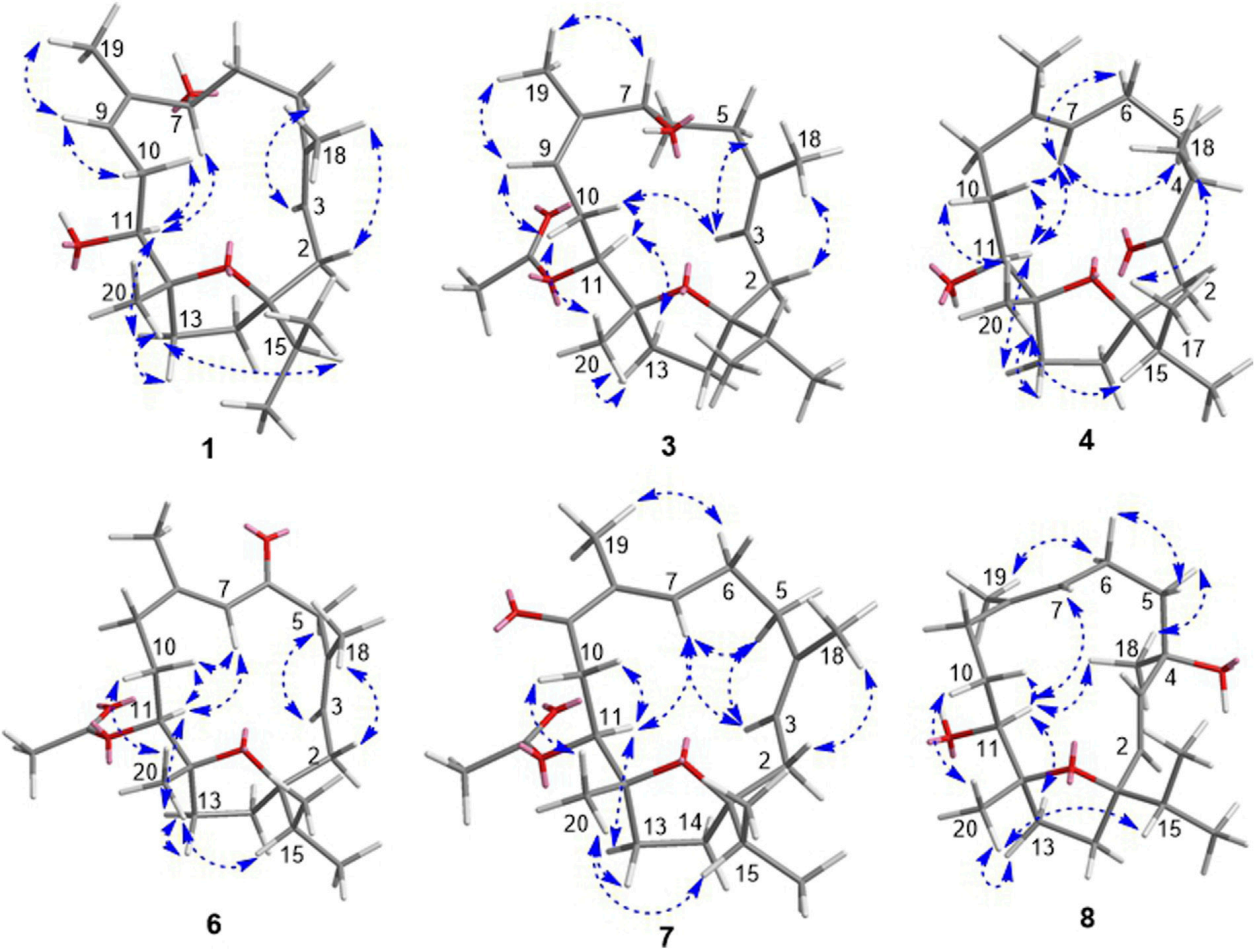
Key NOESY correlations of compounds **1**, **3**–**4**, **6**–**8**.

**FIGURE 4:**
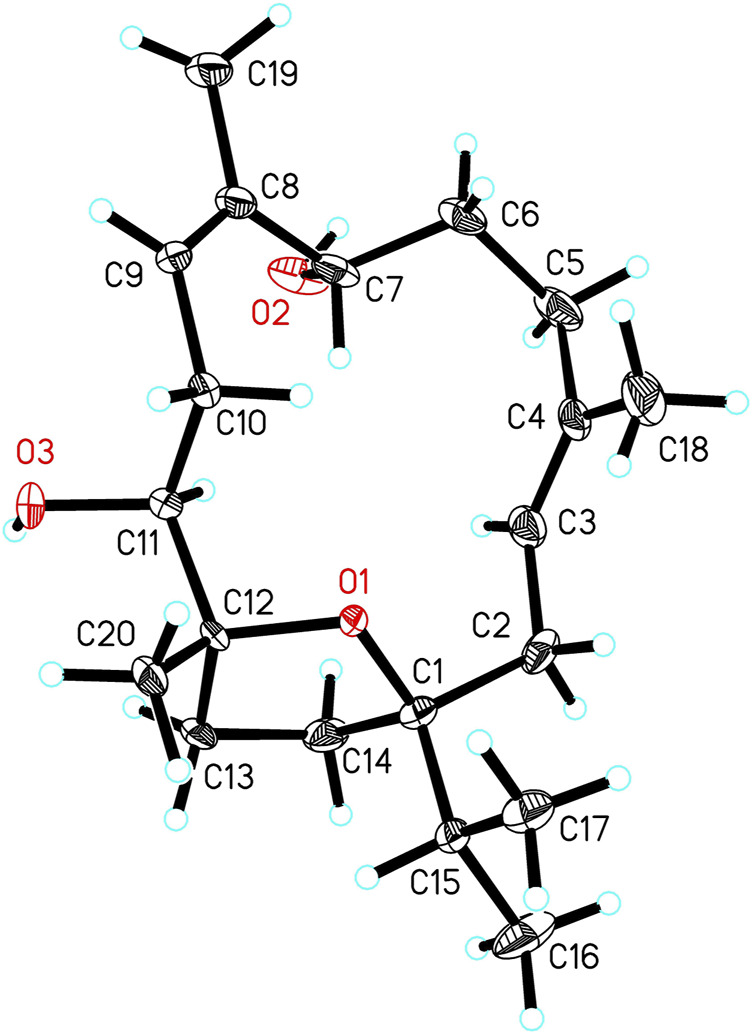
X-ray ORTEP drawing of compound **1**.

The relative configurations were elucidated by interpretation of the NOESY data (Figure 3). The *cis* relationship between C-1 isopropyl moiety and C-12 methyl moiety was evident by the NOESY correlation between H-15/H_3_-20. The *β*-orientation of OH-11 was deduced by the NOESY correlations between H_3_-20/H-13*β*, and H-11/H-13*α*, while the *β*-orientation of OH-7 was deduced by the NOESY correlation between H-7/H-11. The 3*E*, 8*Z* configurations of the olefinic geometries were determined by NOESY correlations between H-3/H-5*α*, H_3_-18/H-2*β* and H-9/H_3_-19. Finally, the structure of **1** including the absolute configurations was determined by single-crystal X-ray diffraction analysis using Cu K*α* radiation [CDCC number, 2035706, Flack parameter = −0.2 (2)] (Figure 4). Thus, the structure of sacraoxide A (**1)** was unambiguously determined as (1*S*,7*S*,11*S*,12*R*)-1:12-epoxy-cembra-3*E*,8*Z*-dien-7,11-diol.

Sacraoxide B (**3**) was isolated as colorless oil [*α*]^25^_D_ −4.1 (*c* 0.10, MeOH). The molecular formula of **3** was established as C_22_H_36_O_4_ from the HRESIMS positive-ion at *m/z* 387.2513 [M + Na]^+^ (calculated for C_22_H_36_O_4_Na, 387.2511). The 1D and 2D NMR spectroscopic data of **3** were mostly compatible with those of the known compound **2**, except for the downfield shifted H-11 resonance (*δ*_H_ 5.13) and the existence of a set of resonances for an acetyl group [*δ*_H_ 2.03 (H_3_-2′), *δ*_C_ 21.3 (C-2′), 171.8 (C-1′)] (Tables 1, 2). The position of the acetyl group at C-11 was further determined by the HMBC correlation between H-11/C-1′. In the NOESY spectrum, a key correlation was observed between H-7/H_3_-19 but not of H-7/H-11, indicating that OH-7 is *α*-orientated (Figure 3). Meanwhile, the ECD spectra of **3** and **2** also revealed high similarities, suggesting the same absolute configurations.

**FIGURE 5:**
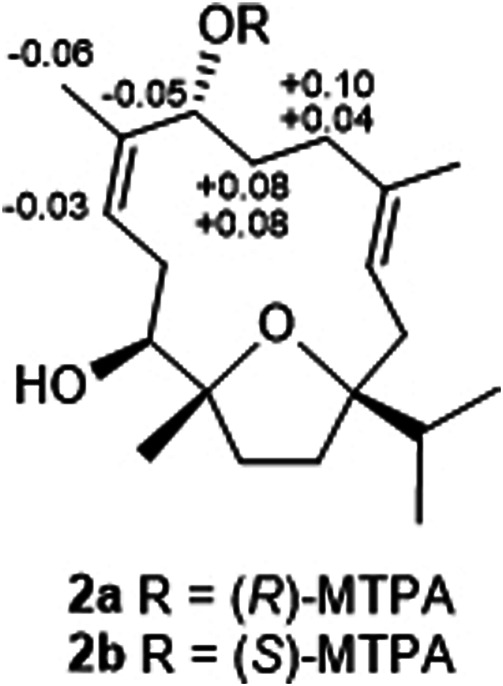
Results of compound **2** with the modified mosher’s method (Δ*δ*_H_ = *δ*_S_ − *δ*_R_).

In order to determine the absolute configurations of compound **3**, the modified Mosher’s method was carried out (Li et al., 2007). Compound **2** was separately esterified with (*S*) and (*R*)-MTPA chloride to give corresponding (*R*) and (*S*)-MTPA esters, **2a** and **2b**, respectively. As a result, only mono-substituted (*R/S*)-MTPA esters were obtained as the products, which were demonstrated by the HRESIMS and NMR spectroscopic data. The fact that OH-7 was esterified, was indicated by the downfield shifted H-7 resonances and the HMBC correlations between H-7/MTPA-C-1′ in **2a** and **2b**. The regioselectivity was in accordance with *cis*-relationship between CH_3_-20 and OH-11 causing steric hindrance effect. The distribution of Δ*δ* values between **2b** and **2a** indicated the 7*R*-configuration of **2** (Figure 5). Thus, the structure of sacraoxide B (**3**) was determined as (1*S*,7*R*,11*S*,12*R*)-1:12-epoxy-11-acetoxy-cembra-3*E*,8*Z*-dien-7-ol.

Sacraoxide C (**4**) was isolated as colorless oil [*α*]^25^_D_ +10.4 (*c* 0.10, MeOH). The molecular formula of **4** was established as C_20_H_34_O_3_ from a protonated molecule at *m/z* 323.2581 [M + H]^+^ (calculated for C_20_H_35_O_3_, 323.2586) in the HRESIMS spectrum. Compound **4** is a cembranoid possessing a tetrahydrofuran ring and 11-hydroxy group, which was deduced by the same NMR spectroscopic data analysis process as aforementioned in compound **1**. However, in the ^1^H and ^13^C NMR spectra of **4**, the resonances of a methyl doublet at *δ*_H_ 1.04 (d, *J* = 7.2 Hz, H_3_-18), and a carbonyl group at *δ*_C_ 213.4 (C-3) were observed (Tables 1, 2). Their positions were assigned by the HMBC correlations between H-15/C-2, H_2_-2/C-3, H_3_-18/C-3 (Figure 2). Meanwhile, the position of the trisubstituted olefin was determined at ∆^7,8^ by the HMBC correlations between H_3_-19/C-7, H-7/C-5, and H_3_-18/C-5, as well as the ^1^H–^1^H COSY correlations between H_3_-18/H-4, H-4/H-5, H-5/H-6, and H-6/H-7. The *trans*-olefinic geometry of ∆^7,8^ was determined by the NOESY correlations between H-7/H-10*α,* and the absence of H-7/H_3_-19 (Figure 3). Furthermore, OH-11 and CH_3_-18 were both *β*-orientated, which were deduced by the NOESY correlations between H_3_-20/H-13*β*, H-11/H-13*α*, and H_3_-17/H_3_-18.

**FIGURE 6:**
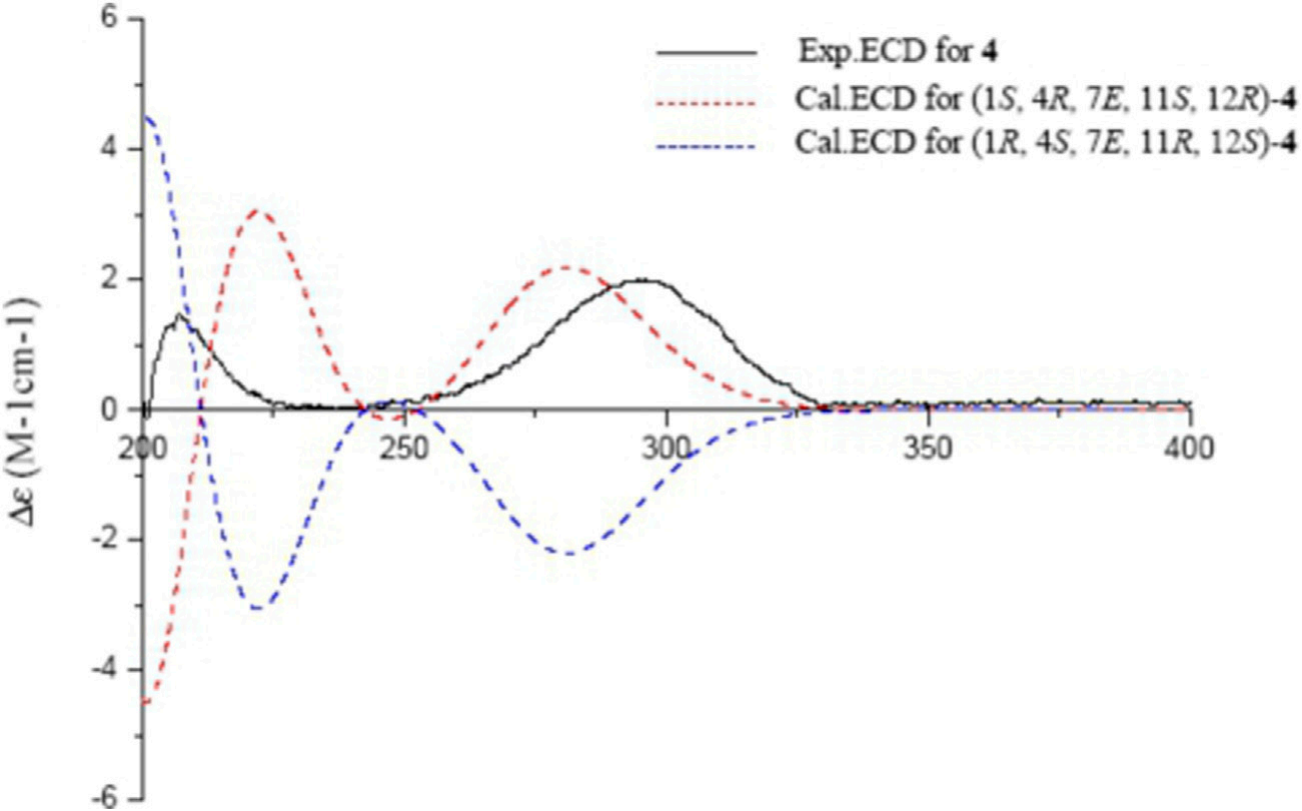
Experimental and calculated ECD spectra of **4** and its enantiomer (red and blue calculated at the B3LYP/6–311++G (2d,*p*)//B3LYP/6–31+G (d,*p*) level in CH_3_OH; black, experimental in CH_3_OH).

The absolute configurations of **4** were determined by comparison of experimental and calculated ECD spectra. Time dependent density functional theory (TDDFT) at the B3LYP/6-311++G (2d,*p*) level with IEFPCM in MeOH was used to calculate the ECD spectra of two enantiomers of **4**. The experimental ECD spectrum of **4** showed a positive cotton effect (CE) at 292 nm (n → π*), a negative CE at 239 nm (π → π*), and a positive CE at 205 nm (n → σ*), which coincided well with the calculated ECD spectrum of (1*S*,4*R*,7*E*,11*S*,12*R*)-**4** (Figure 6), Thus, the structure of sacraoxide C (**4)** was determined as (1*S*,4*R*,11*S*,12*R*)-1:12-epoxy-11-hydroxy-cembra-7*E*-en-3-one.

Sacraoxide D (**5**) is an acetylated derivate of compound **4**, which was indicated by both HRESIMS and NMR spectroscopic data. The acetoxy group was determined at C-11, since the proton resonance of H-11 (*δ*_H_ 5.13) was observed downfield shifted (Table 1), and the HMBC correlation between H-11/C-1′ was also observed. The ECD spectra of **5** and **4** revealed high similarities, suggesting the same absolute configurations. Thus, the structure of sacraoxide D (**5**) was determined as (1*S*,4*R*,11*S*,12*R*)-1:12-epoxy-11-acetoxy-cembra-7*E*-en-3-one.

**FIGURE 7:**
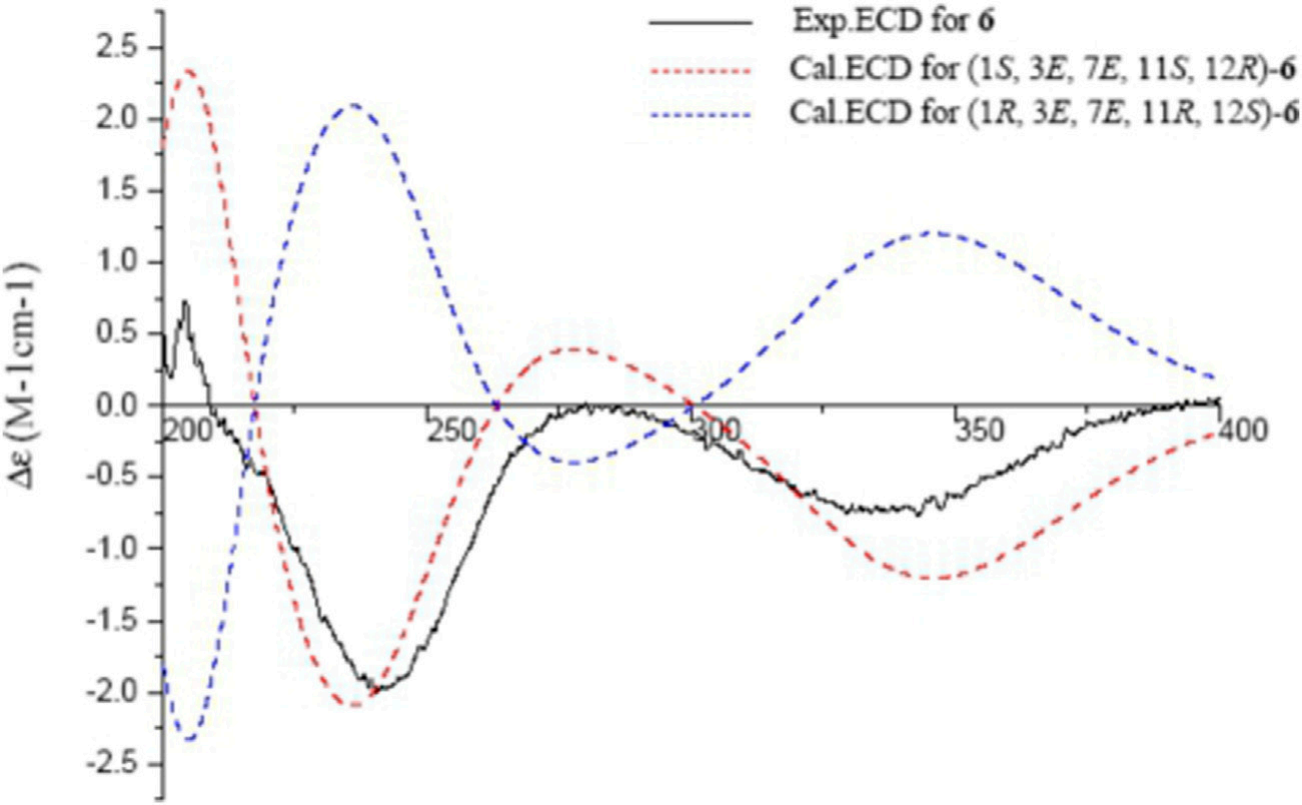
Experimental and calculated ECD spectra of **6** and its enantiomer (red and blue calculated at the B3LYP/6–311++G (2d,p)//B3LYP/6–31+G (d,*p*) level in CH_3_OH; black, experimental in CH_3_OH).

Sacraoxide E (**6**) was isolated as colorless oil [*α*]^25^_D_ −64.0 (*c* 0.10, MeOH). The molecular formula of **6** was established as C_22_H_34_O_4_ from the HRESIMS positive-ion at *m/z* 385.2354 [M + Na]^+^ (calculated for C_22_H_34_O_4_Na, 385.2355). Compound **6** is also a cembranoid, with a tetrahydrofuran ring and 11*β*-acetoxy group, which was suggested by its superimposable resonances in comparison with structurally similar compounds **9**, **3**, and **5**. Other functionalities were suggested to be two trisubstituted olefin groups and a carbonyl moiety. One olefin was assigned to be a *trans*-∆^3,4^ moiety by the HMBC correlations between H-3/C-1, H-3/C-18, and H_3_-18/C-3, as well as the NOESY correlations between H-3/H-5*α*, and H-2*β*/H-18 (Figures 2, 3). In addition, the presence of an *α,β*-unsaturated ketone moiety was suggested by observation of a downfield olefinic proton at *δ*_H_ 6.53 (H-7) and a carbonyl carbon resonance at *δ*_C_ 201.2 (C-6), which were assigned as 7,8-en-6-one by the ^3^ *J*-HMBC correlations between H_3_-19/C-7, H-7/C-5, and H_3_-18/C-5, as well as the ^2^ *J*-HMBC correlations between H-5/C-6, and H-7/C-6. The *trans*-olefinic geometry of ∆^7,8^ was evident by the NOESY correlation between H-10*α*/H-7. The experimental ECD spectrum of **6** showed a negative CE at 348 nm (n → π*), a positive CE at 280 nm (π → π*), a negative CE at 241 nm (π → π*), and a positive CE at 203 nm (n → σ*), which coincided well with the calculated ECD spectrum of (1*S*,3*E*,7*E*,11*S*,12*R*)-**6** (Figure 7). Thus, the structure of sacraoxide E (**6**) was determined as (1*S*,11*S*,12*R*)-1:12-epoxy-11-acetoxy-cembra-3*E*,7*E*-dien-6-one.

**FIGURE 8:**
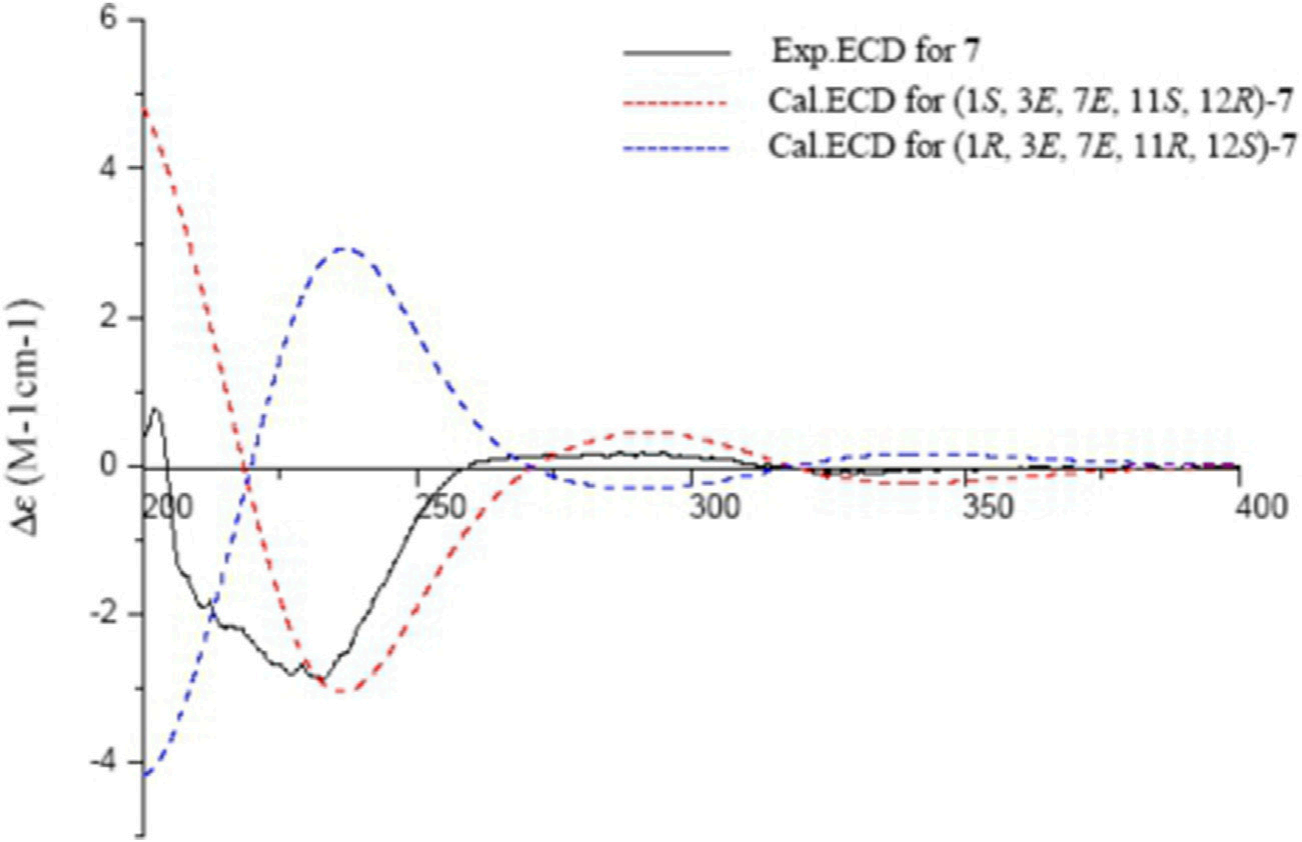
Experimental and calculated ECD spectra of **7** and its enantiomer (red and blue calculated at the B3LYP/6–311++G (2d,p)//B3LYP/6–31+G (d,*p*) level in CH_3_OH; black, experimental in CH_3_OH).

Sacraoxide F (**7**) has the same molecular formula of C_22_H_34_O_4_ as **6**, which was established from a protonated molecule at *m/z* 363.2504 [M + H]^+^ (calculated for C_22_H_35_O_4_, 363.2535) in the HRESIMS spectrum. The functionalities of the cembranoid skeleton of compound **7** included a tetrahydrofuran ring and 11*β*-acetoxy group, as well as two trisubstituted olefin groups and a carbonyl moiety. Detailed analyses of the 2D NMR spectroscopic data assigned the *α,β*-unsaturated ketone moiety as 7,8-en-9-one by the HMBC correlations between H_3_-19/C-7, H_3_-19/C-9, and H-11/C-9 (Figure 2). Additionally, the experimental ECD spectrum of **7** coincided well with calculated ECD spectrum of (1*S*,3*E*,7*E*,11*S*,12*R*)-**7** (Figure 8). Thus, the structure of sacraoxide F (**7**) was determined as (1*S*,11*S*,12*R*)-1:12-epoxy-11*β*-acetoxy-cembra-3*E*,7*E*-dien-9-one.

**FIGURE 9:**
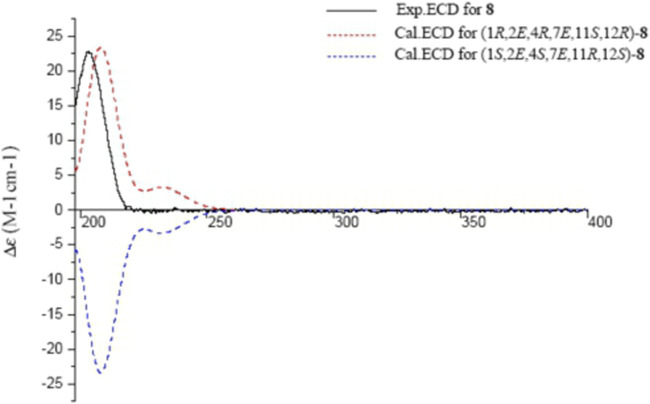
Experimental and calculated ECD spectra of **8** and its enantiomer (red and blue calculated at the B3LYP/6–311++G (2d,p)//B3LYP/6–31+G (d,*p*) level in CH_3_OH; black, experimental in CH_3_OH).

Sacraoxide G (**8**) was isolated as white amorphous powder [*α*]^25^_D_ +115.0 (*c* 0.11, MeOH). The molecular formula of **8** was established as C_20_H_34_O_3_ from the HRESIMS positive-ion at *m/z* 305.2583 [M − H_2_O + H]^+^ (calculated for C_20_H_33_O_2_, 305.2475). Compound **8** is a cembranoid with a tetrahydrofuran ring and OH-11*β* group, which was suggested by its superimposable resonances in comparison with structurally similar compounds **1**, **2** and **4**. In comparison to compound **4**, the different functionalities of **8** were suggested to be a disubstituted *trans*-olefin [*δ*_H_ 5.54 (d, *J* = 15.4 Hz, H-2), 5.81 (d, *J* = 15.4 Hz, H-3)], and an oxygenated quaternary carbon [*δ*_C_ 74.4 (C-4)] (Tables 2, 3). Their positions were assigned by the HMBC correlations between H-3/C-1, H-2/C-15, H_3_-18/C-3, and H-2/C-4 (Figure 2). The *β*-orientation of OH-4 and OH-11 were evident by the NOESY correlations between H_3_-20/H-13*β,* H-13*α*/H-11 and H_3_-18/H-11 (Figure 3). The experimental ECD spectrum of **8** coincided well with the calculated ECD spectrum of (1*R*,2*E*,4*R*,7*E*,11*S*,12*R*)-**8** (Figure 9). Thus, the structure of sacraoxide G (**8**) was determined as (1*R*,4*R*,11*S*,12*R*)-1:12-epoxy-cembra-2*E*,7*E*-dien-4,11-diol.

Nitric oxide (NO) plays significant roles in immune and inflammatory responses. The inhibition of NO release may be considered therapeutic in the treatment of inflammatory diseases (Strowig et al., 2012). Taking account of the traditional usage of the olibanum, cembranoids (**1**–**9**) were evaluated for their inhibitory activities against lipopolysaccharide (LPS)-induced NO production in RAW 264.7 mouse monocyte-macrophages. Compounds **6** and **7** showed the most potent inhibitory activities with the IC_50_ values of 36.4 and 24.9 *μ*M respectively, while the others were less active or inactive (Table 4). In addition, the MTT assay indicated that none of these compounds showed cytotoxicity in RAW264.7 cells at a concentration of 50 *μ*M. The presence of an *α,β*-unsaturated carbonyl functionality seems essential for the inhibitory activity.

**TABLE 4:** Inhibitory activity of compounds **1**–**9** against NO production in RAW 264.7 cells.

Compound^a^	IC_50_ (µM)
**4**	72.1 ± 5.1
**6**	36.4 ± 2.9
**7**	24.9 ± 1.7
HSS^b^	52.2 ± 4.5
Indomethacin^b^	11.9 ± 0.6

^a^ Compounds **1**–**3**, **5**, and **8**–**9** showed activities with IC_50_ > 100 *µ*M.

^b^ HSS, (hydrocortisone sodium succinate) and indomethacin were used as a positive control. Data are presented based on three experiments.

## Conclusion

A phytochemical investigation on the gum resin of *B. sacra* resulted in the isolation and structural elucidation of seven undescribed and two known cembranoids. These cembranoids possess a common tertrahydrofuran ring structure through an ether bond between C-1 and C-12. The structures including absolute configurations were determined by extensive physicochemical and spectroscopic analysis, as well as ECD calculation, modified Mosher's method and X-ray diffraction crystallography. Compounds **4**, **6**, and **7** displayed inhibitory activities against LPS-induced NO production in RAW 264.7 cells with IC_50_ values ranging from 24.9 to 72.1 *μ*M. These findings will be of particular value for further studies of structurally interesting cembranoids with biological activities from the genus of *Boswellia*.

## Data Availability

The datasets presented in this study can be found in online repositories. The names of the repository/repositories and accession number(s) can be found in the article/Supplementary Material.
