# Supplementary material for: Sacraoxides A–G, Bioactive Cembranoids from Gum Resin of Boswellia sacra
Source: Front Chem. 2021 Mar 30;9:649287. doi: 10.3389/fchem.2021.649287 (PMC8044883; doi:10.3389/fchem.2021.649287)
Supplement: Supplementary file 1 [file datasheet1.pdf]

Supplementary Material

Contents

1. Spectroscopic data P2-44

**Figure 1-1~1-11.** 1D/2D NMR, HRESIMS, IR, UV, and ECD Spectra of Compound **1**	P2
**Figure 2-1~2-4.** 1D/2D NMR, and ECD Spectra of Compound **2**	P7
**Figure 3-1~3-5.** 1D/2D NMR, and HRESIMS Spectra of Compound **2a**	P9
**Figure 4-1~4-5.** 1D/2D NMR, and HRESIMS Spectra of Compound **2b**	p12
**Figure 5-1~5-10.** 1D/2D NMR, HRESIMS, IR, UV, and ECD Spectra of Compound **3**	p14
**Figure 6-1~6-10.** 1D/2D NMR, HRESIMS, IR, UV, and ECD Spectra of Compound **4**	P19
**Figure 7-1~7-10.** 1D/2D NMR, HRESIMS, IR, UV, and ECD Spectra of Compound **5**	p24
**Figure 8-1~8-10.** 1D/2D NMR, HRESIMS, IR, UV, and ECD Spectra of Compound **6**	p29
**Figure 9-1~9-10.** 1D/2D NMR, HRESIMS, IR, UV, and ECD Spectra of Compound **7**	p34
**Figure 10-1~10-11.** 1D/2D NMR, HRESIMS, IR, UV, and ECD Spectra of Compound **8**	p39

2. X-ray crystallographic data for 1 .... p44-45

3. ECD calculations for 4, 6−8 .... p45-48

1. Spectroscopic data

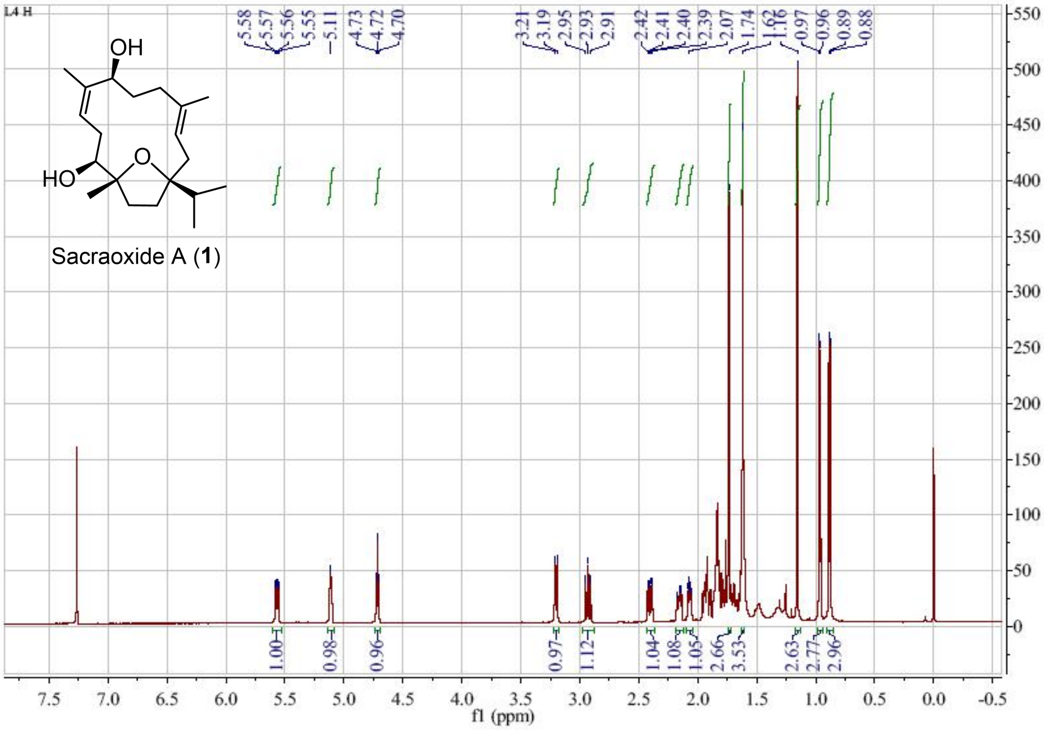

Supplementary Figure 1-1. ^{1}H NMR Spectrum of compound **1**

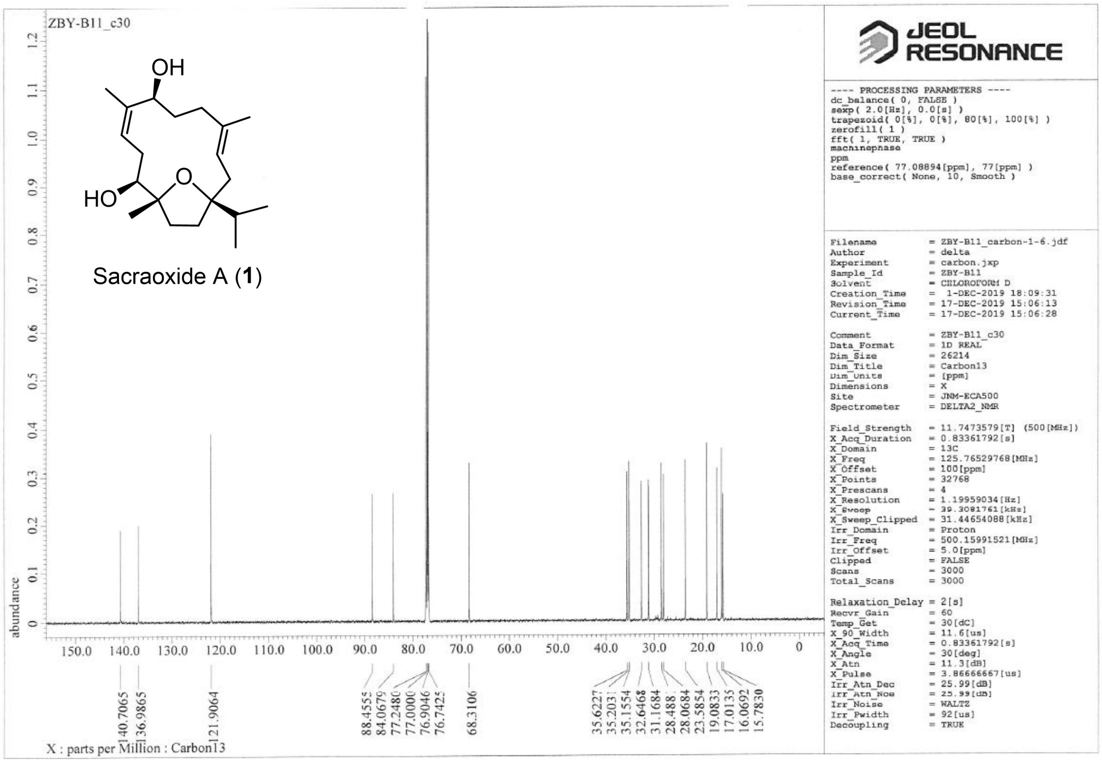

Supplementary Figure 1-2. ^{13}C NMR Spectrum of compound **1**

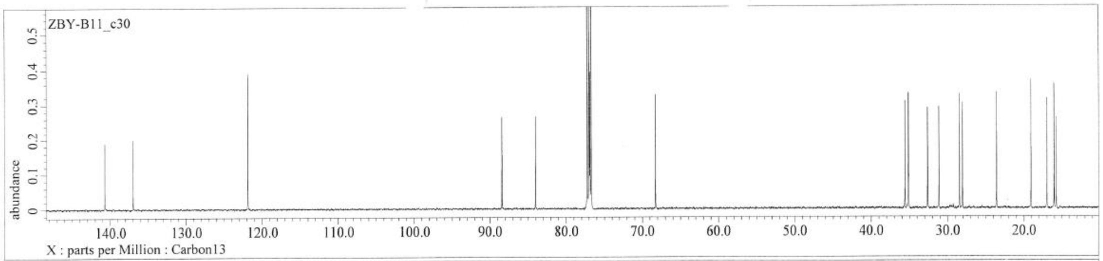

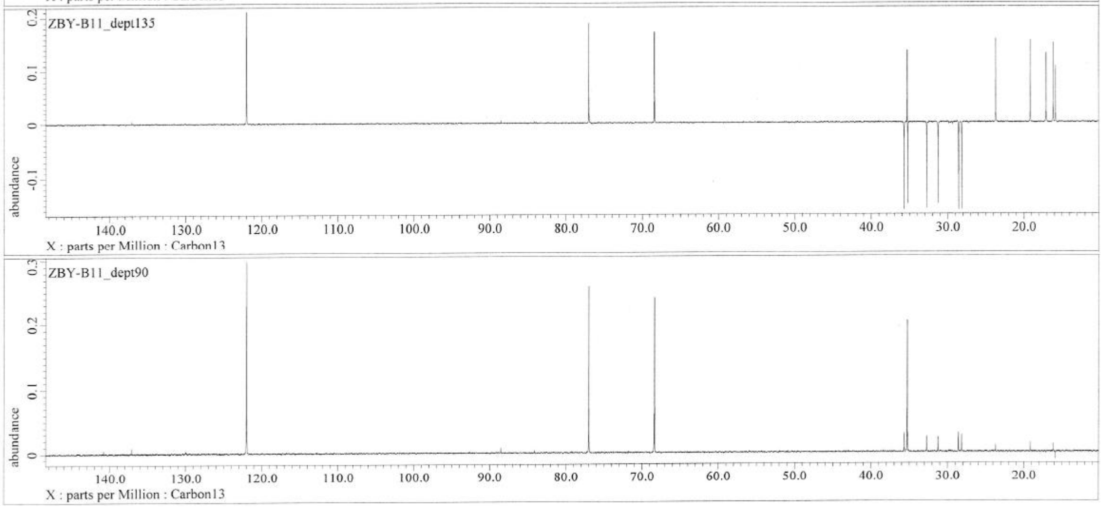

Supplementary Figure 1-3. DEFT Spectrum of compound **1**

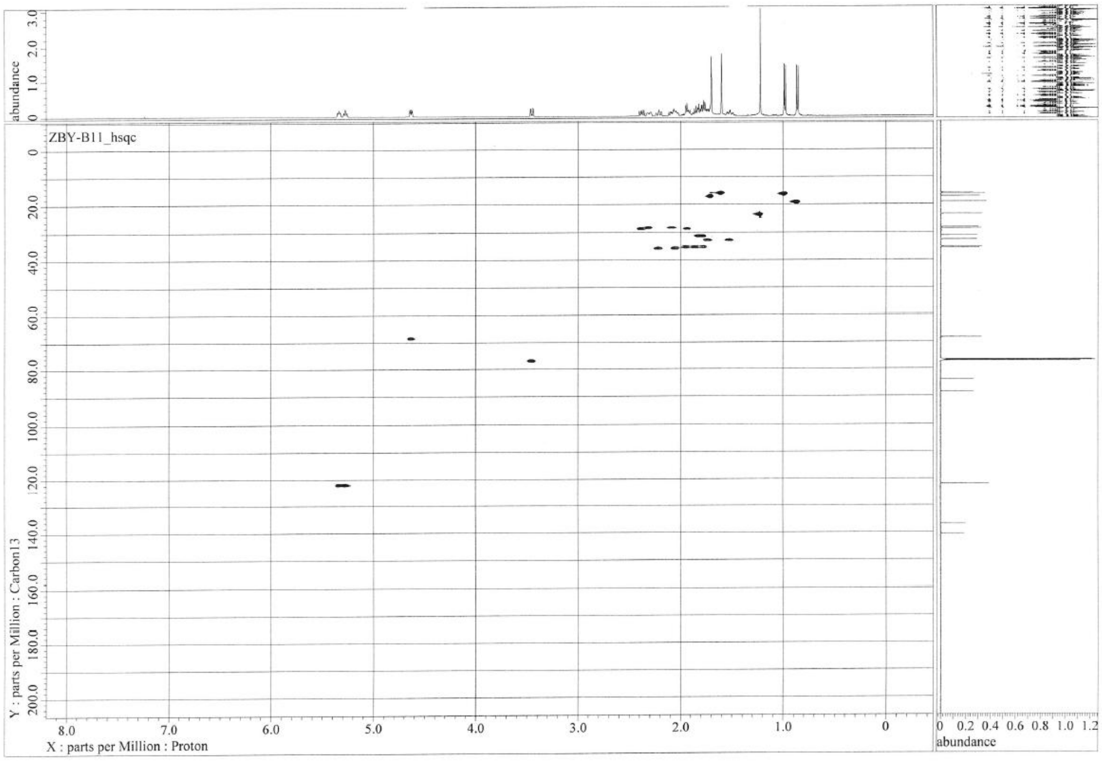

Supplementary Figure 1-4. HSQC Spectrum of compound **1**

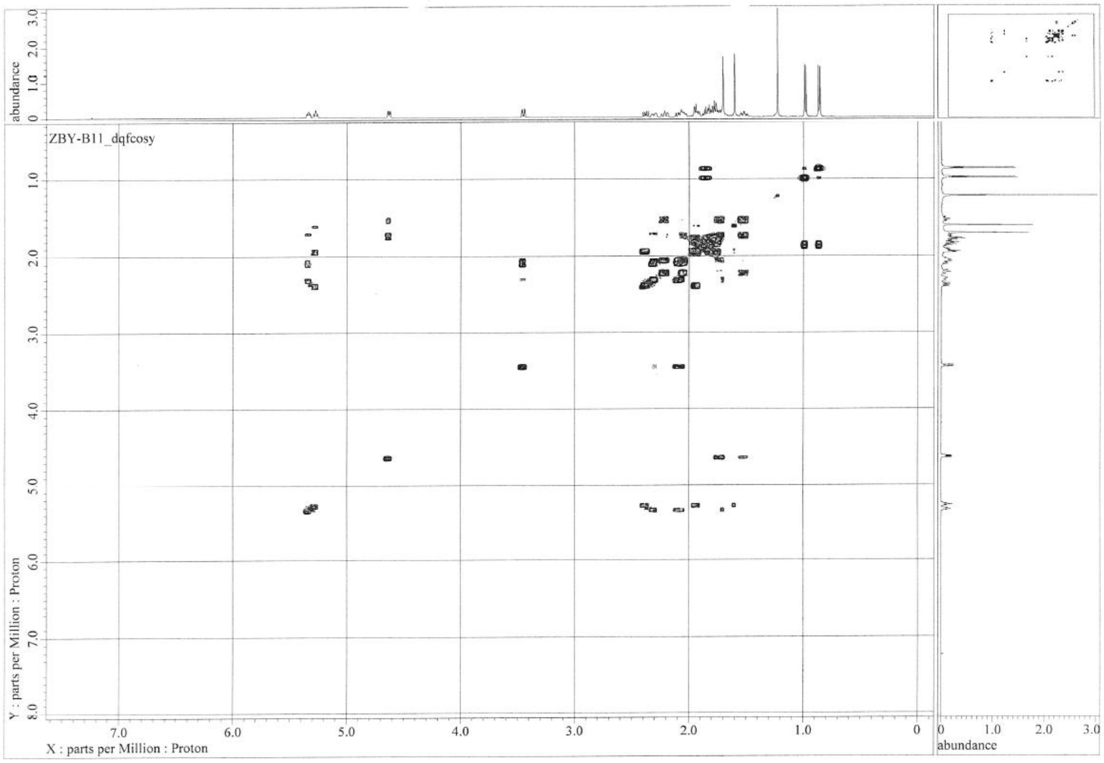

Supplementary Figure 1-5. ^{1}H-^{1}H COSY Spectrum of compound **1**

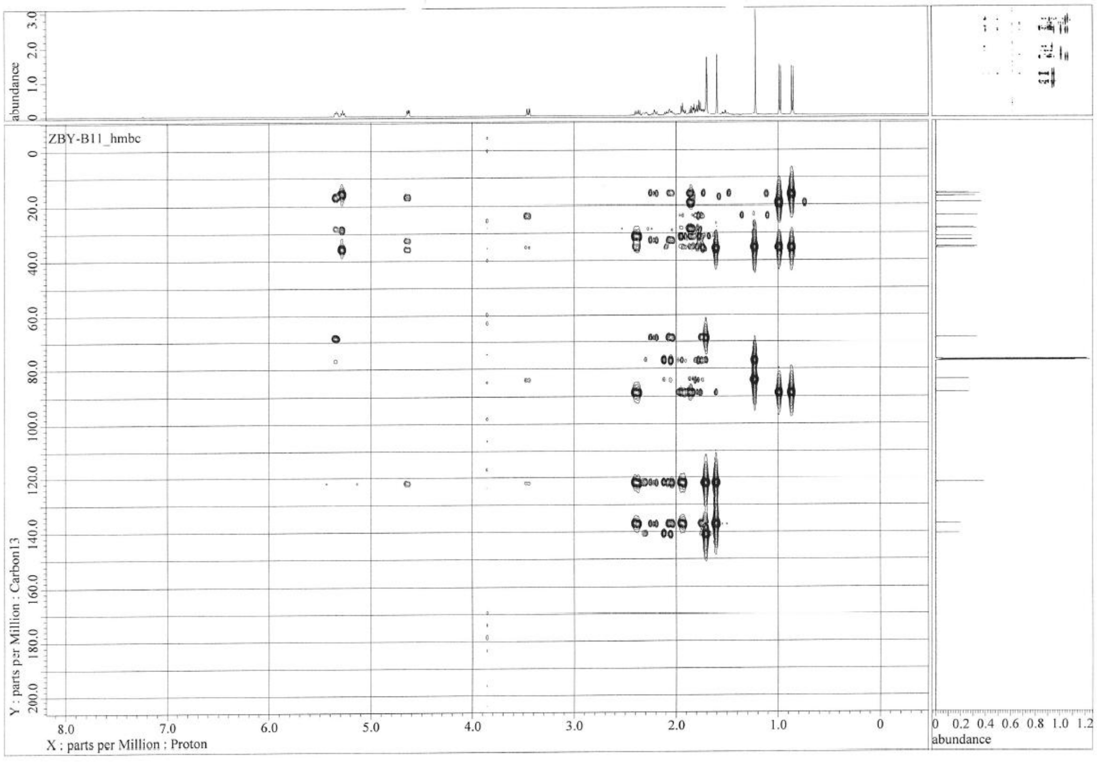

Supplementary Figure 1-6. HMBC Spectrum of compound **1**

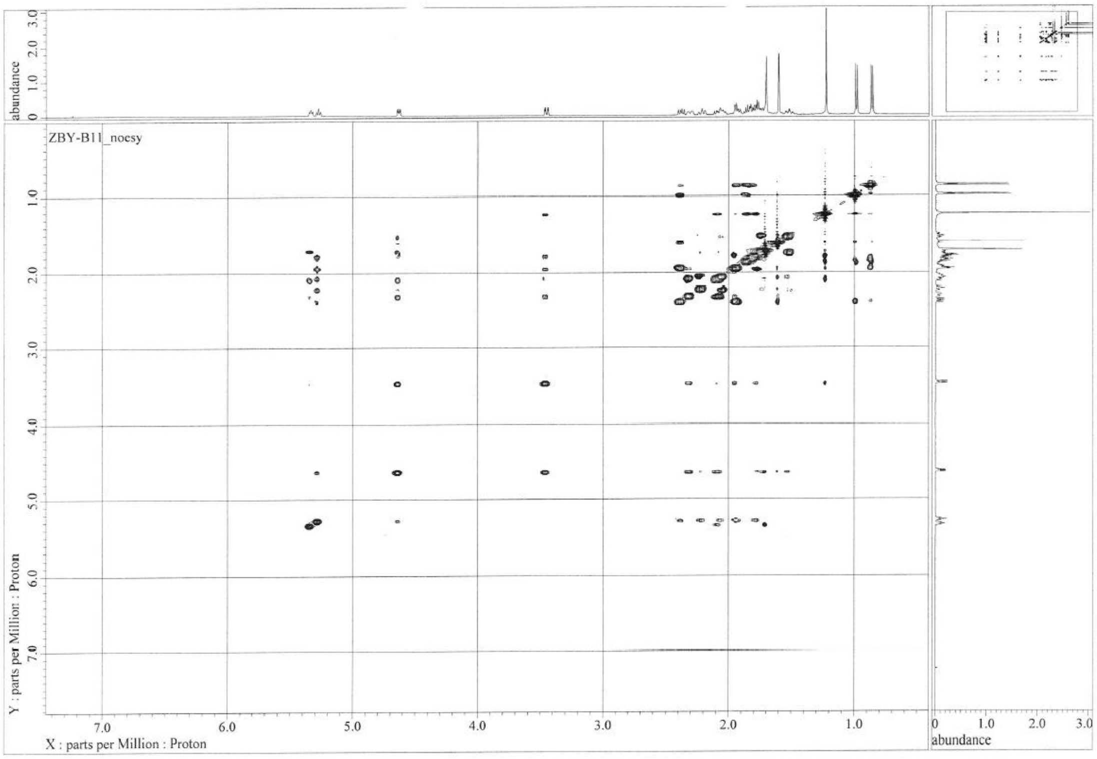

Supplementary Figure 1-7. NOESY Spectrum of compound **1**

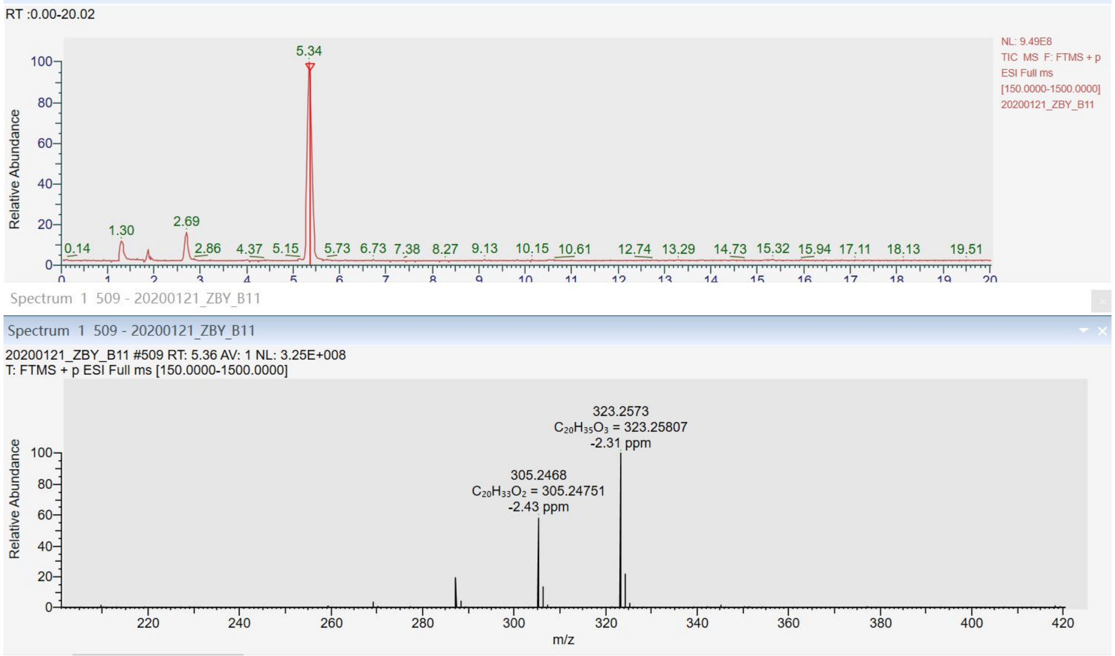

Supplementary Figure 1-8. HRESIMS data of compound **1**

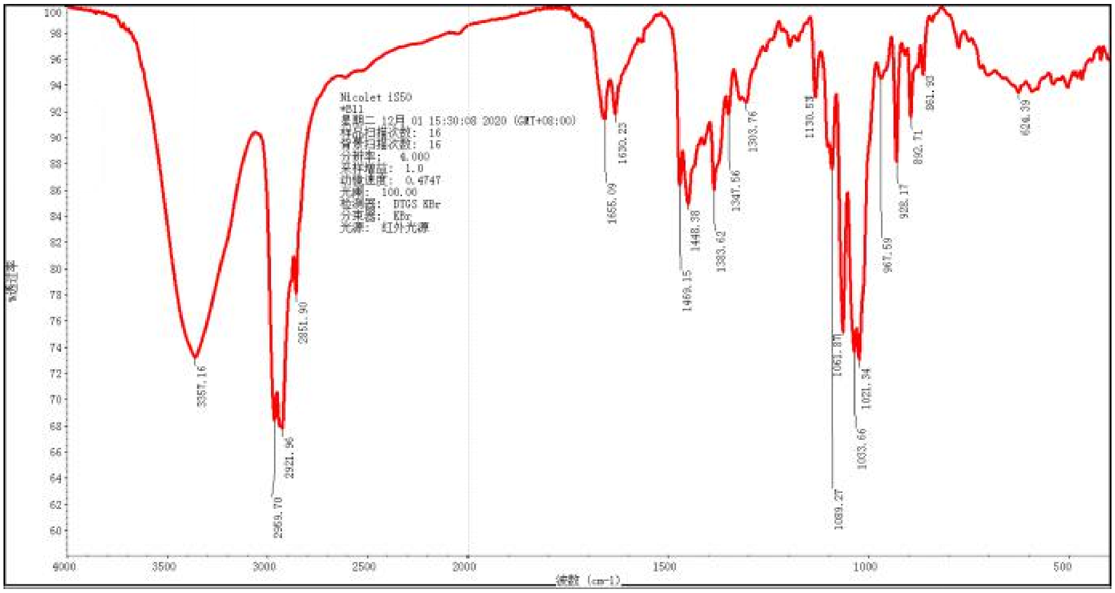

Supplementary Figure 1-9. IR Spectrum of compound **1**

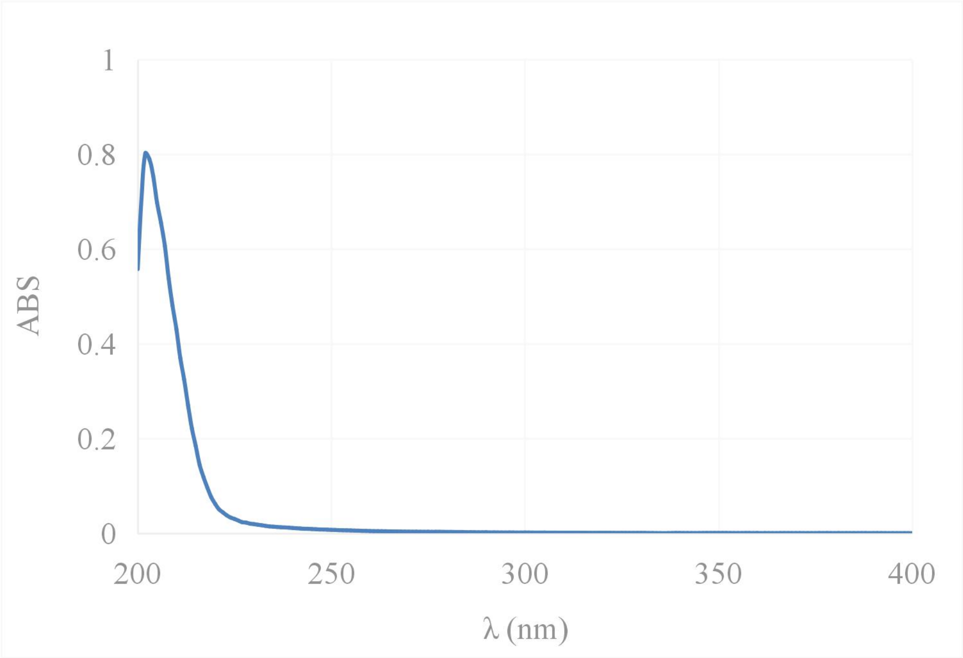

Supplementary Figure 1-10. UV Spectrum of compound **1**

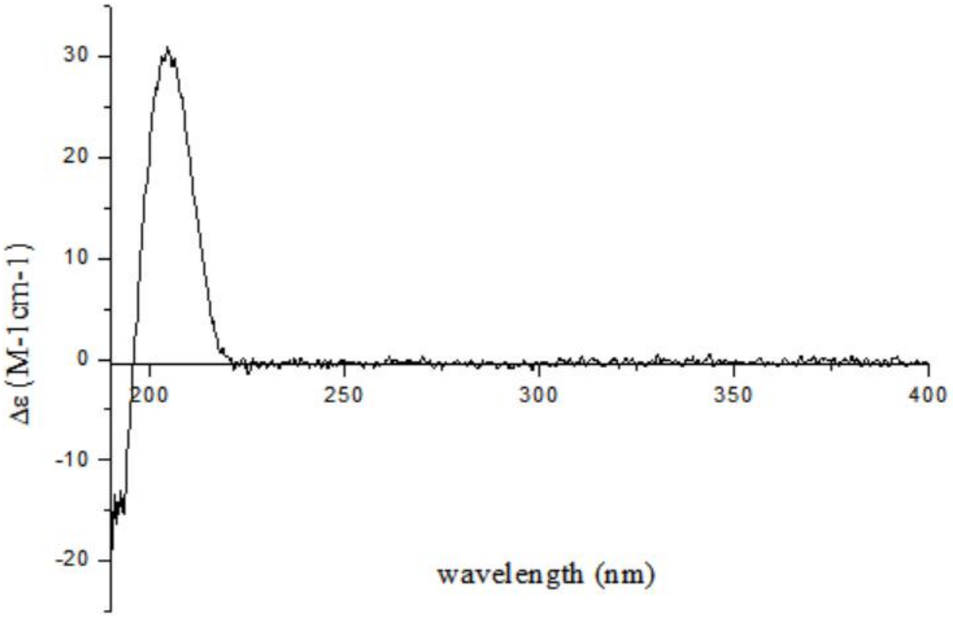

Supplementary Figure 1-11. ECD Spectrum of compound **1**

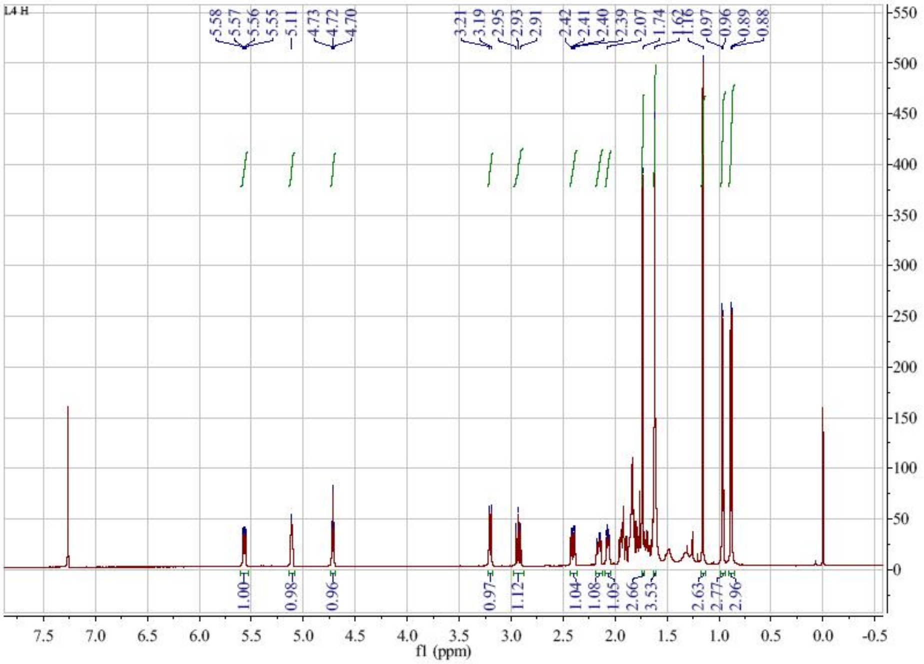

Supplementary Figure 2-1. ^{1}H NMR Spectrum of compound **2**

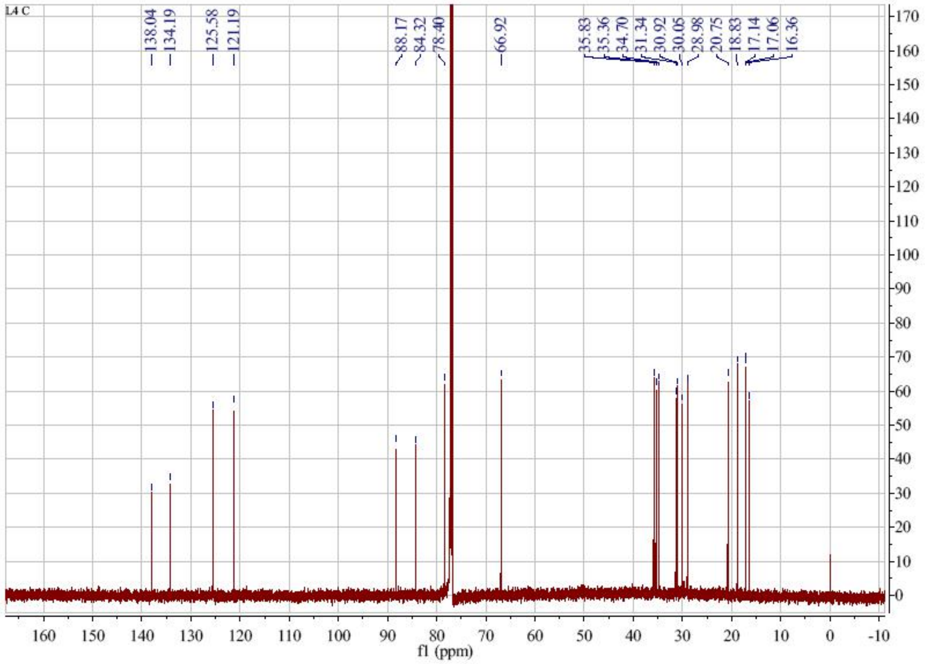

Supplementary Figure 2-2. ^{13}C NMR Spectrum of compound **2**

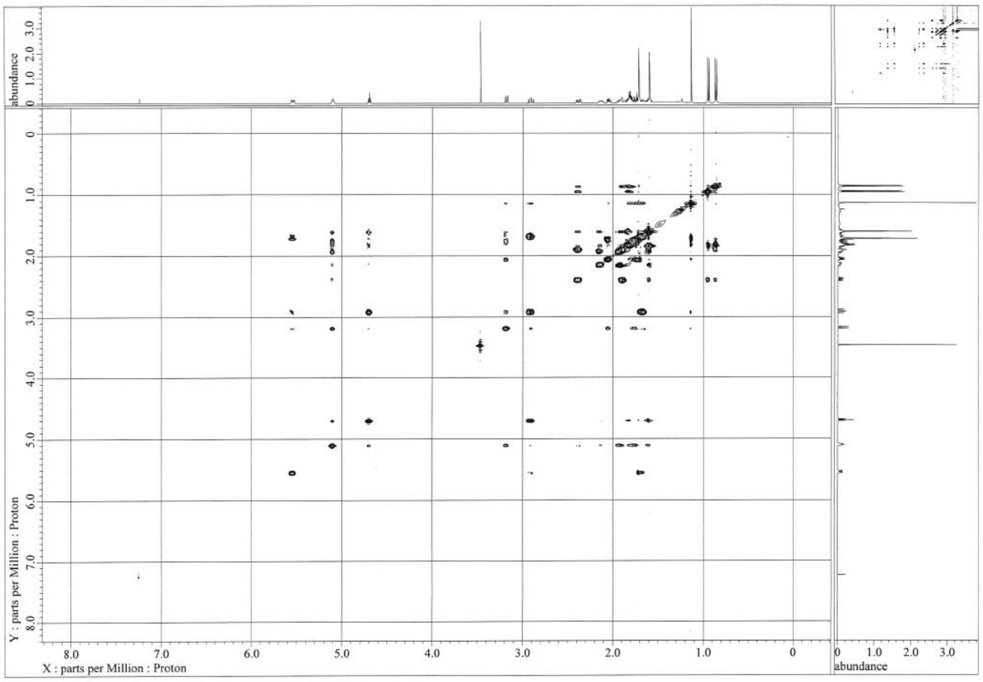

Supplementary Figure 2-3. NOESY Spectrum of compound **2**

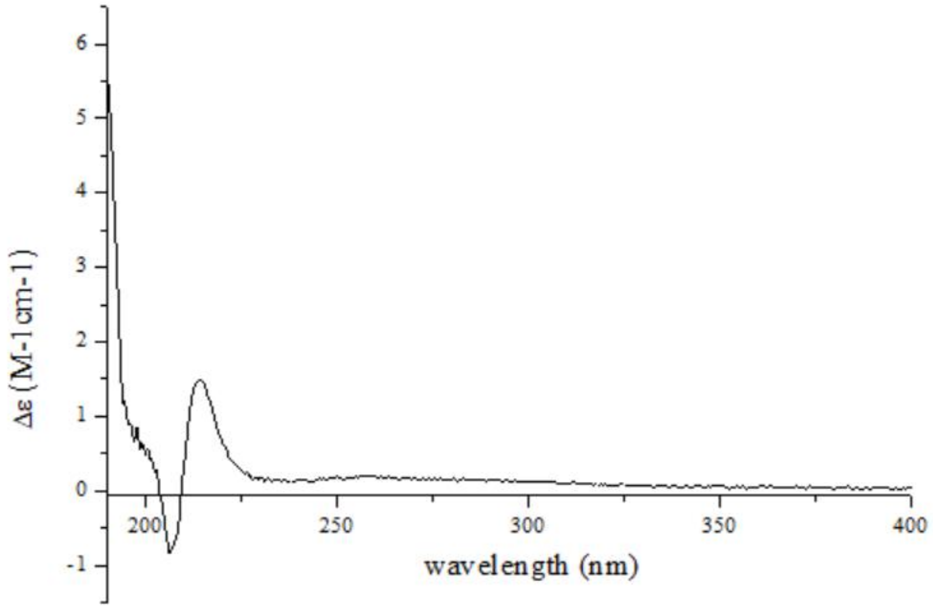

Supplementary Figure 2-4. ECD Spectrum of compound **2**

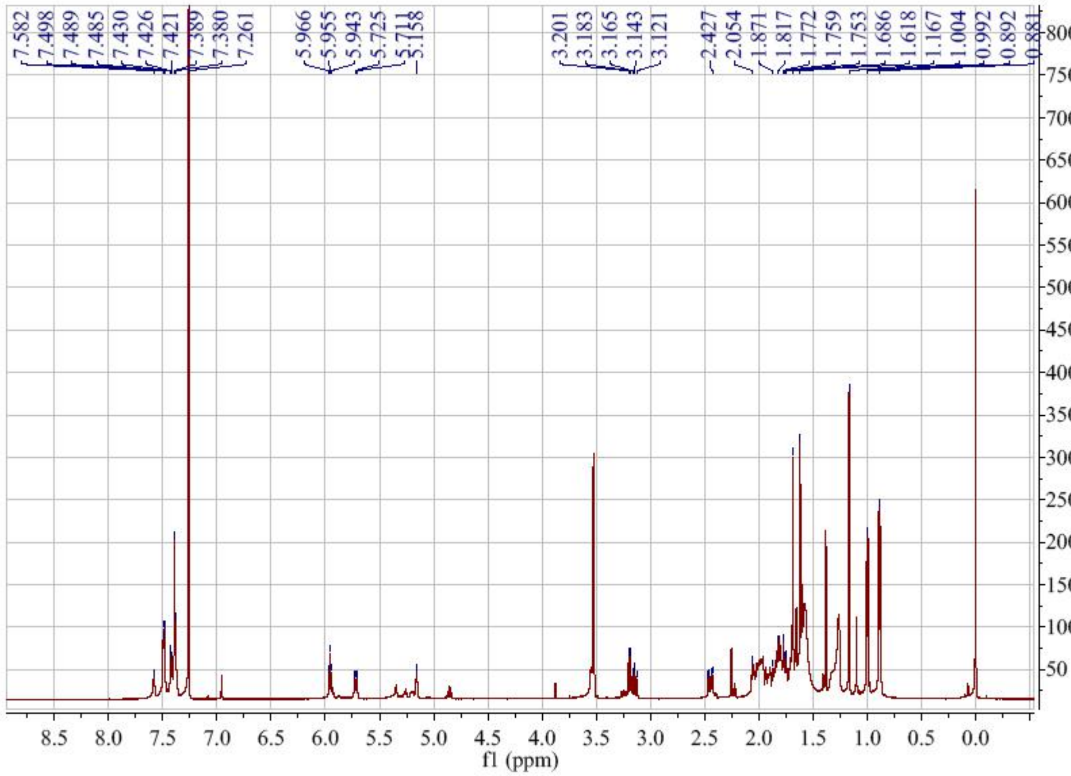

Supplementary Figure 3-1. ^{1}H NMR Spectrum of compound **2a**

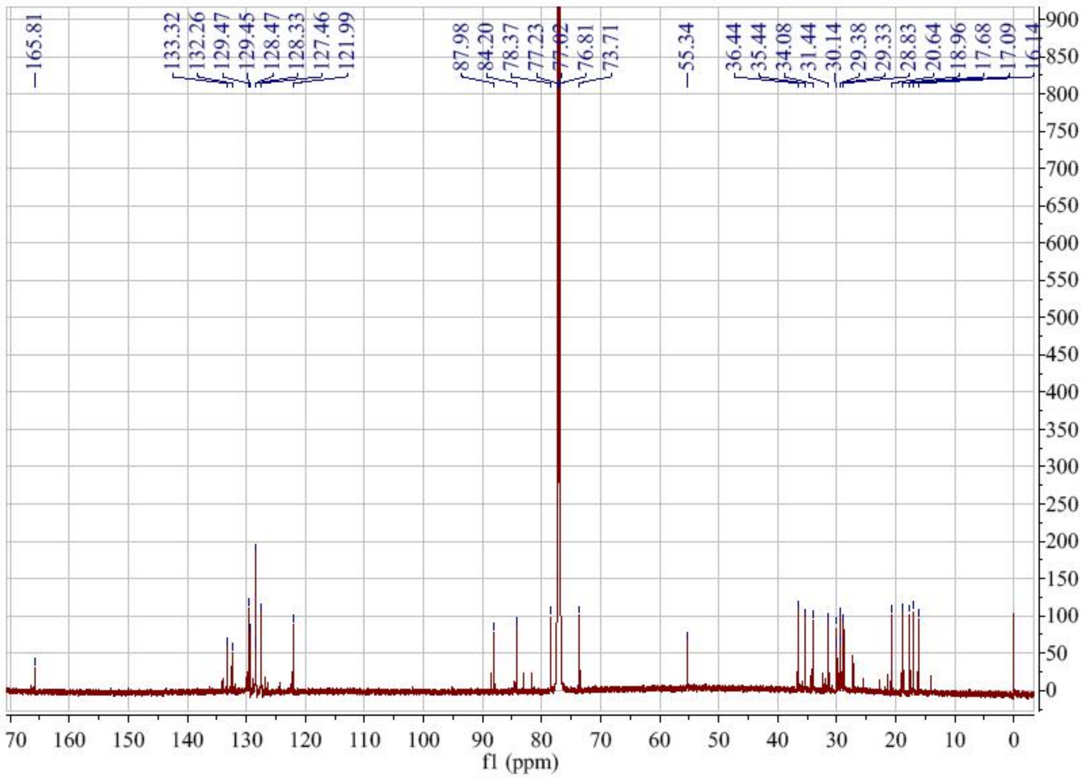

Supplementary Figure 3-2. ^{13}C NMR Spectrum of compound **2a**

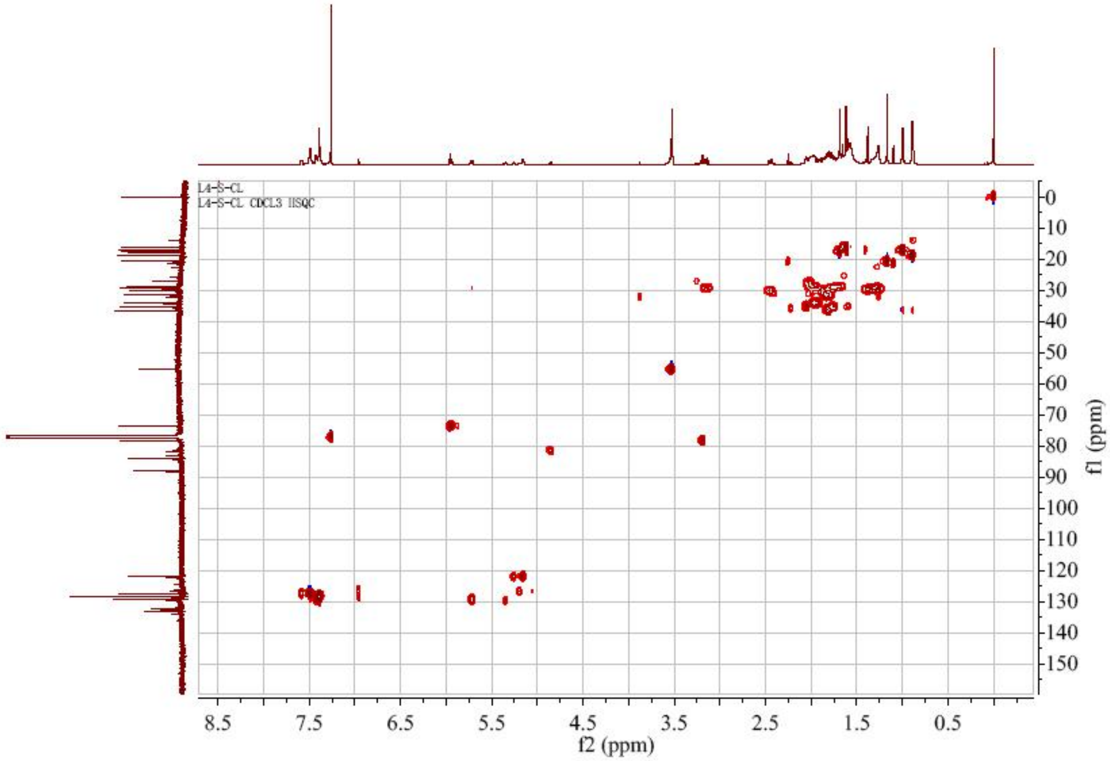

Supplementary Figure 3-3. HSQC Spectrum of compound 2a

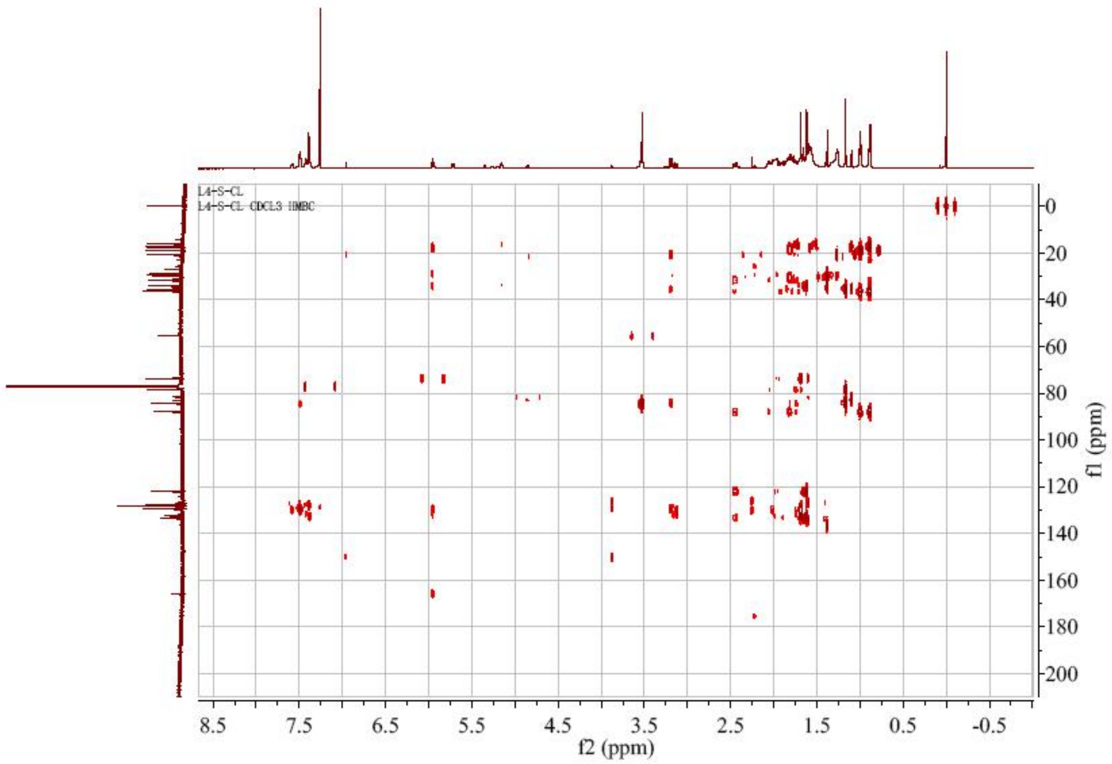

Supplementary Figure 3-4. HMBC Spectrum of compound **2a**

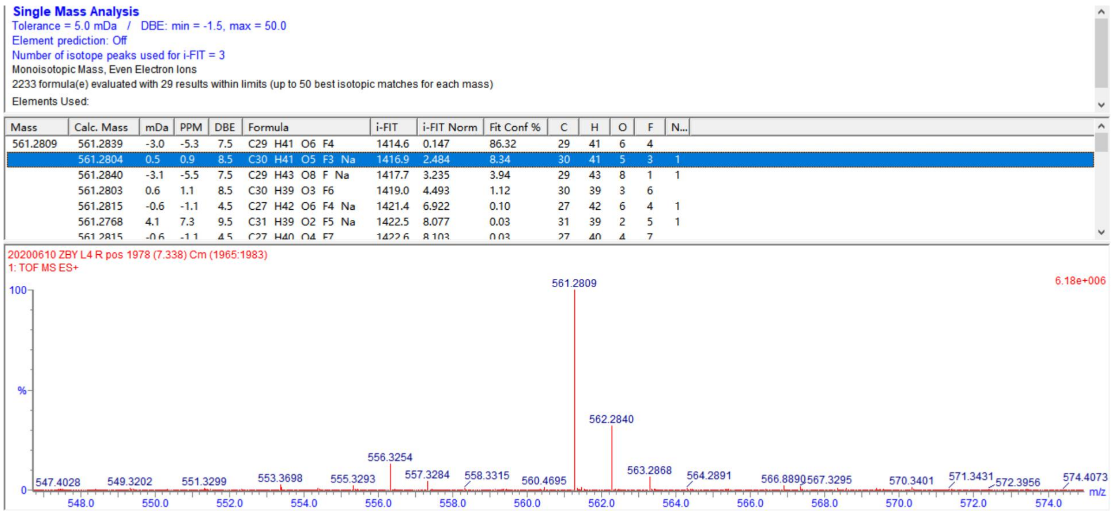

Supplementary Figure 3-5. HRESIMS data of compound **2a**

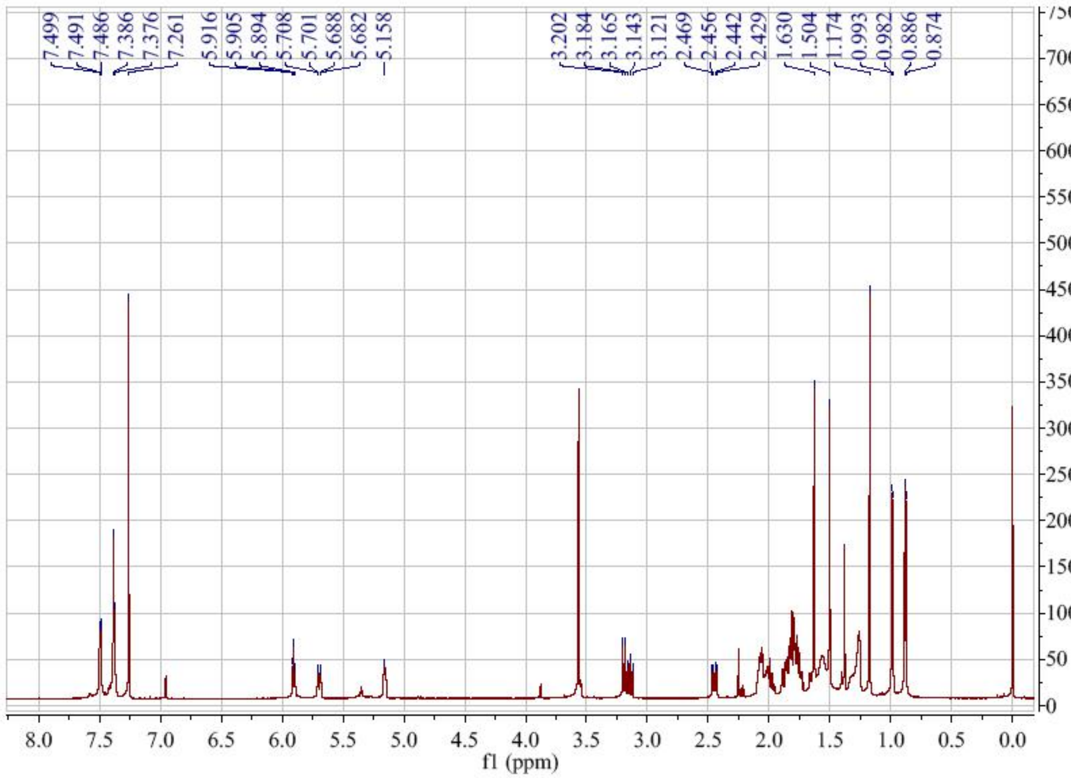

Supplementary Figure 4-1. 1H NMR Spectrum of compound **2b**

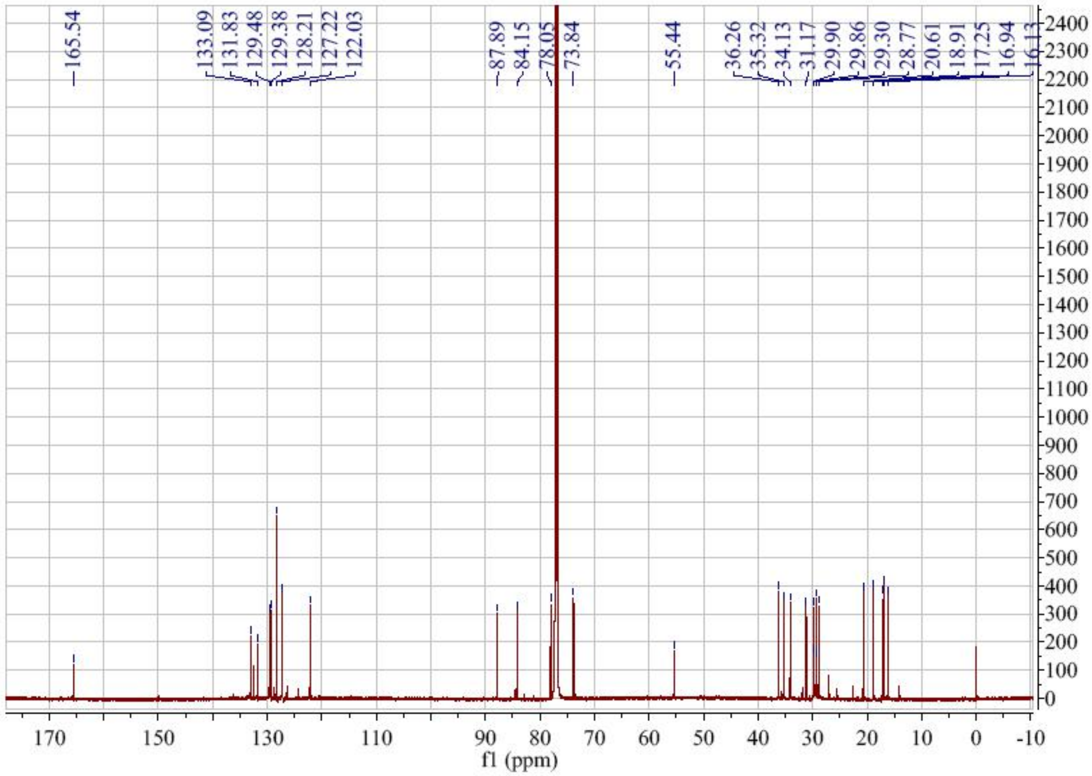

Supplementary Figure 4-2. ^{13}C NMR Spectrum of compound **2b**

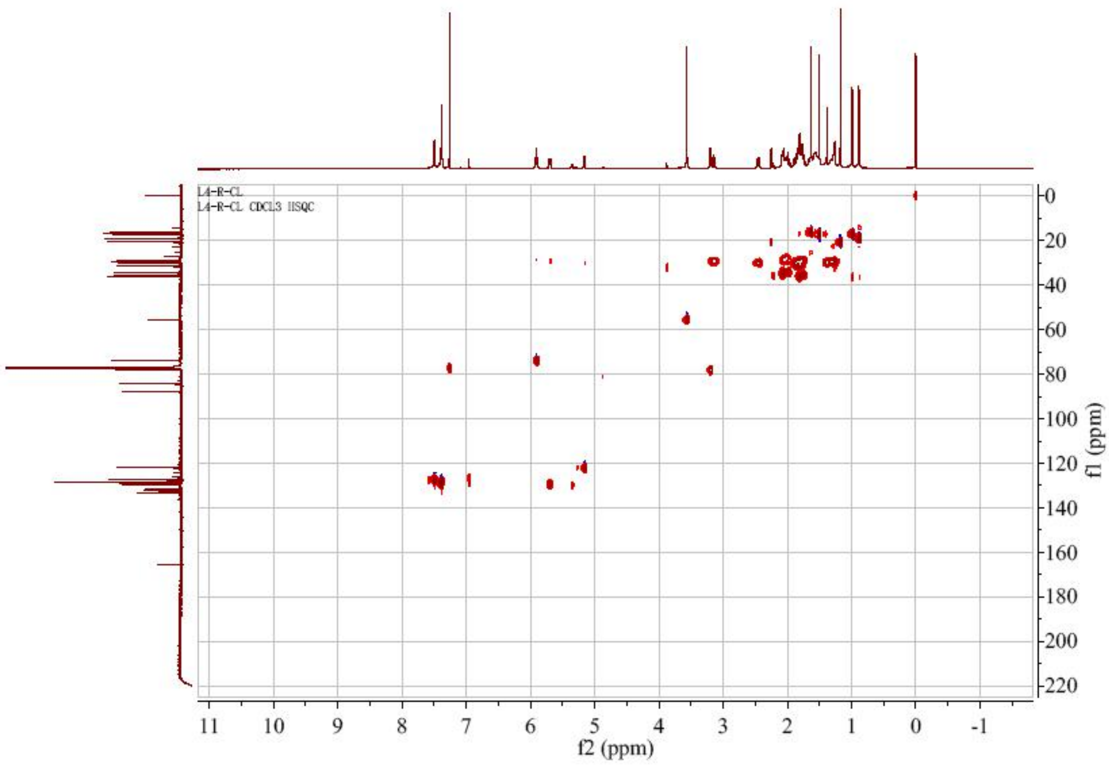

Supplementary Figure 4-3. HSQC Spectrum of compound **2b**

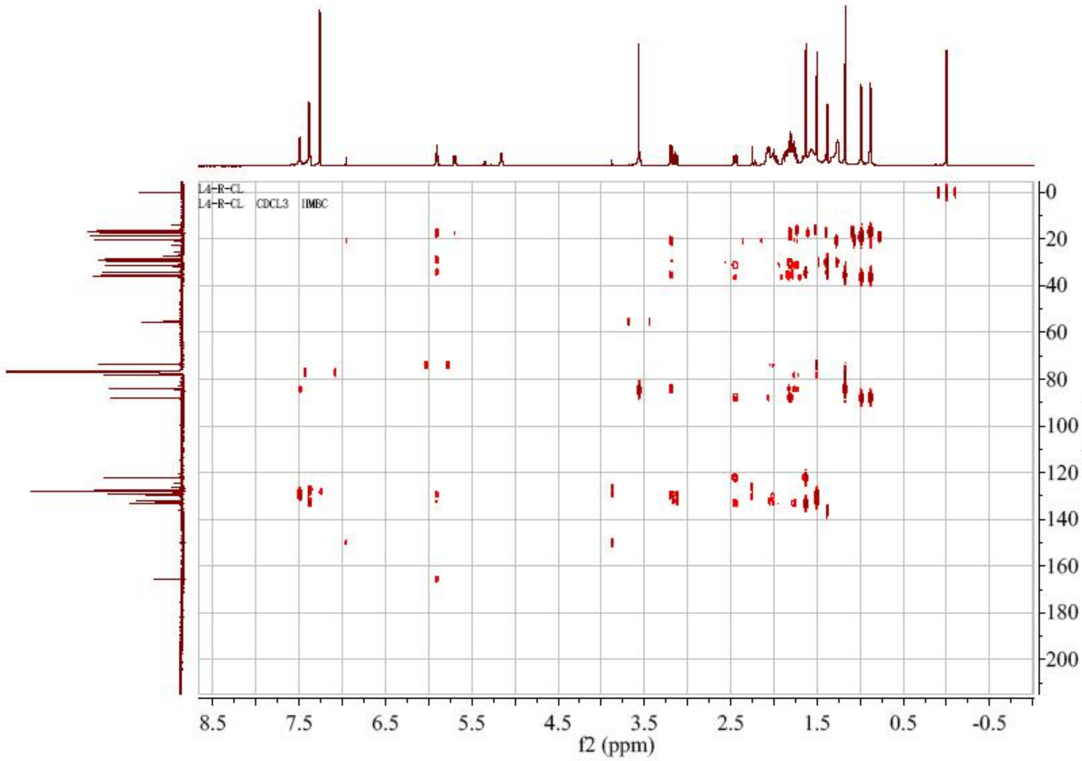

Supplementary Figure 4-4. HMBC Spectrum of compound **2b**

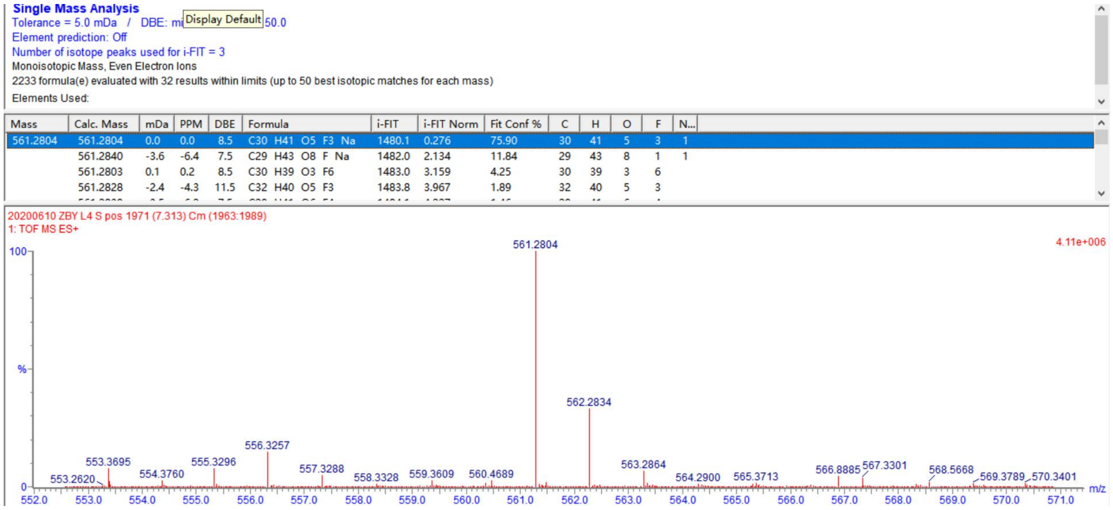

Supplementary Figure 4-5. HRESIMS data of compound **2b**

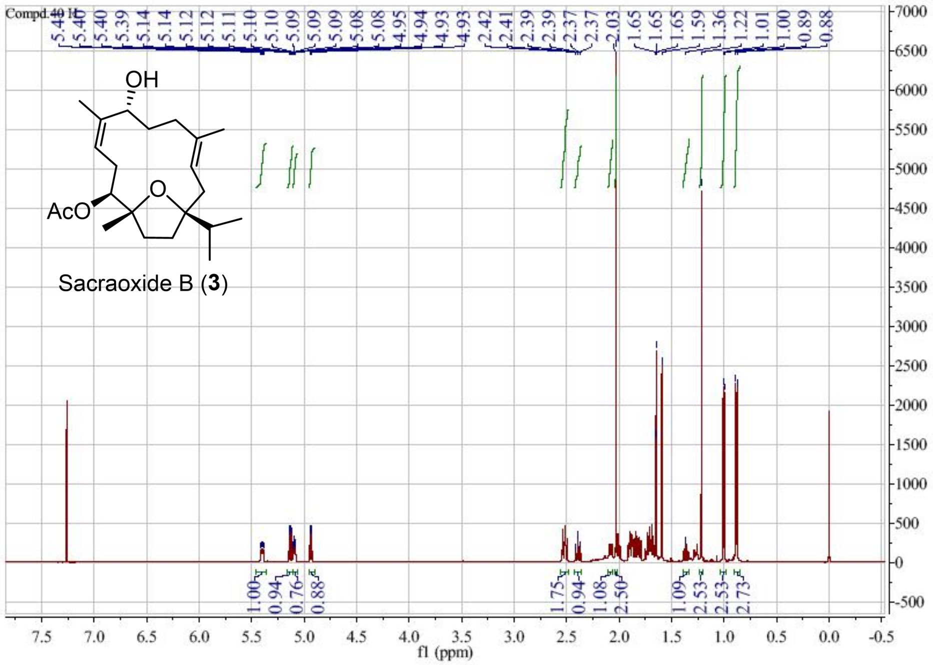

Supplementary Figure 5-1. ^{1}H NMR Spectrum of compound **3**

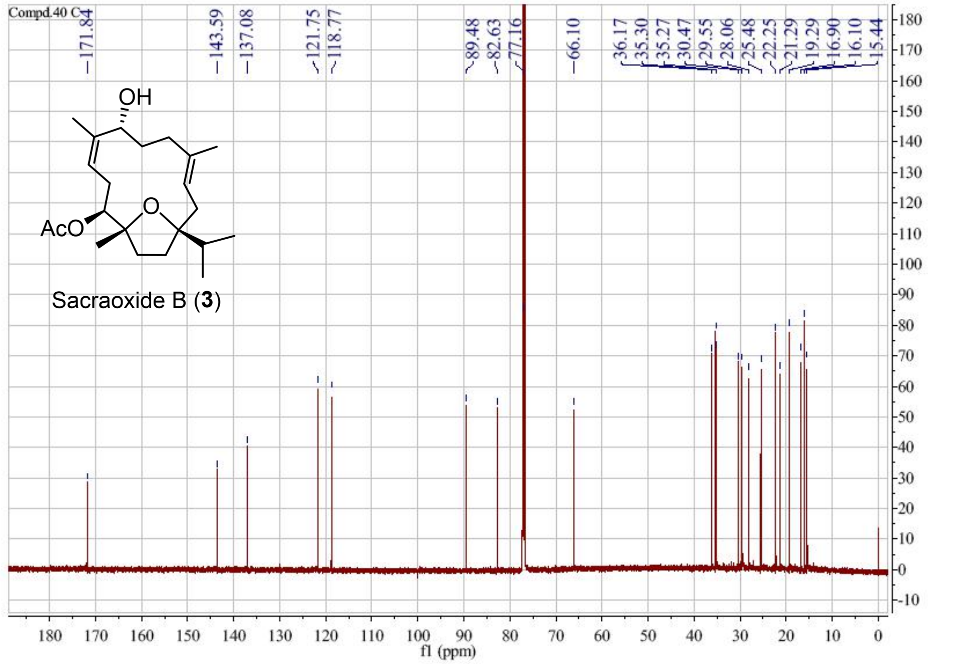

Supplementary Figure 5-2. ^{13}C NMR data of compound **3**

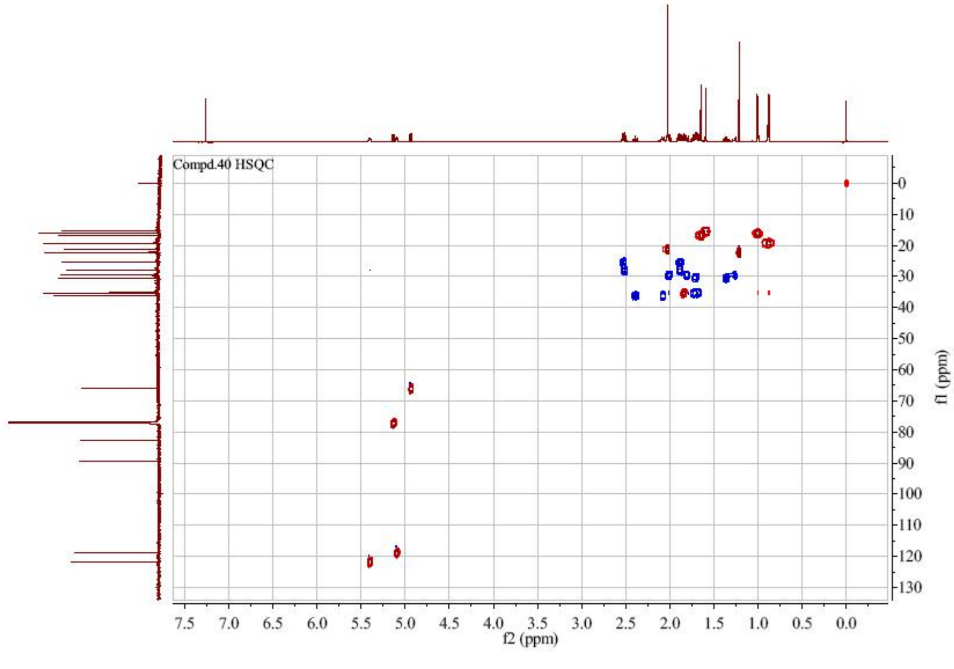

Supplementary Figure 5-3. HSQC Spectrum of compound **3**

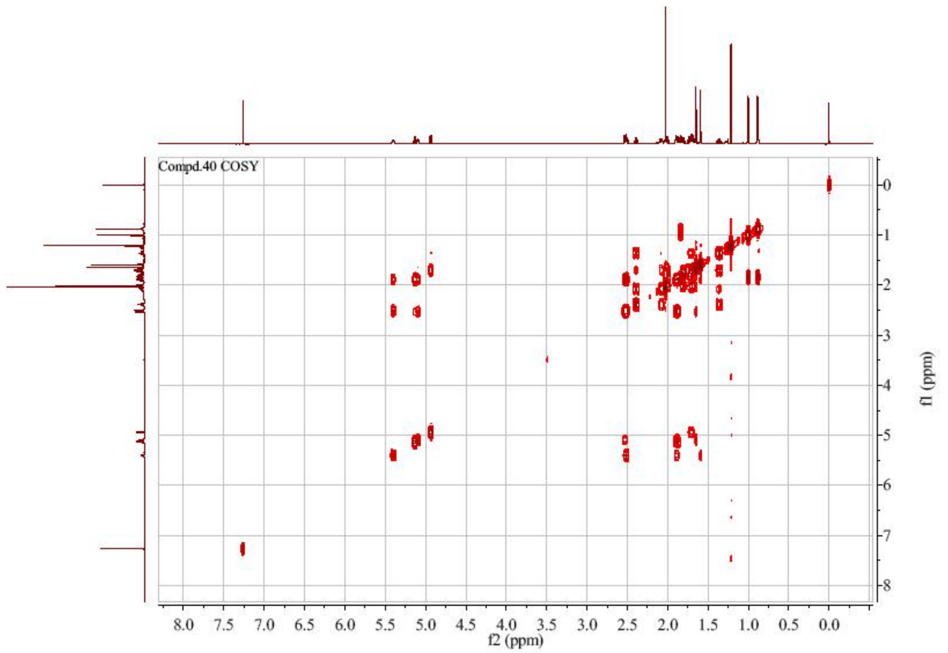

Supplementary Figure 5-4. ^{1}H-^{1}H COSY Spectrum of compound **3**

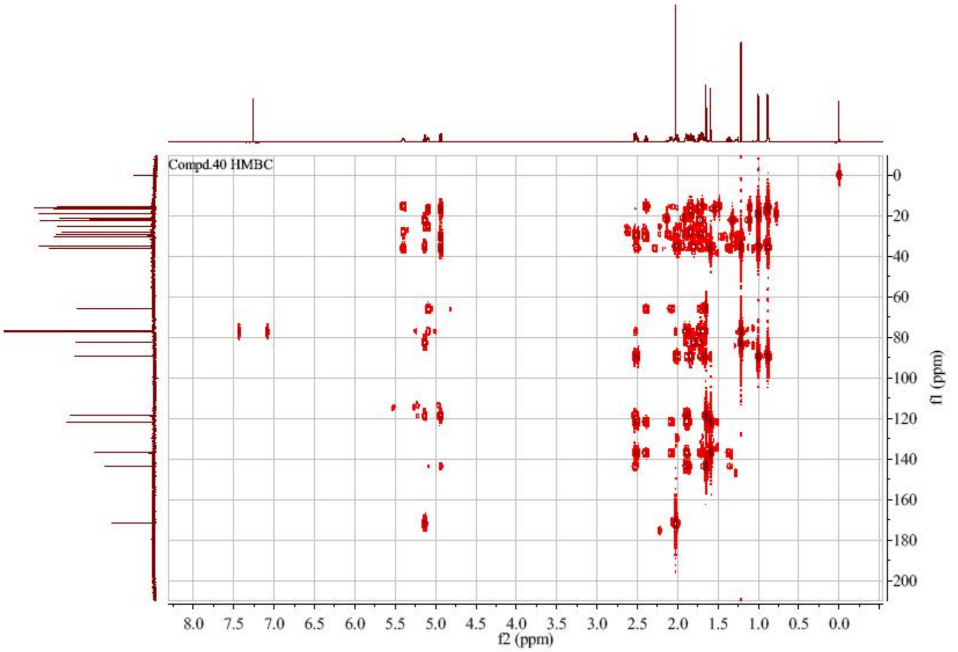

Supplementary Figure 5-5. HMBC Spectrum of compound **3**

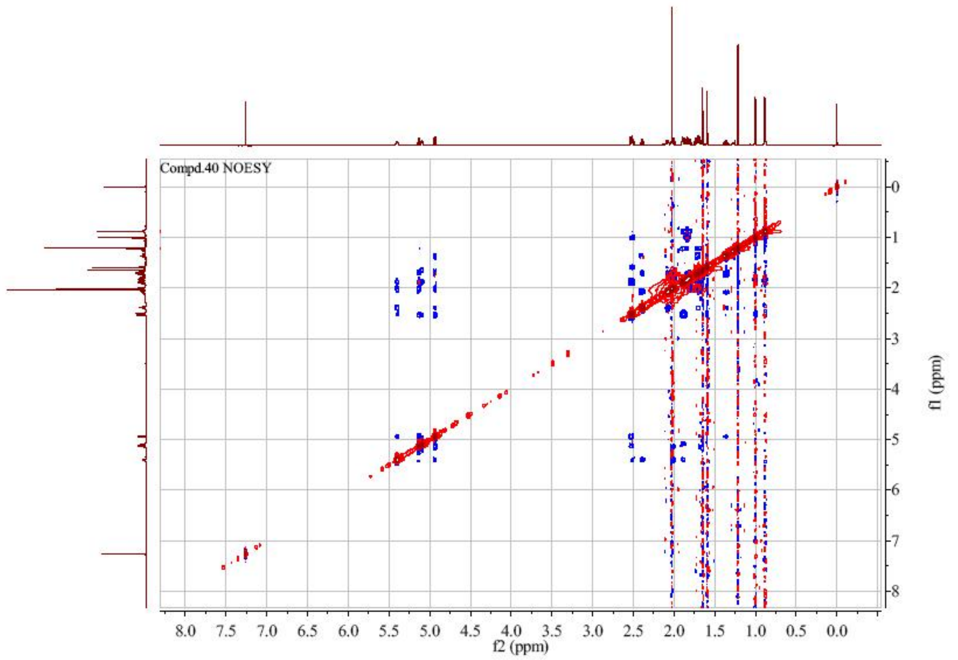

Supplementary Figure 5-6. NOESY Spectrum of compound **3**

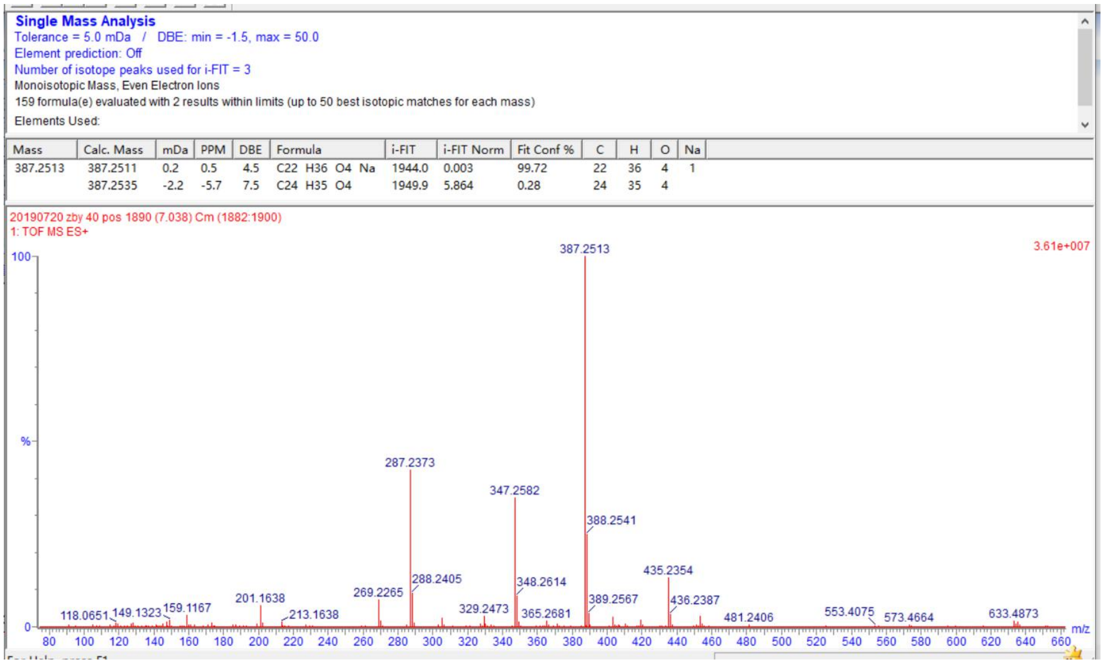

Supplementary Figure 5-7. HRESIMS data of compound **3**

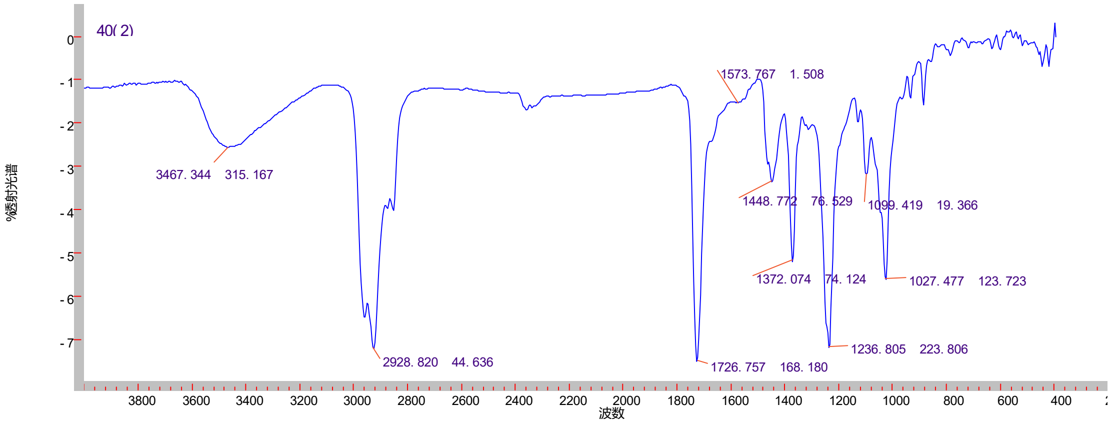

Supplementary Figure 5-8. IR Spectrum of compound **3**

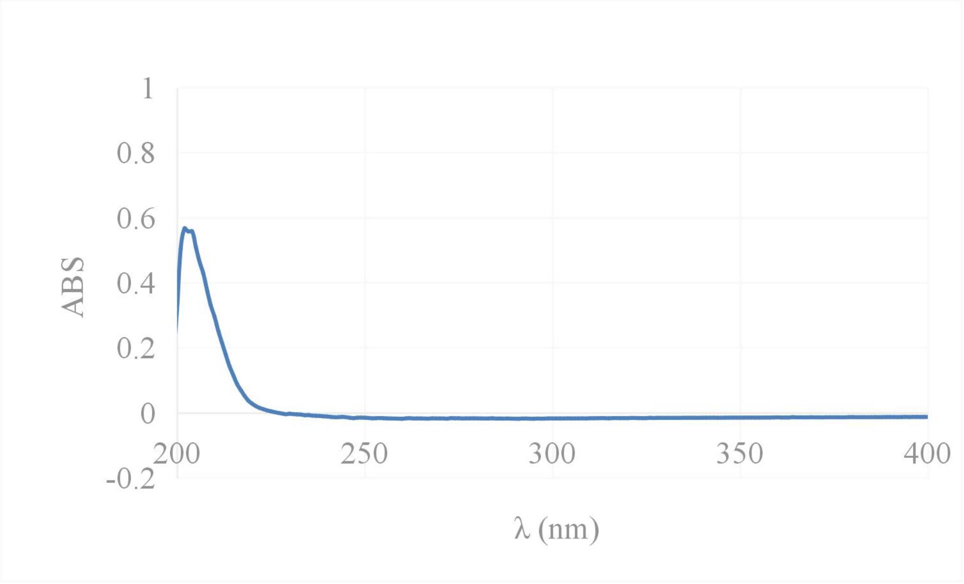

Supplementary Figure 5-9. UV Spectrum of compound **3**

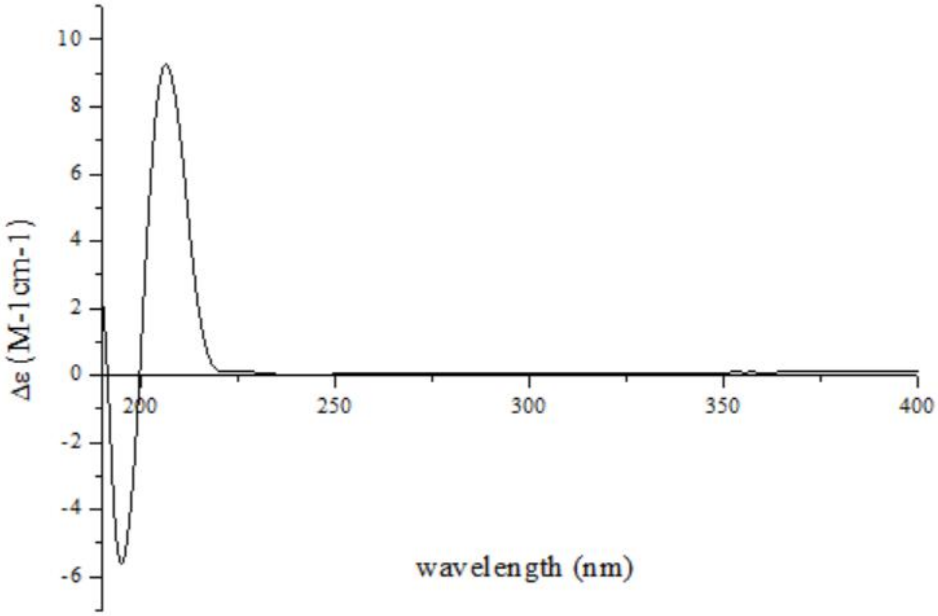

Supplementary Figure 5-10. ECD Spectrum of compound **3**

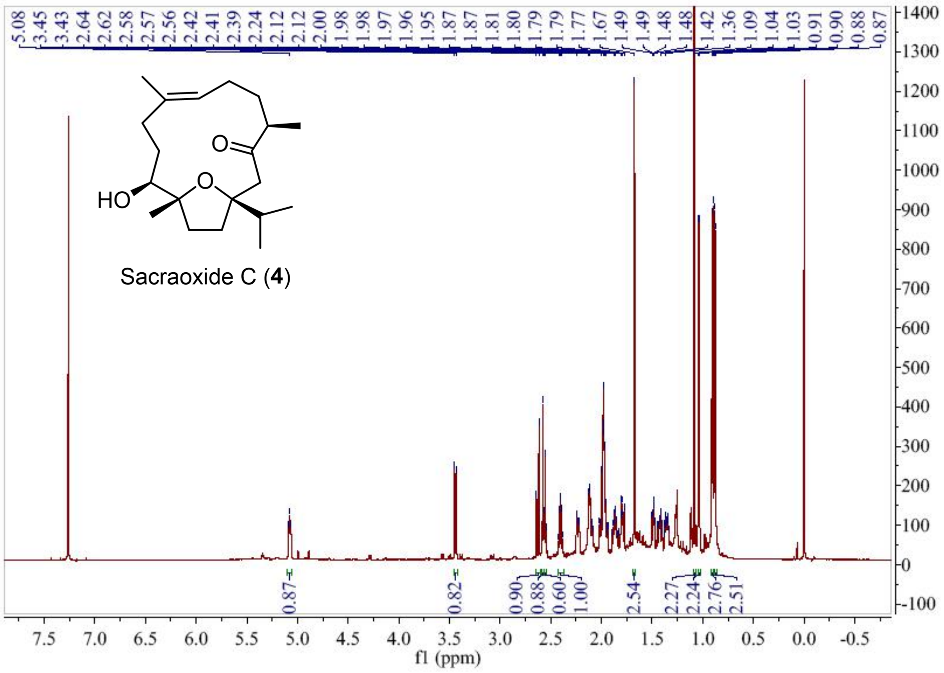

Supplementary Figure 6-1. ^{1}H NMR Spectrum of compound **4**

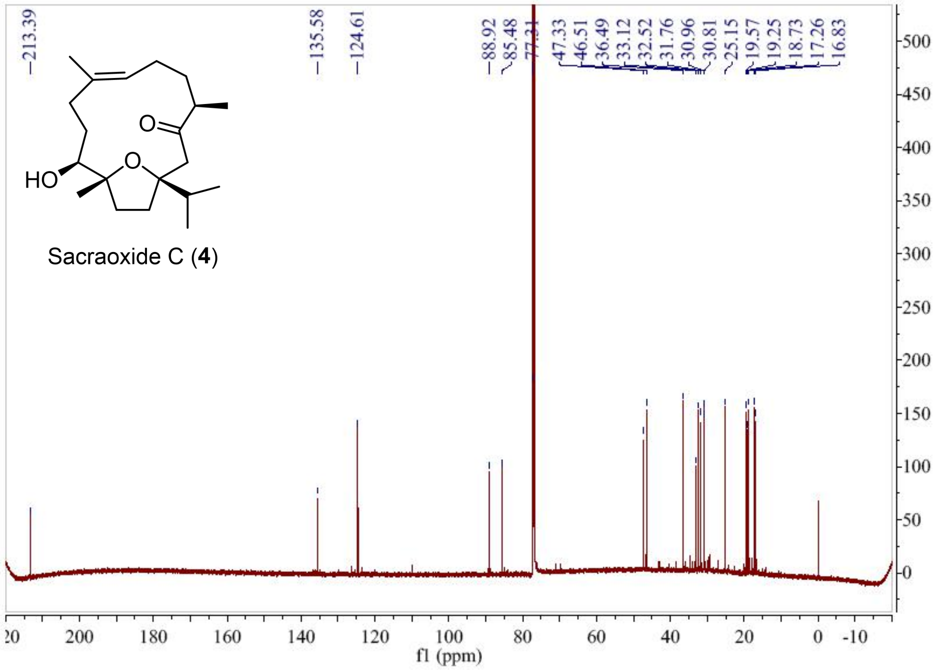

Supplementary Figure 6-2. ^{13}C NMR Spectrum of compound **4**

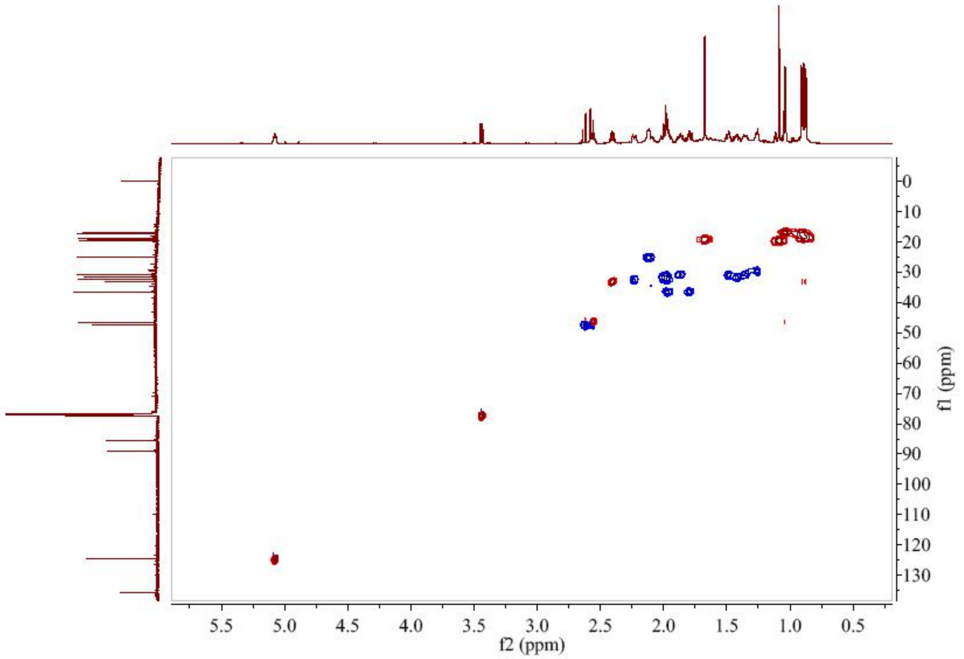

Supplementary Figure 6-3. HSQC Spectrum of compound **4**

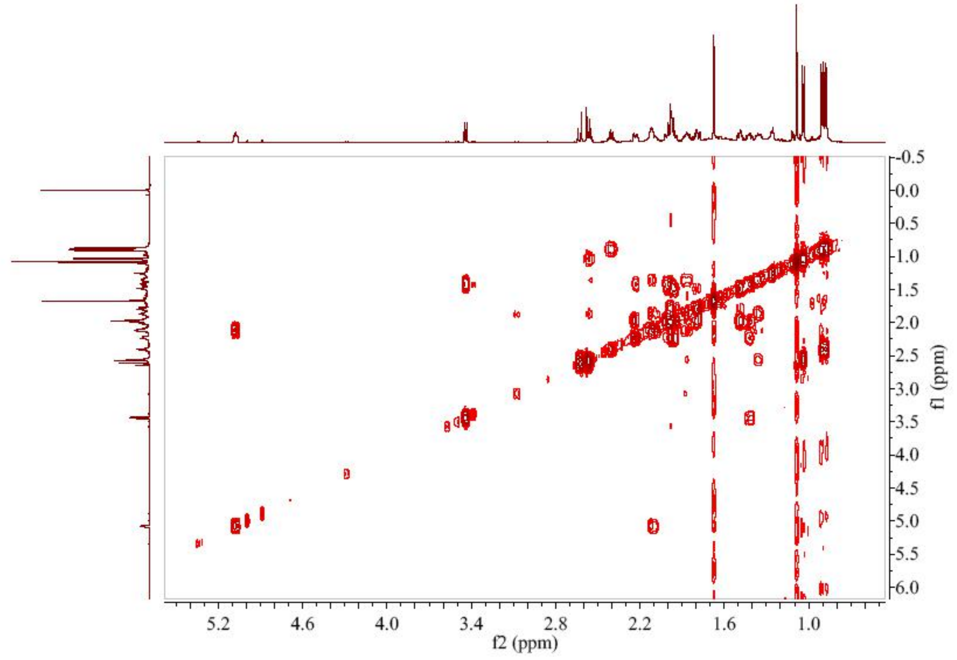

Supplementary Figure 6-4. ^{1}H-^{1}H COSY Spectrum of compound **4**

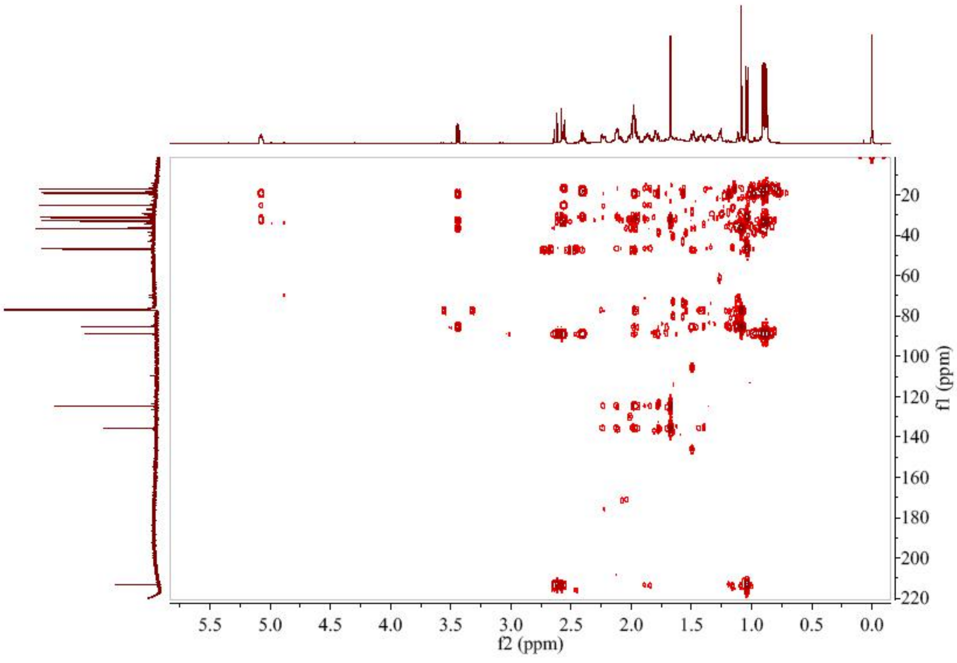

Supplementary Figure 6-5. HMBC Spectrum of compound **4**

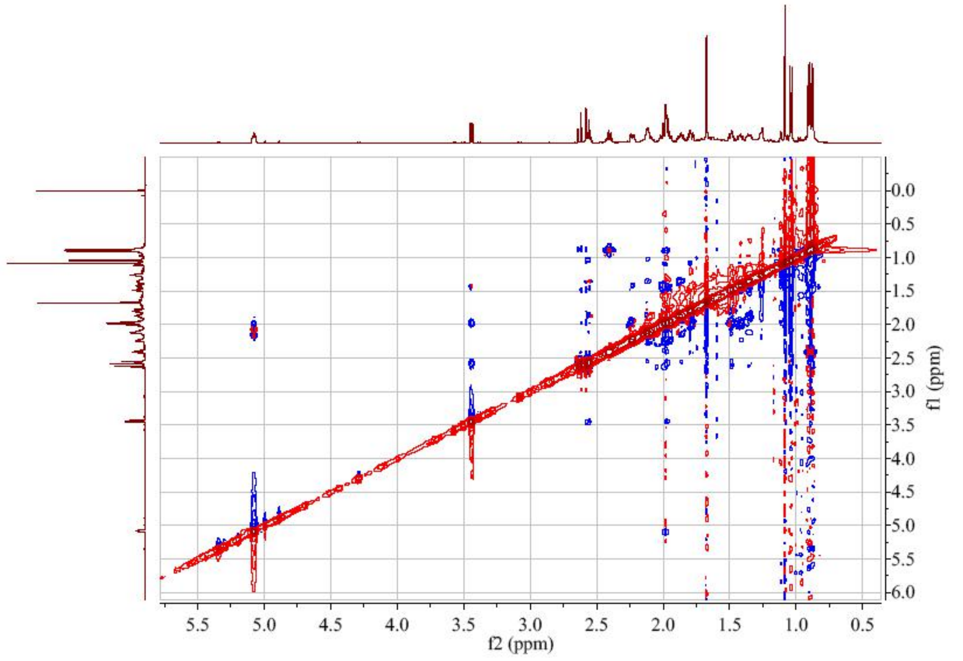

Supplementary Figure 6-6. NOESY Spectrum of compound **4**

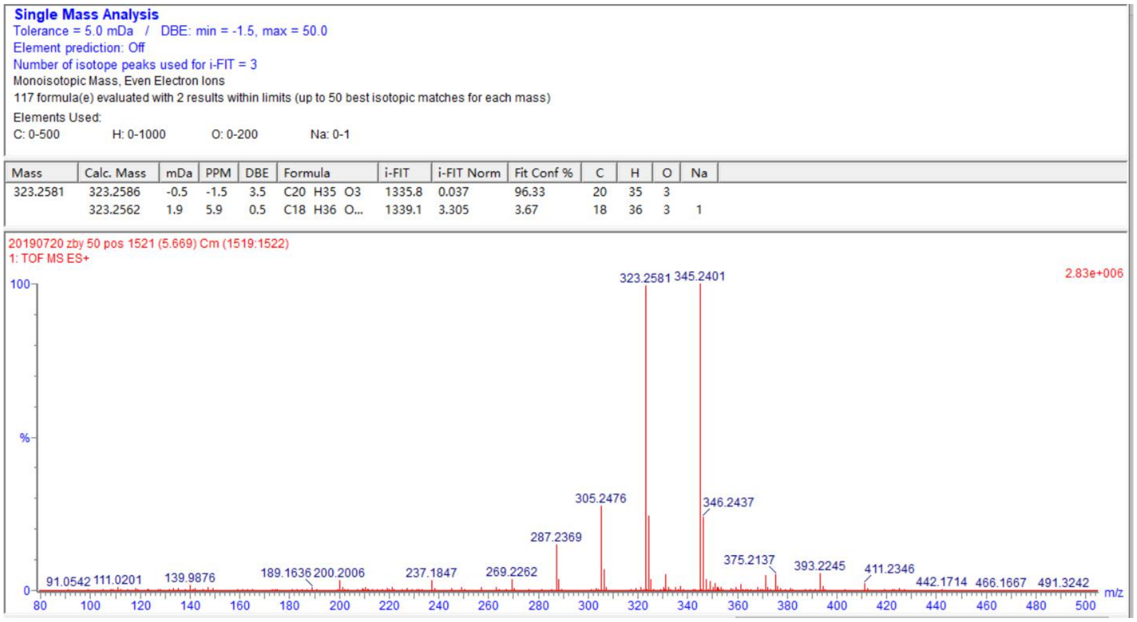

Mass	Calc. Mass	mDa	PPM	DBE	Formula	i-FIT	i-FIT Norm	Fit Conf %	C	H	O	Na
323.2581	323.2586	-0.5	-1.5	3.5	C20 H35 O3	1335.8	0.037	96.33	20	35	3	
	323.2562	1.9	5.9	0.5	C18 H36 O...	1339.1	3.305	3.67	18	36	3	1

Supplementary Figure 6-7. HRESIMS data of compound **4**

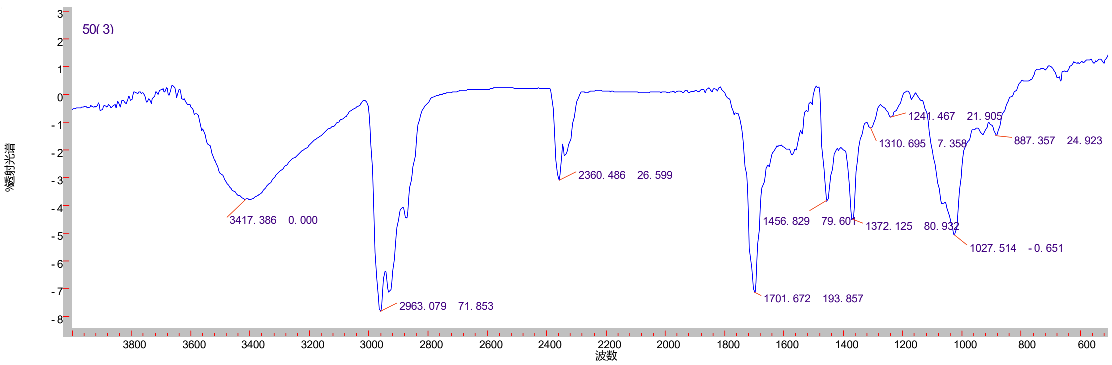

Supplementary Figure 6-8. IR Spectrum of compound **4**

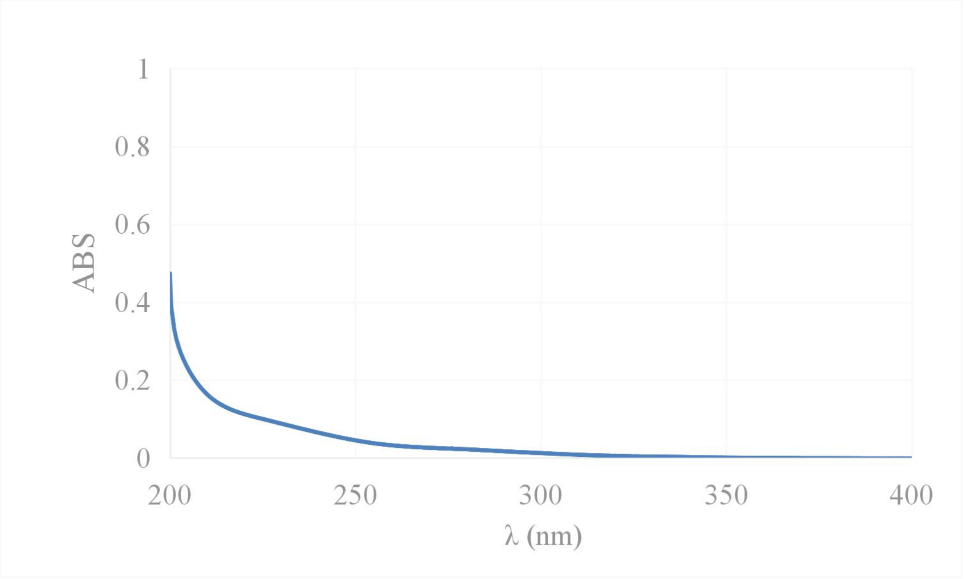

Supplementary Figure 6-9. UV Spectrum of compound **4**

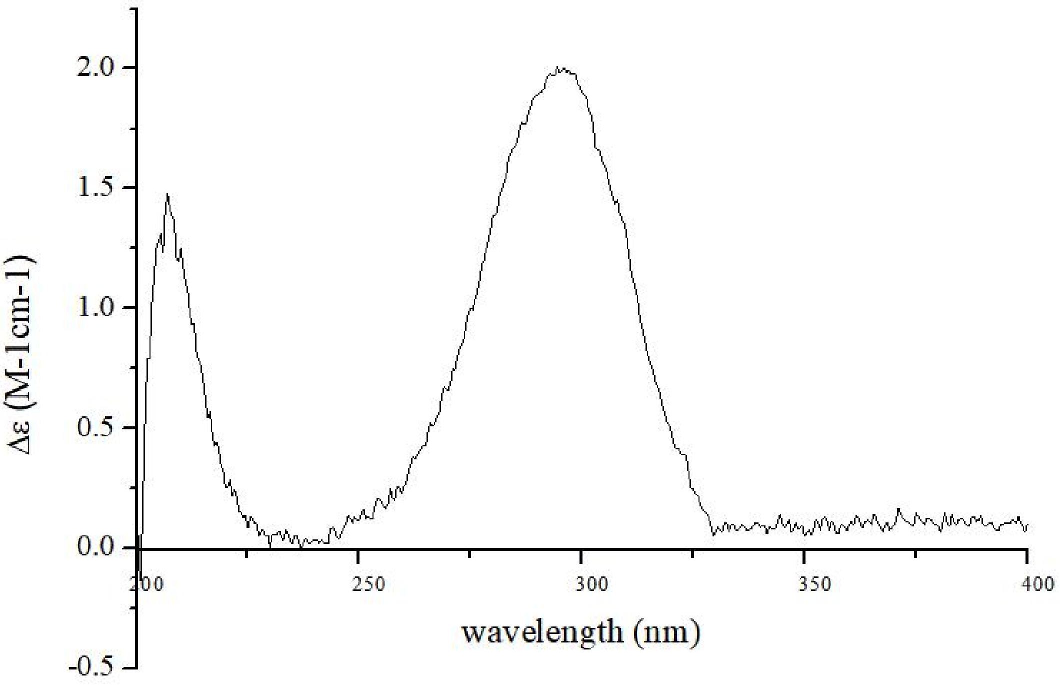

Supplementary Figure 6-10. ECD Spectrum of compound **4**

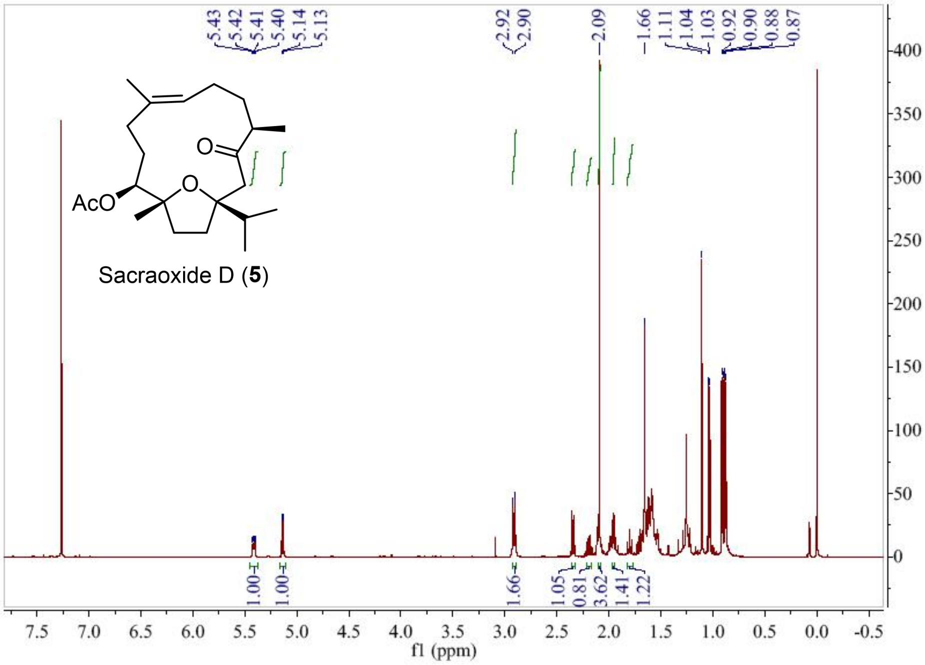

Supplementary Figure 7-1. 1H NMR Spectrum of compound **5**

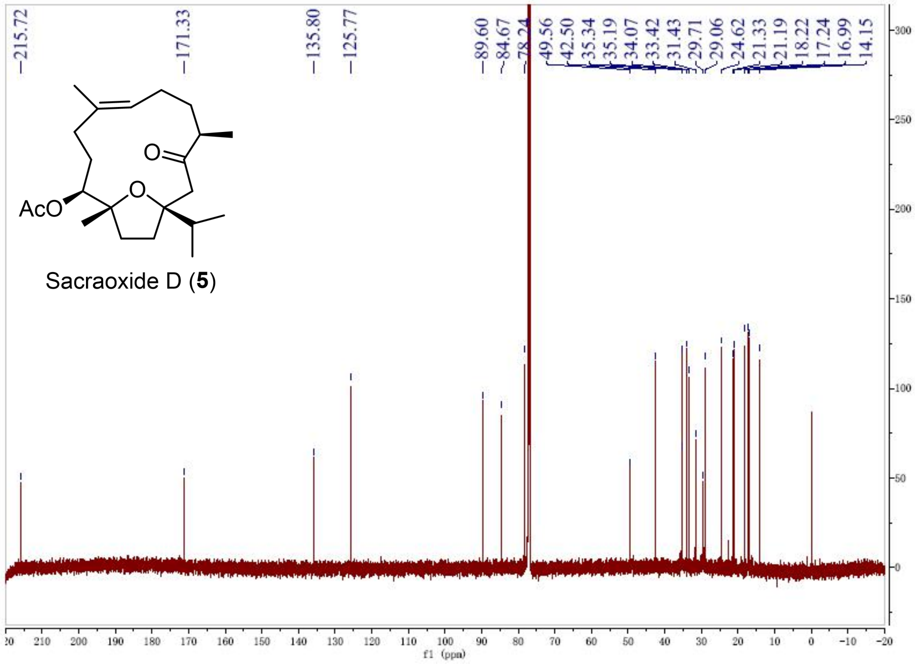

Supplementary Figure 7-2. ^{13}C NMR Spectrum of compound **5**

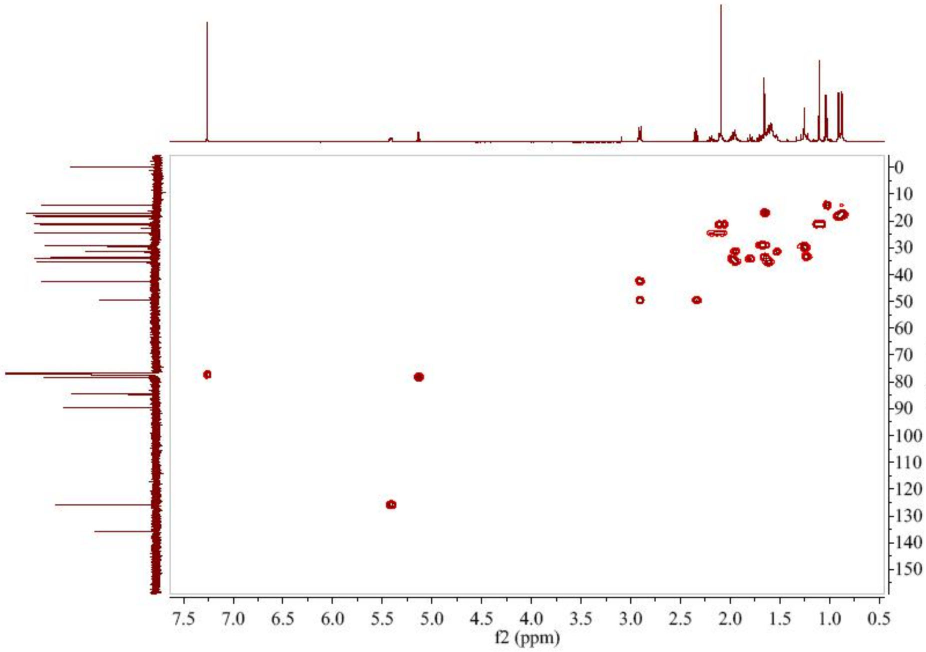

Supplementary Figure 7-3. HSQC Spectrum of compound **5**

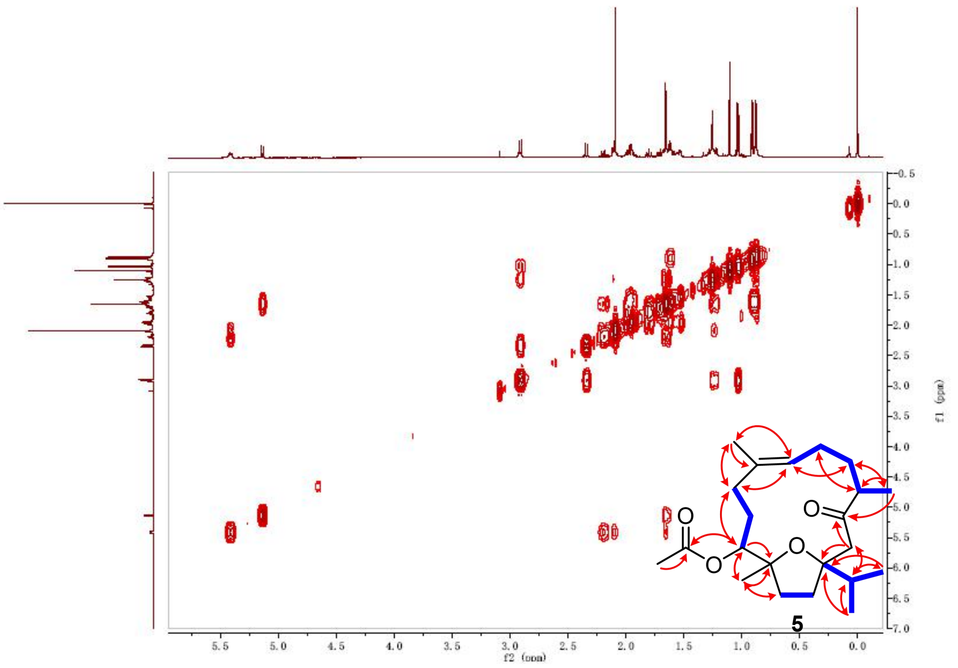

Supplementary Figure 7-4. ^{1}H-1H COSY Spectrum of compound **5**

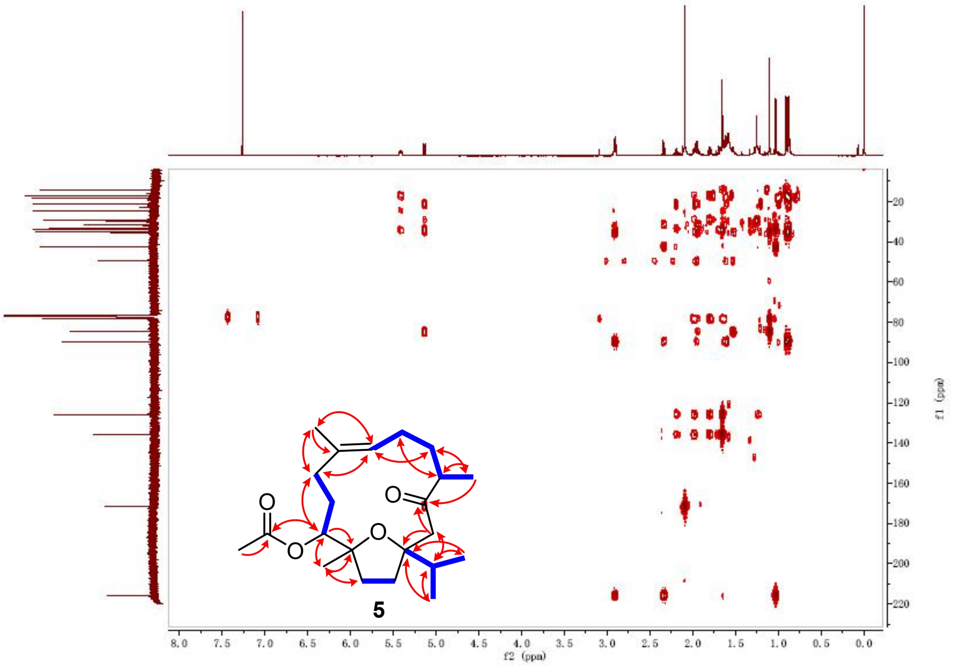

Supplementary Figure 7-5. HMBC Spectrum of compound **5**

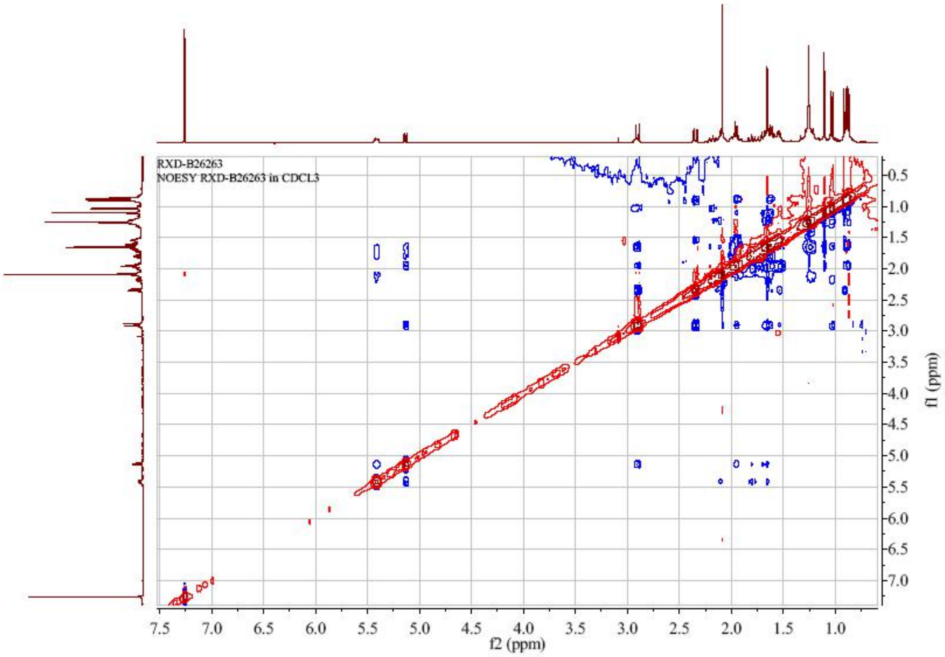

Supplementary Figure 7-6. NOESY Spectrum of compound **5**

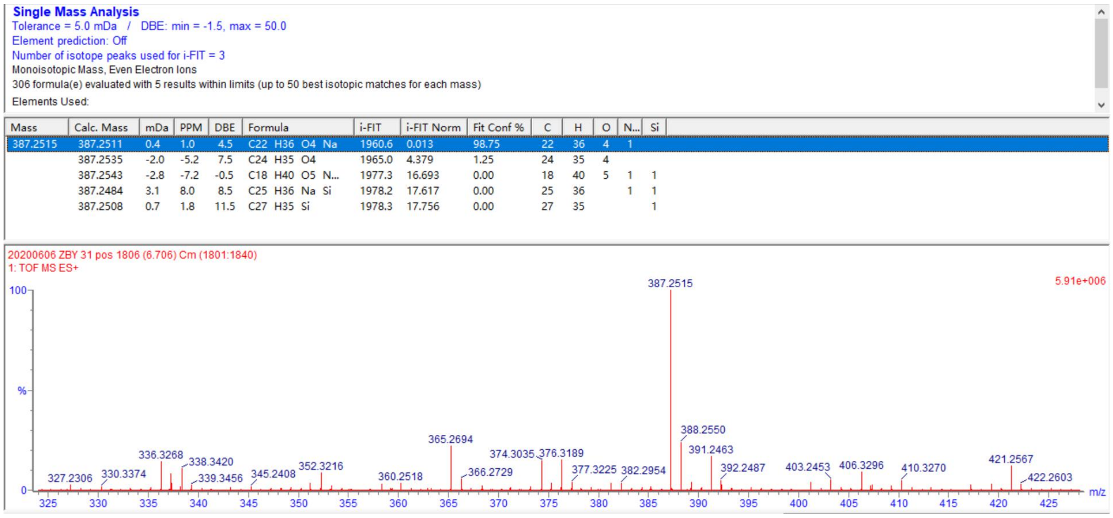

Supplementary Figure 7-7. HRESIMS data of compound **5**

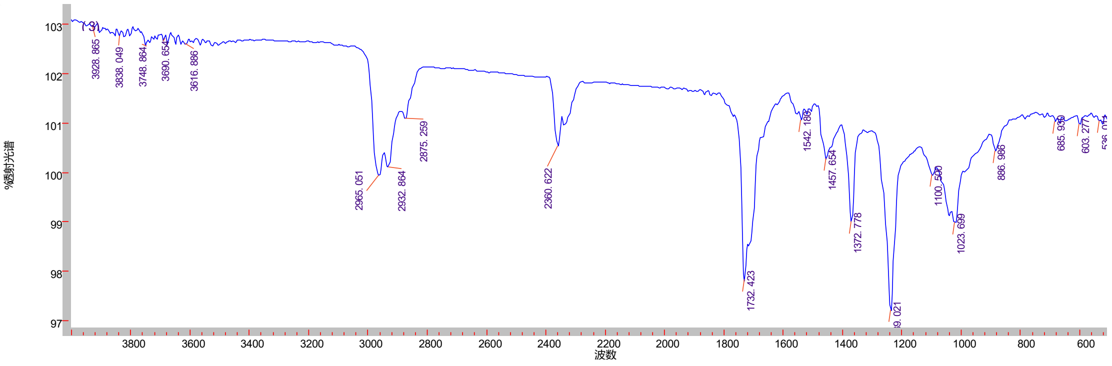

Supplementary Figure 7-8. IR Spectrum for compound **5**

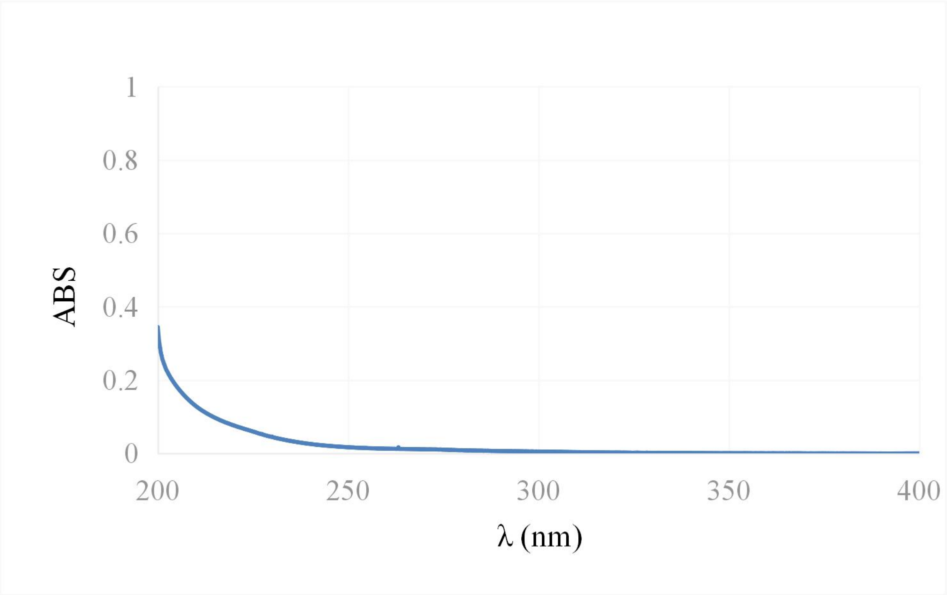

Supplementary Figure 7-9. UV Spectrum for compound **5**

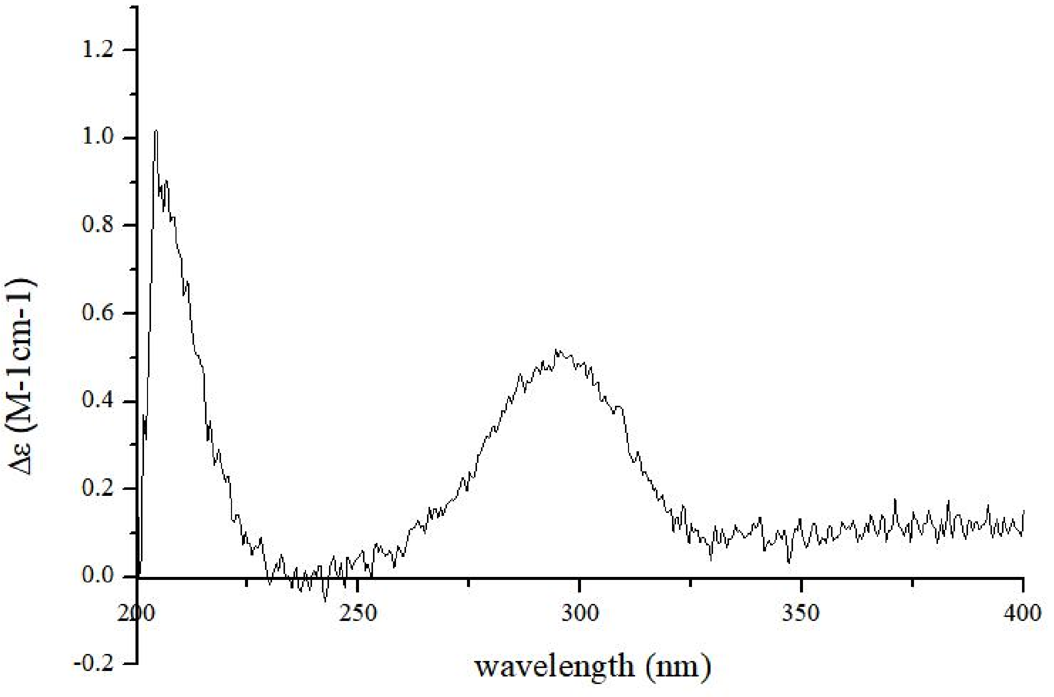

Supplementary Figure 7-10. ECD Spectrum for compound **5**

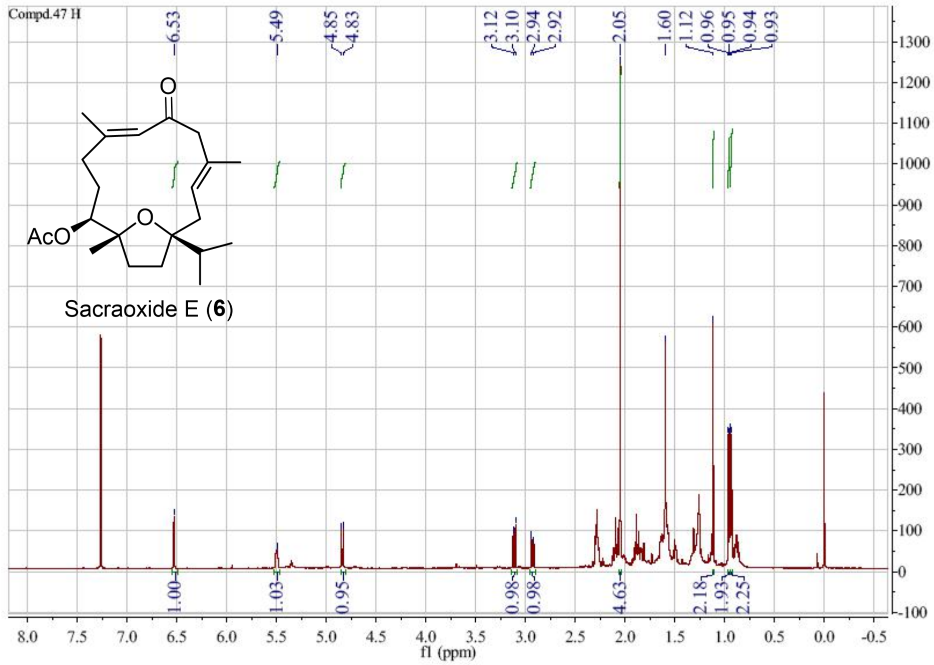

Supplementary Figure 8-1. ^{1}H NMR Spectrum of compound **6**

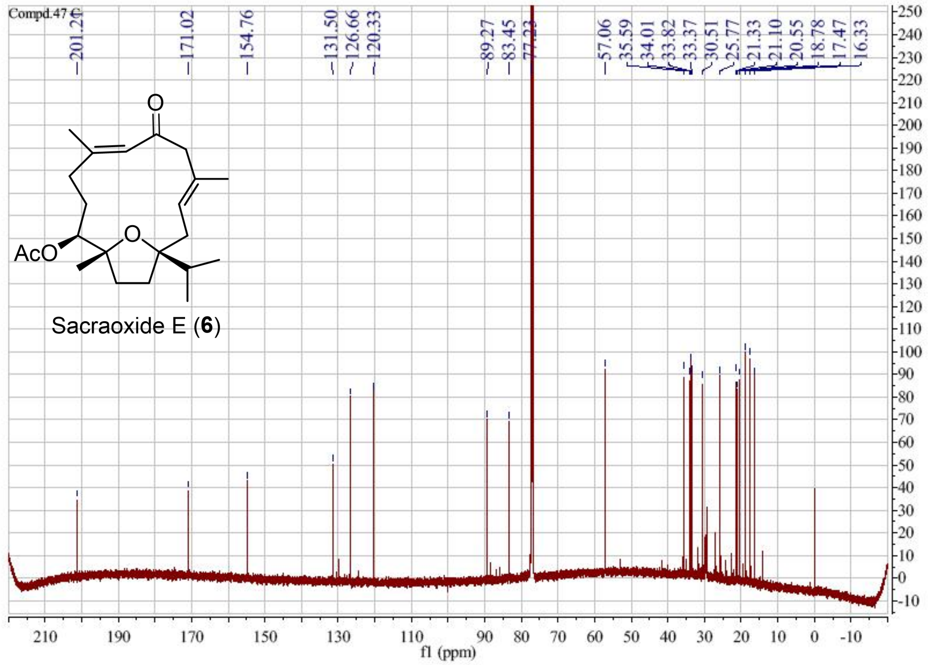

Supplementary Figure 8-2. ^{13}C NMR Spectrum of compound **6**

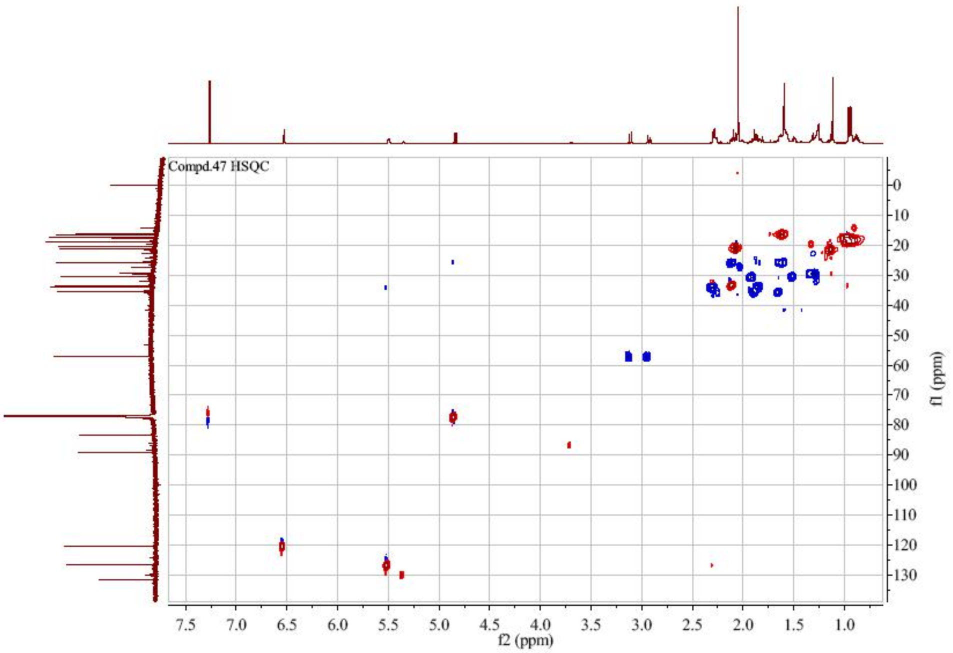

Supplementary Figure 8-3. HSQC Spectrum of compound **6**

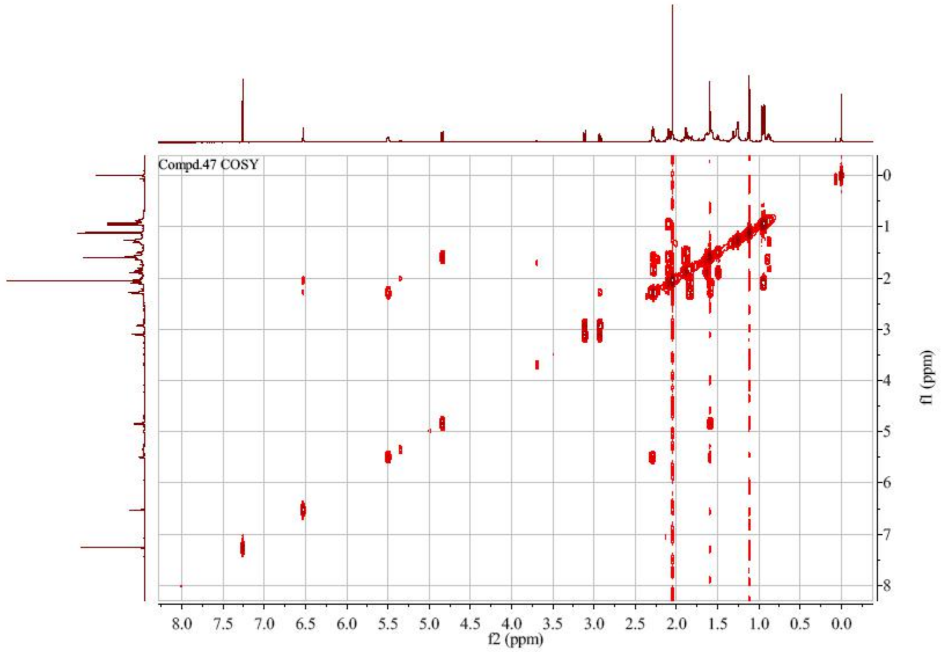

Supplementary Figure 8-4. ^{1}H-^{1}H COSY Spectrum of compound **6**

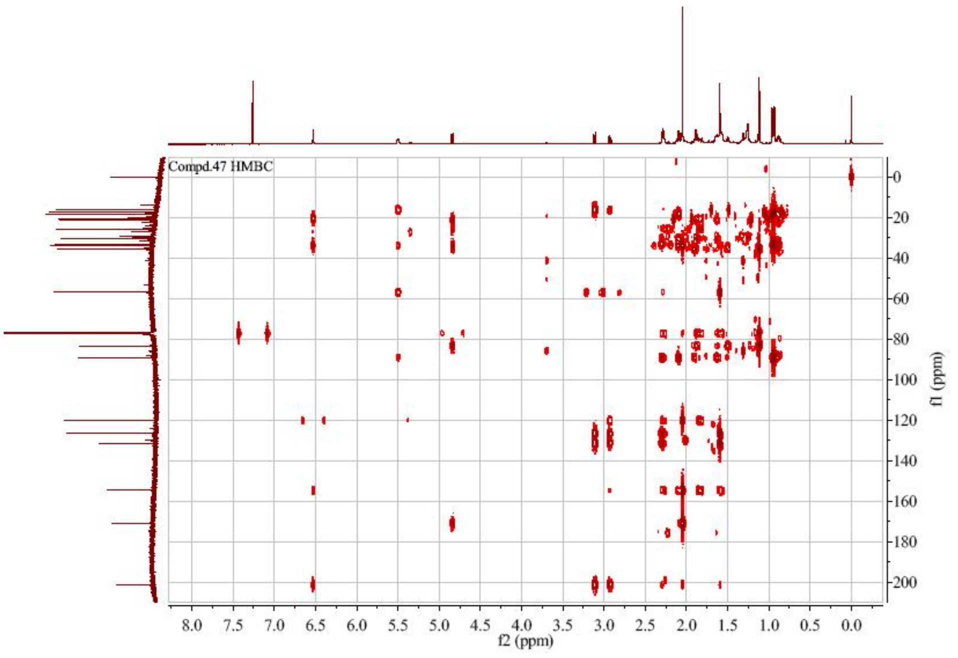

Supplementary Figure 8-5. HMBC Spectrum of compound **6**

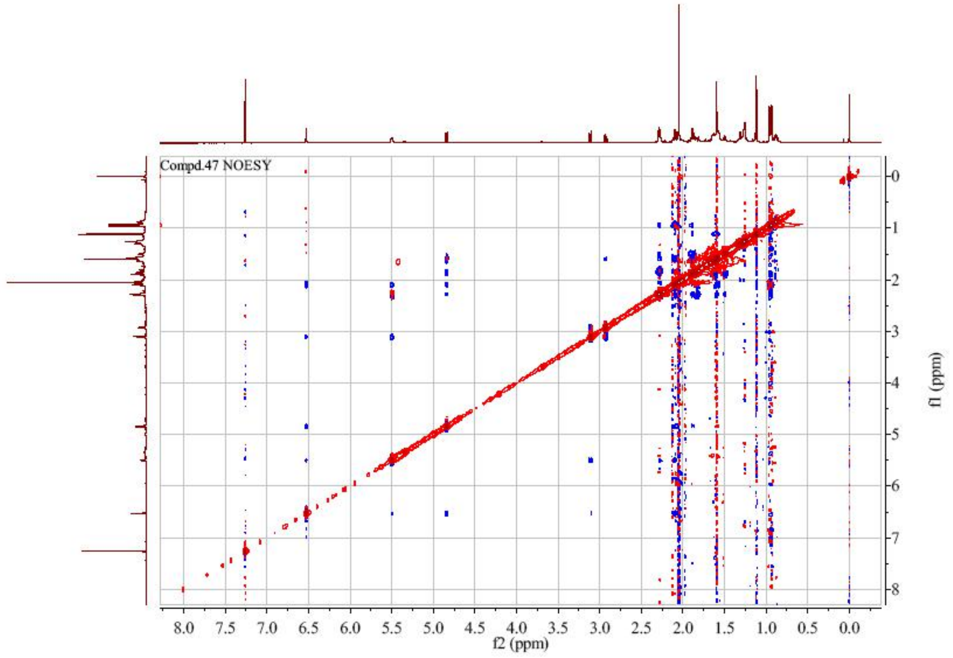

Supplementary Figure 8-6. NOESY Spectrum of compound **6**

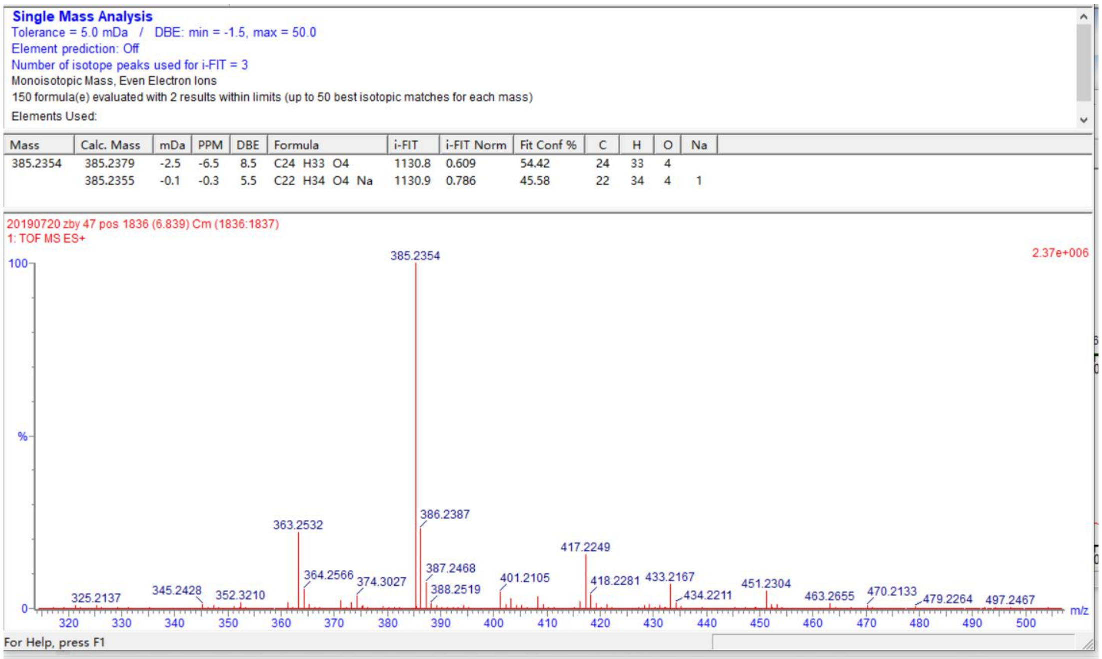

Supplementary Figure 8-7. HRESIMS data of compound **6**

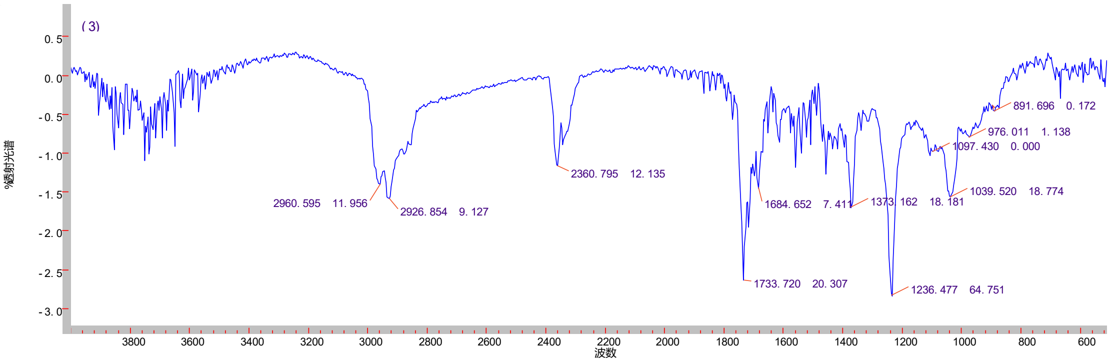

Supplementary Figure 8-8. IR Spectrum for compound **6**

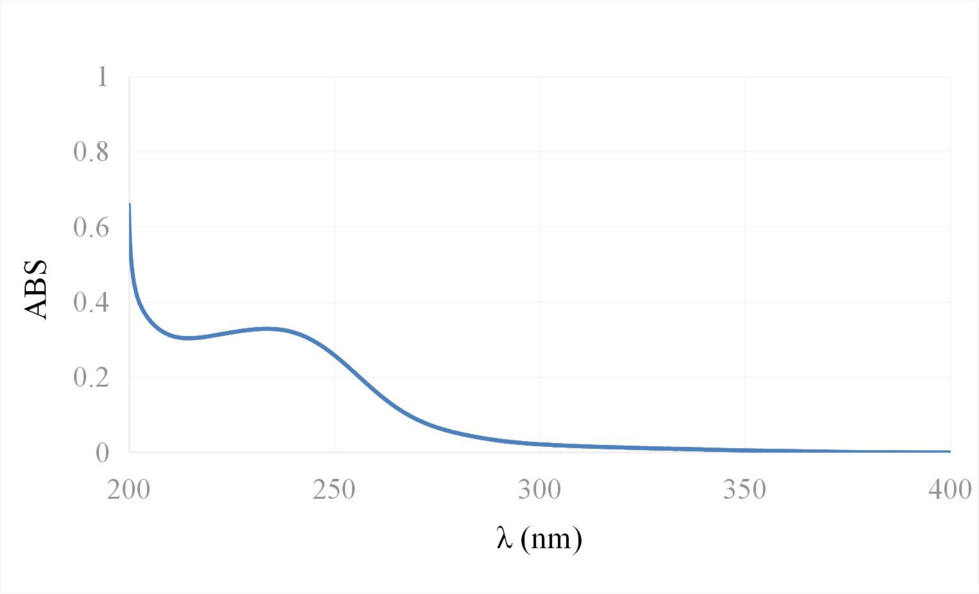

Supplementary Figure 8-9. UV Spectrum for compound **6**

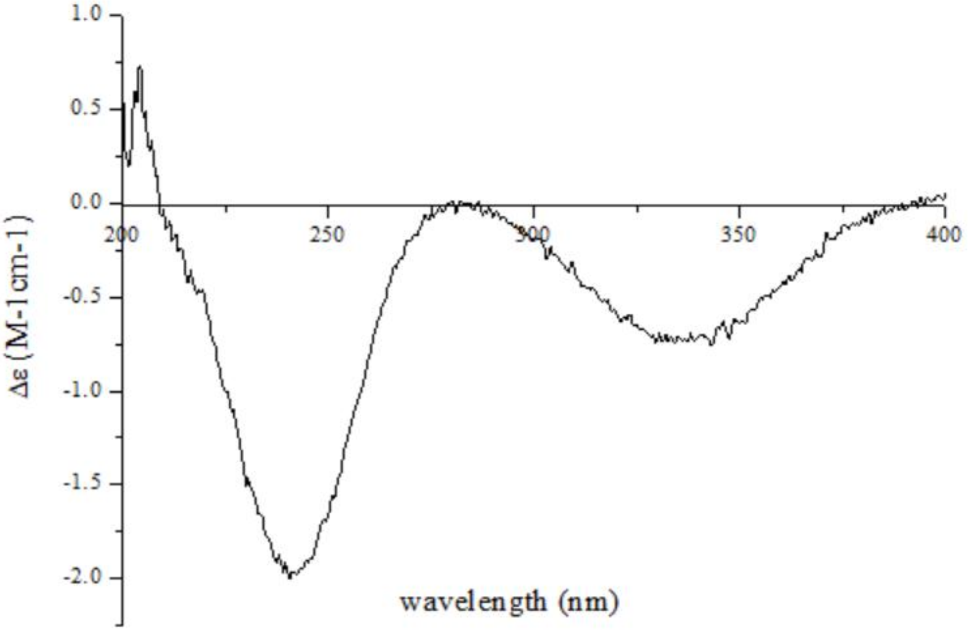

Supplementary Figure 8-10. ECD Spectrum for compound **6**

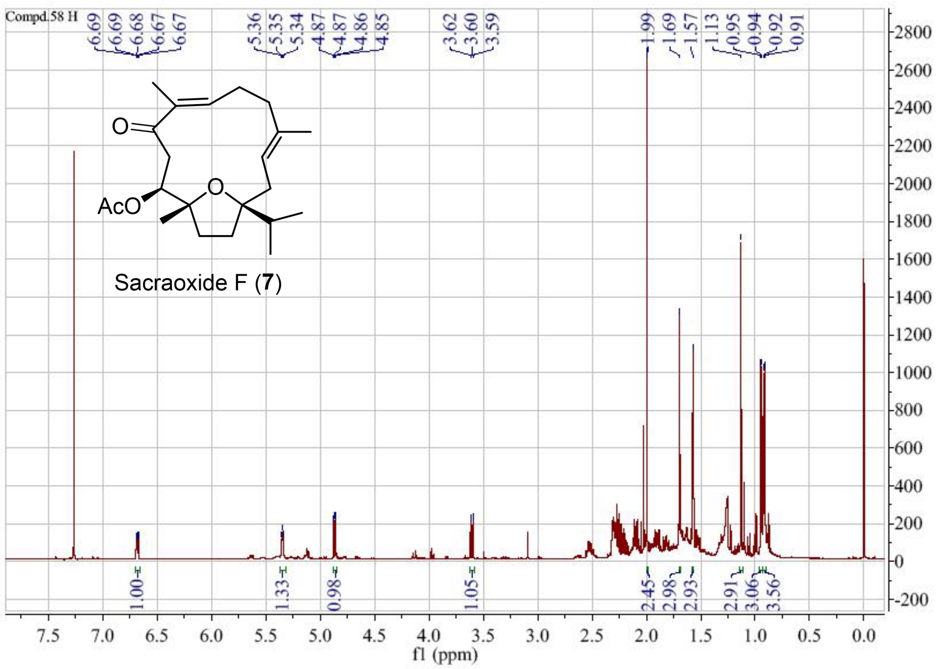

Supplementary Figure 9-1. ^{1}H NMR Spectrum of compound **7**

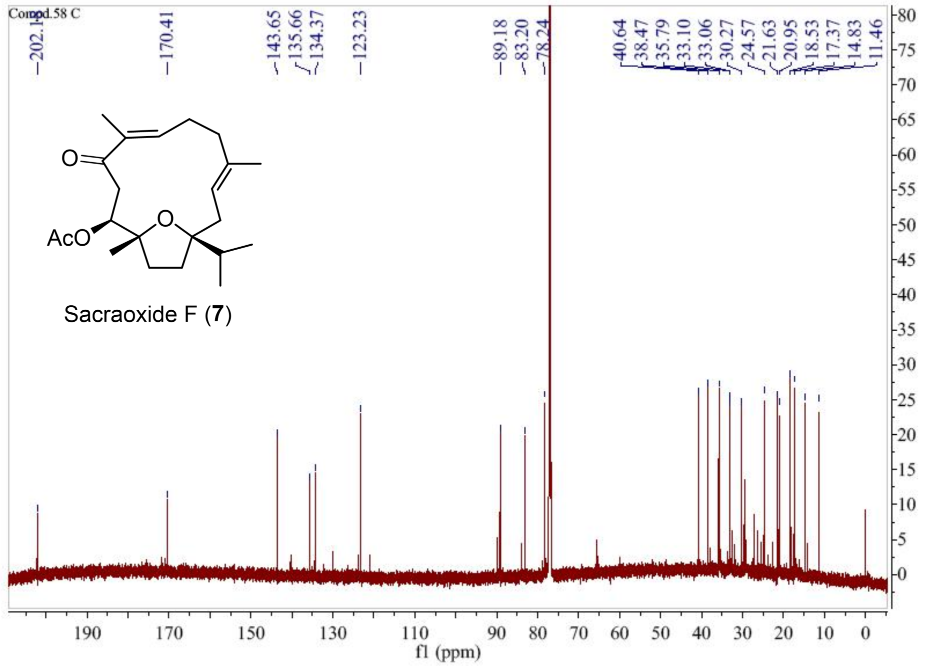

Supplementary Figure 9-2. ^{13}C NMR Spectrum of compound **7**

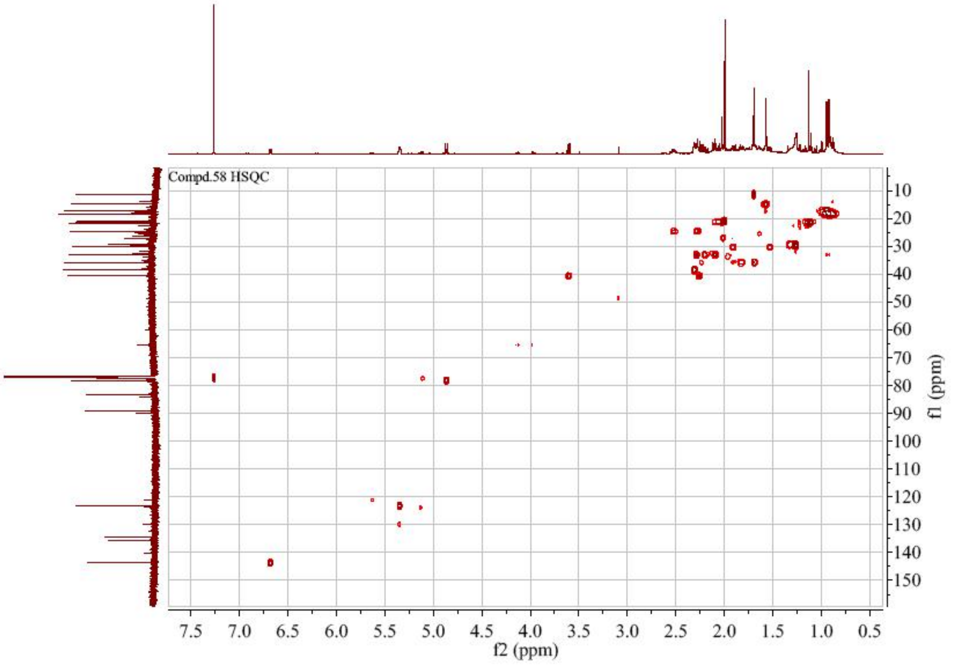

Supplementary Figure 9-3. HSQC Spectrum of compound **7**

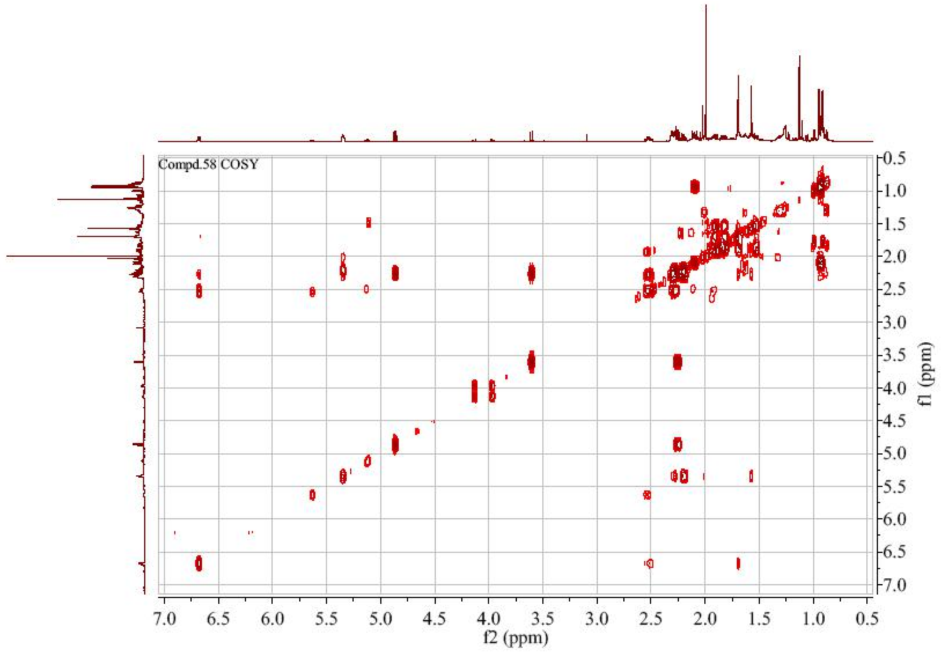

Supplementary Figure 9-4. ^{1}H-^{1}H COSY Spectrum of compound **7**

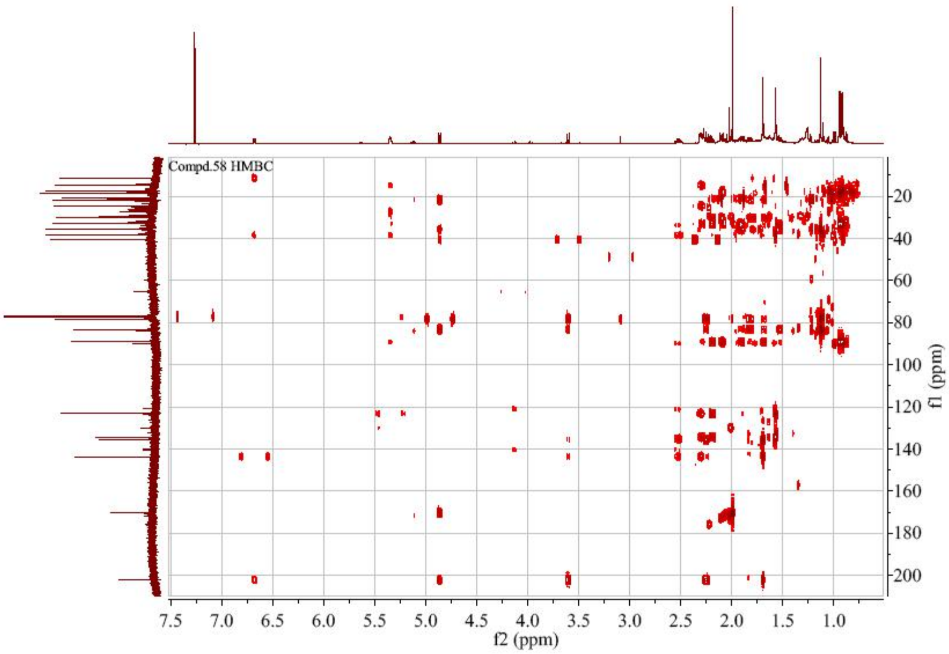

Supplementary Figure 9-5. HMBC Spectrum of compound **7**

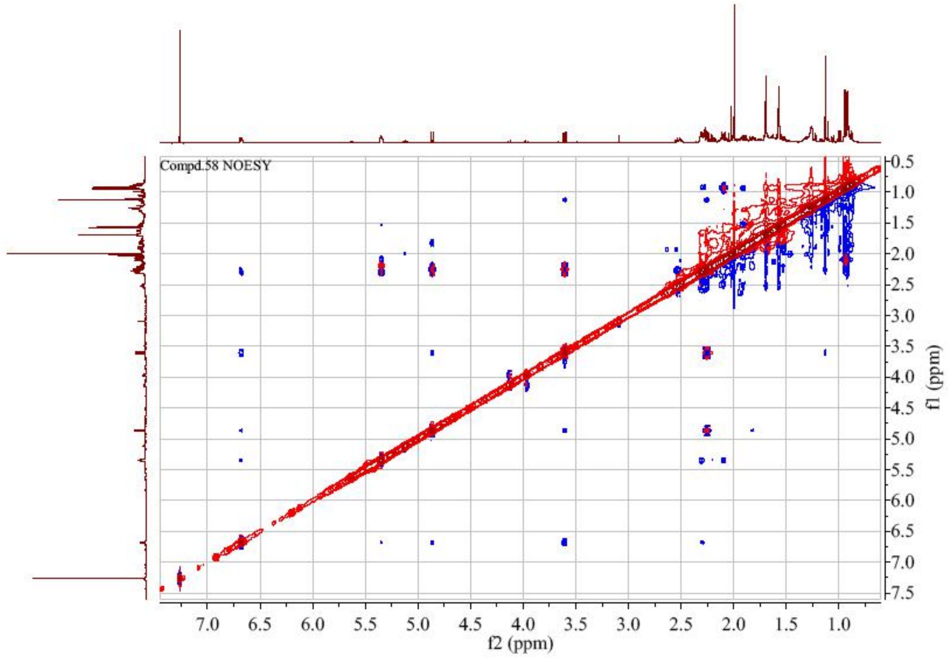

Supplementary Figure 9-6. NOESY Spectrum of compound **7**

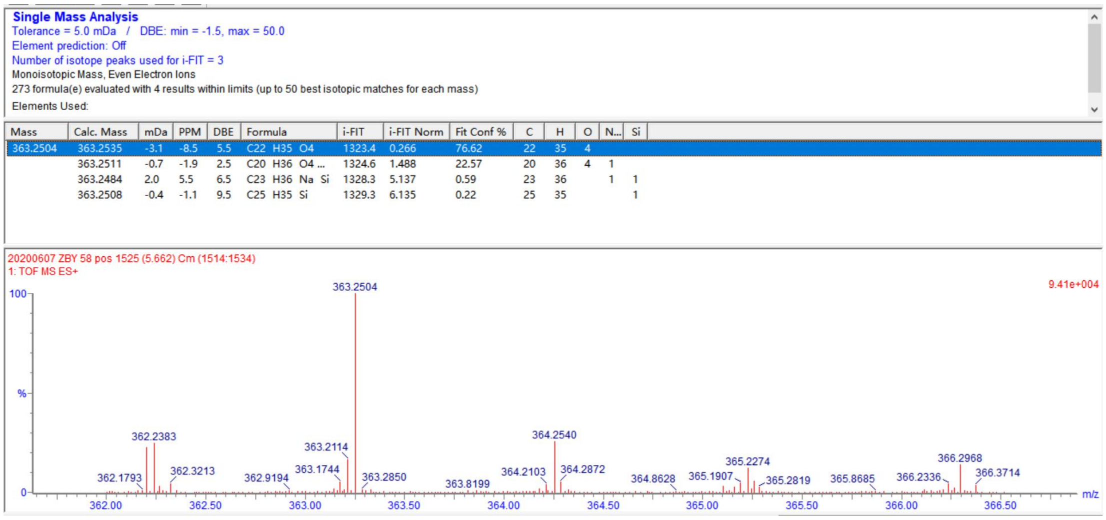

Supplementary Figure 9-7. HRESIMS for compound **7**

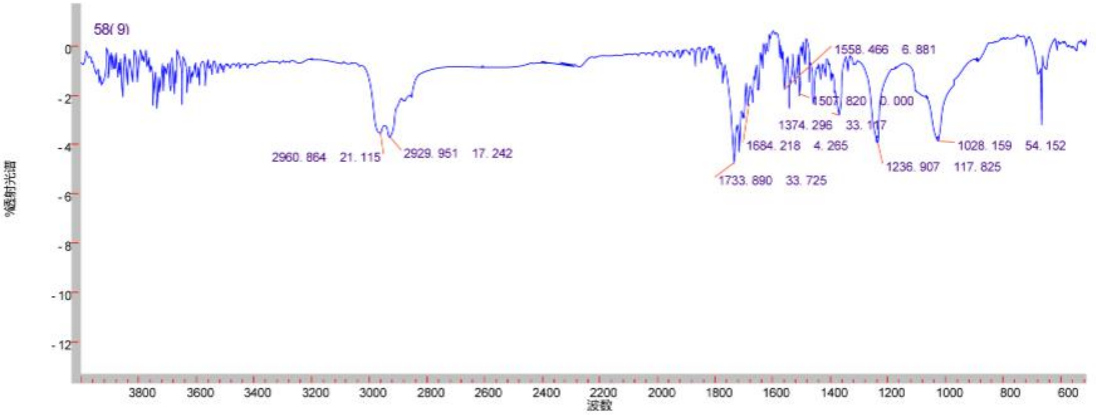

Supplementary Figure 9-8. IR Spectrum for compound **7**

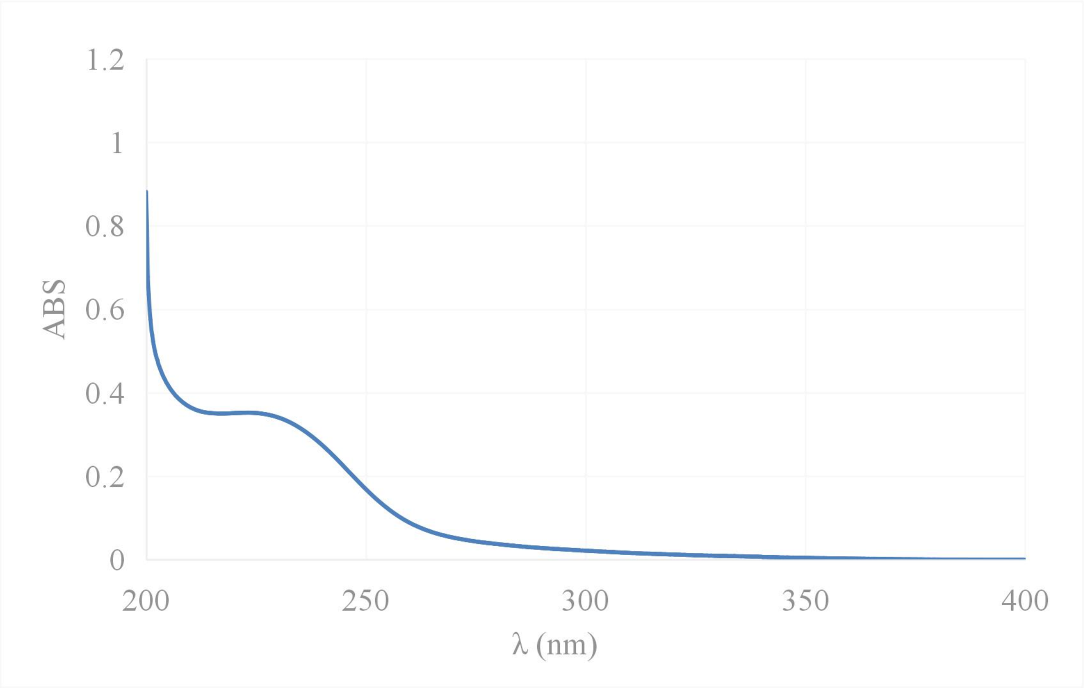

Supplementary Figure 9-9. UV Spectrum for compound **7**

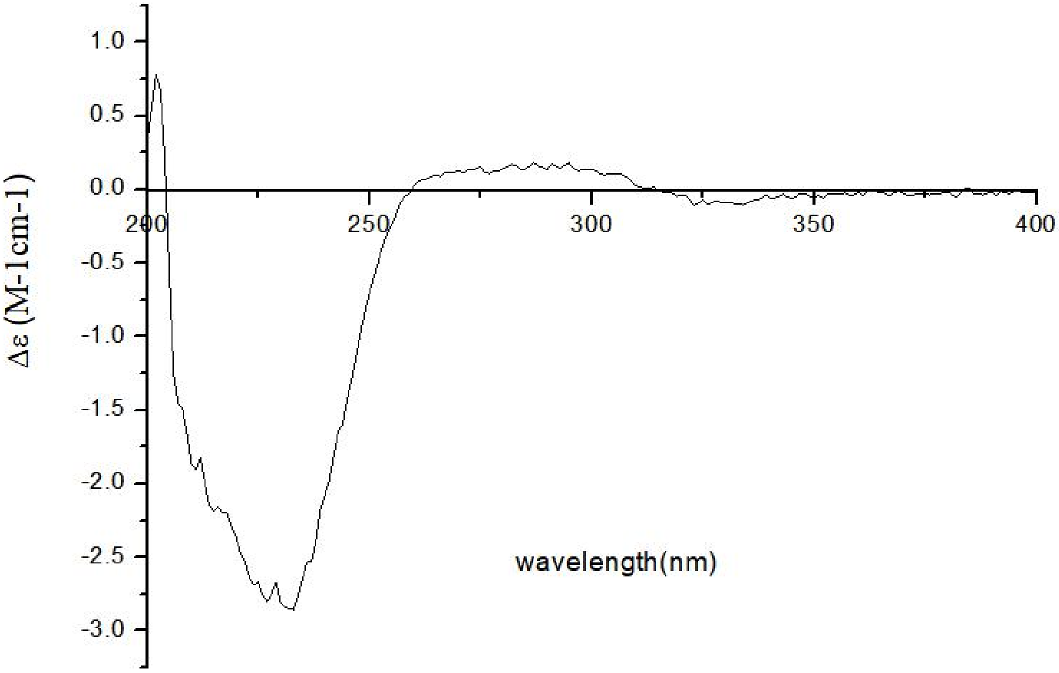

Supplementary Figure 9-10. ECD Spectrum for compound **7**

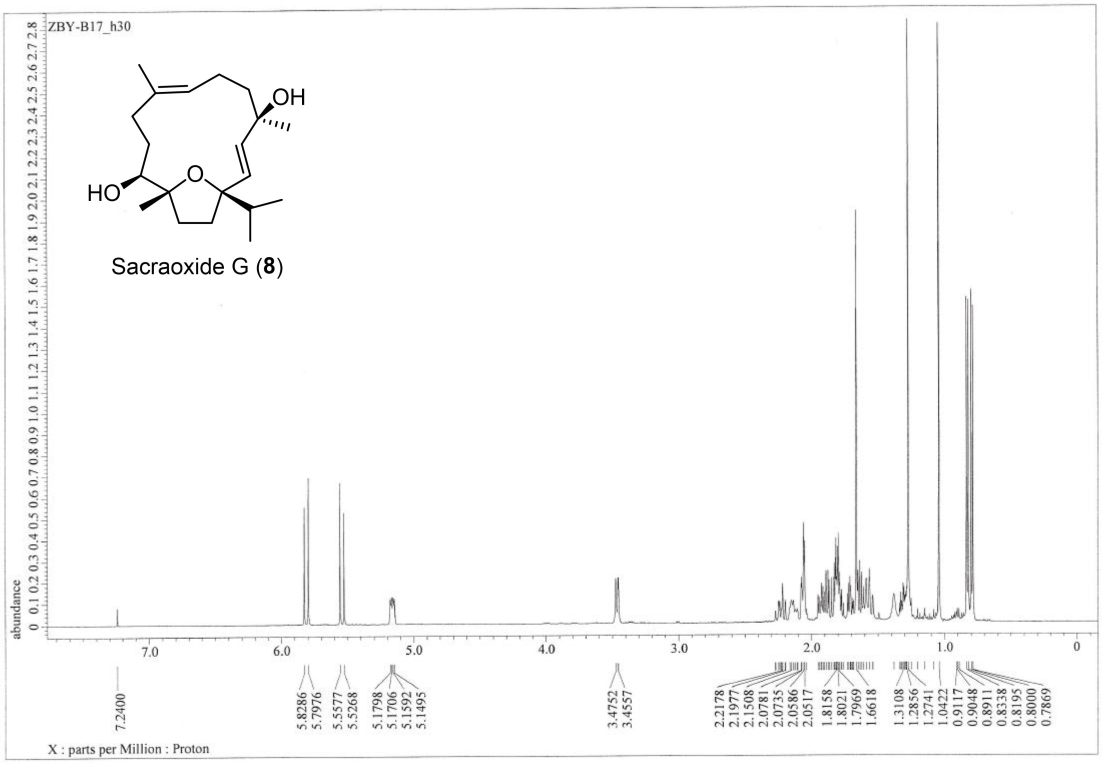

Supplementary Figure 10-1. ^{1}H NMR Spectrum of compound **8**

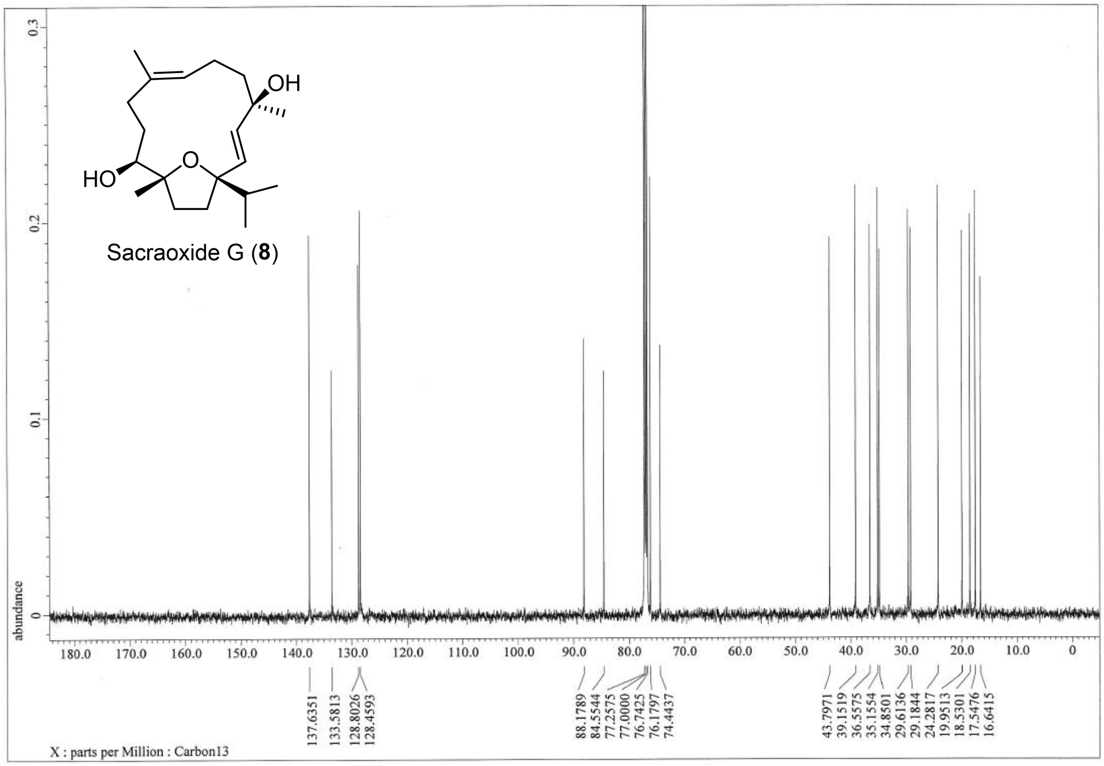

Supplementary Figure 10-2. ^{13}C NMR Spectrum of compound **8**

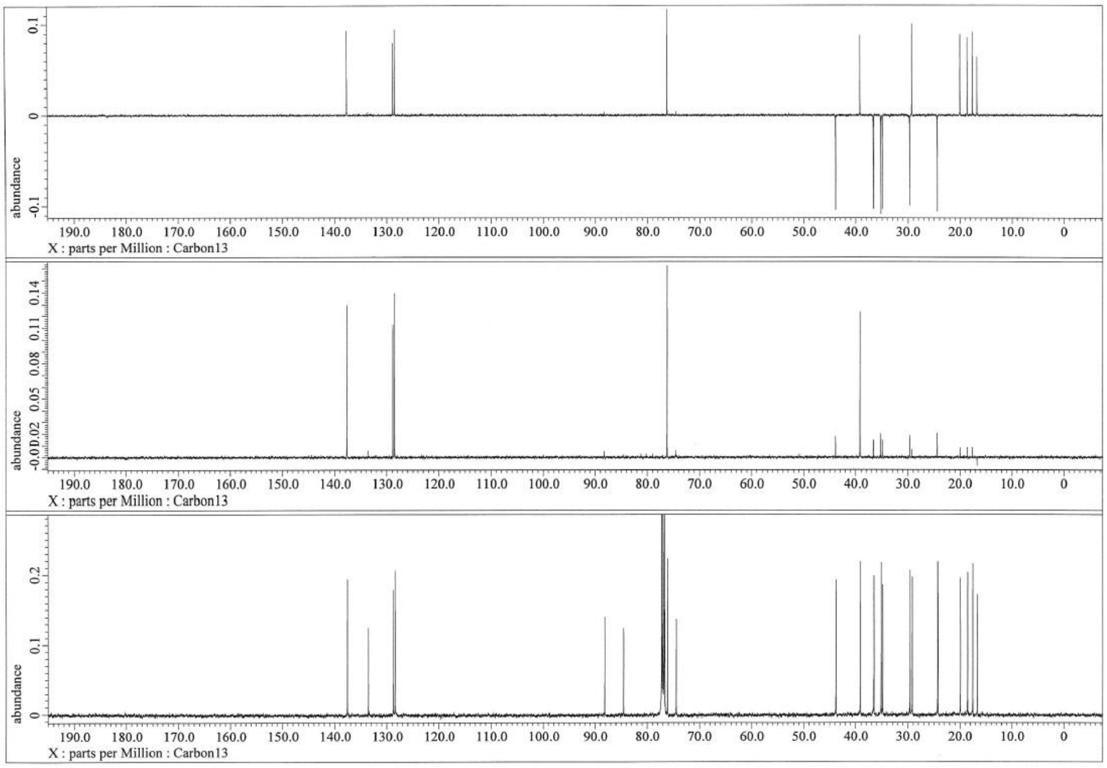

Supplementary Figure 10-3. DEPT Spectrum of compound **8**

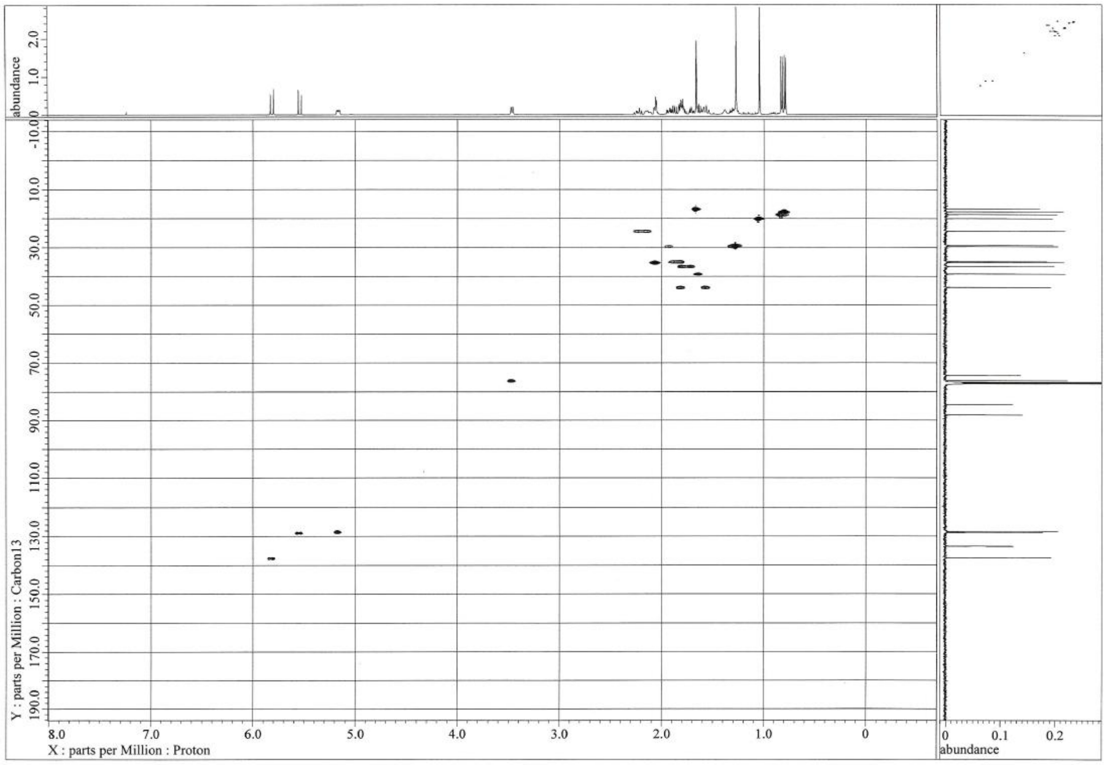

Supplementary Figure 10-4. HSQC Spectrum of compound **8**

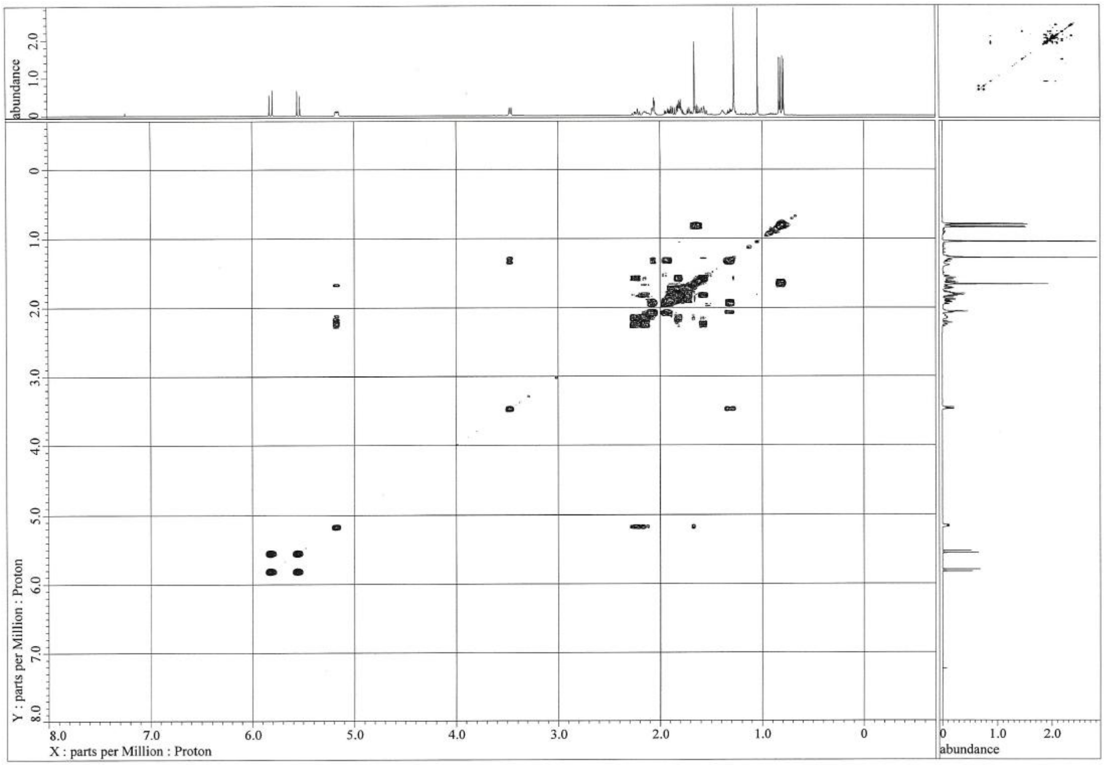

Supplementary Figure 10-5. ^1H-^1H COSY Spectrum of compound **8**

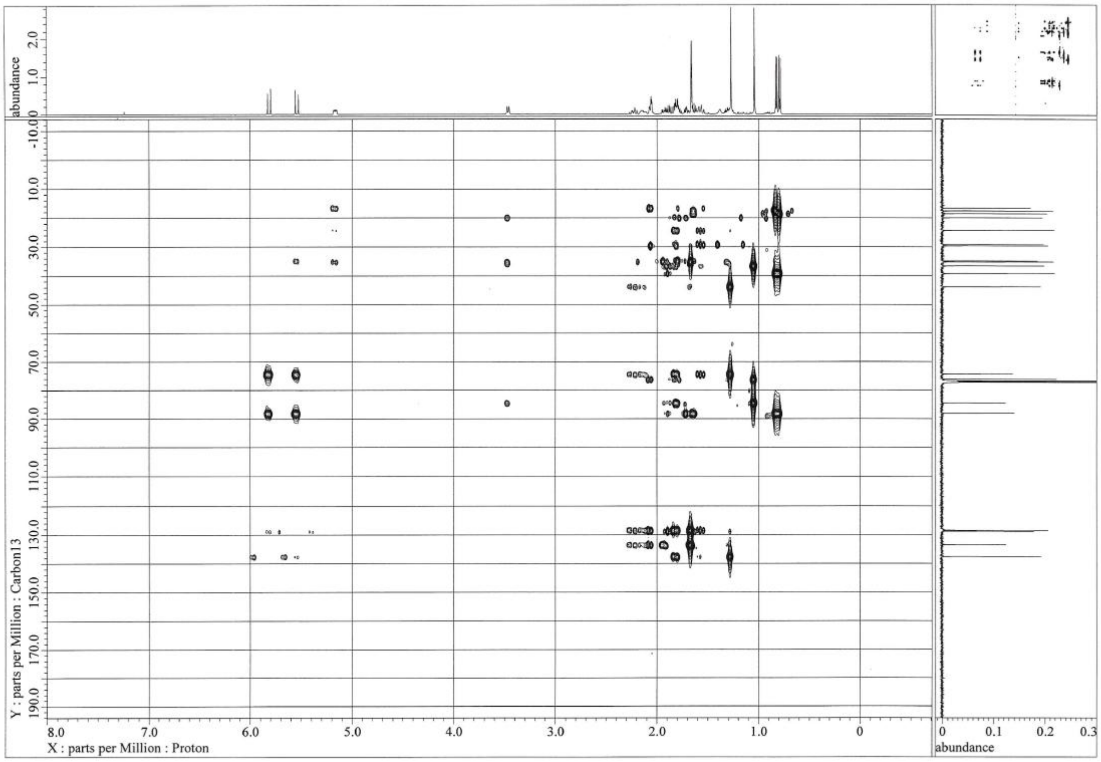

Supplementary Figure 10-6. HMBC Spectrum of compound **8**

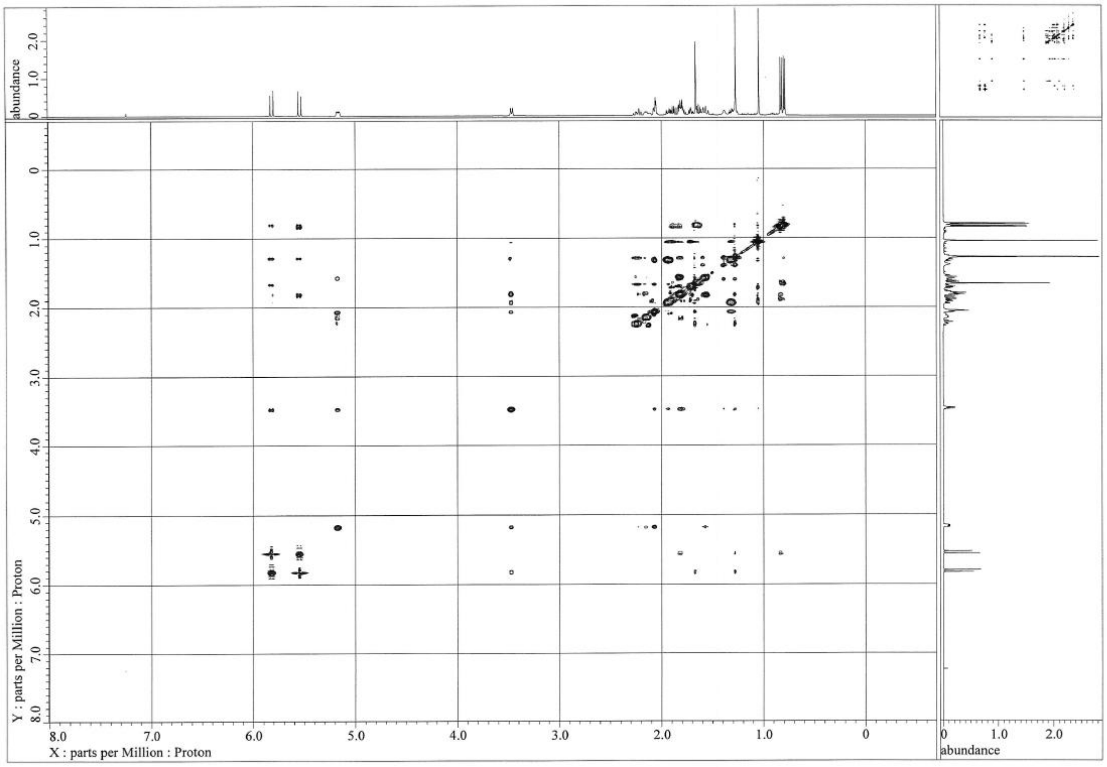

Supplementary Figure 10-7. NOESY Spectrum of compound **8**

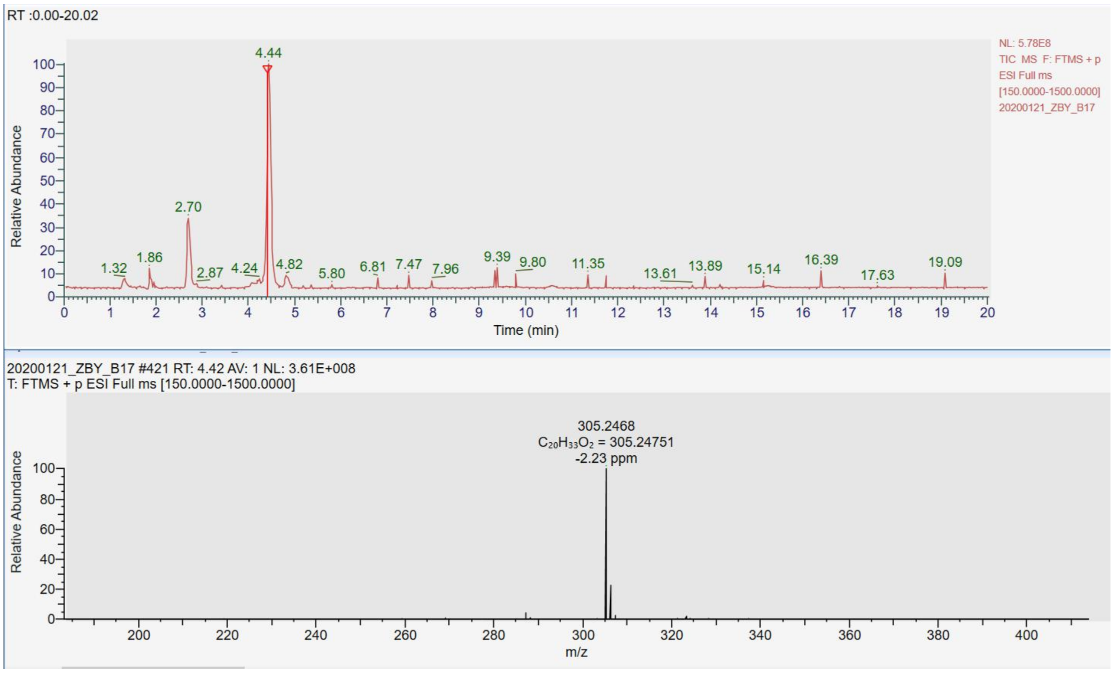

Supplementary Figure 10-8. HRESIMS data of compound **8**

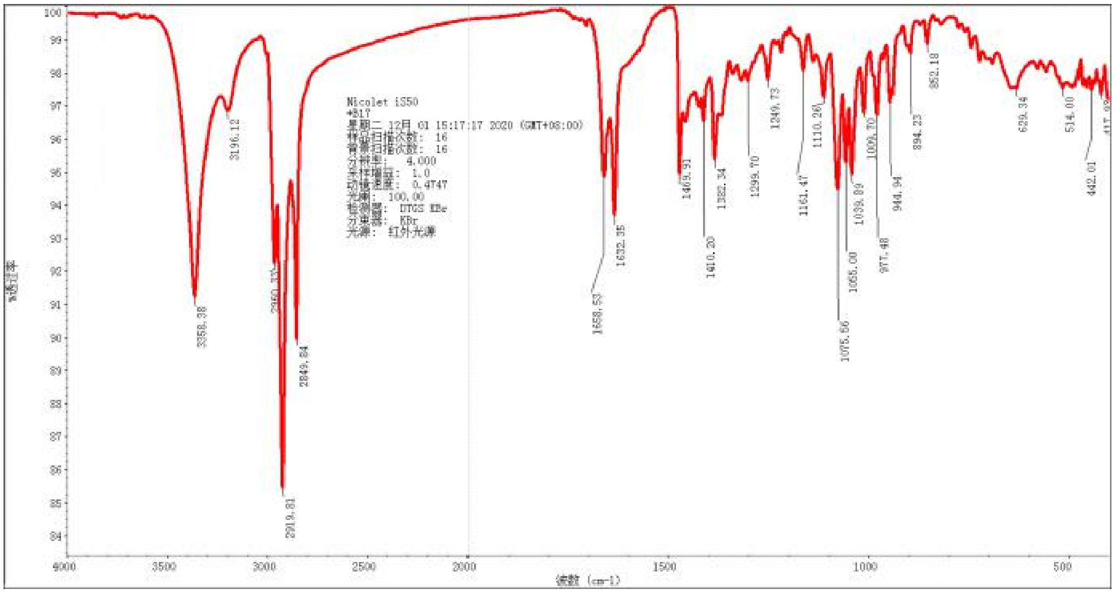

Supplementary Figure 10-9. IR Spectrum for compound **8**

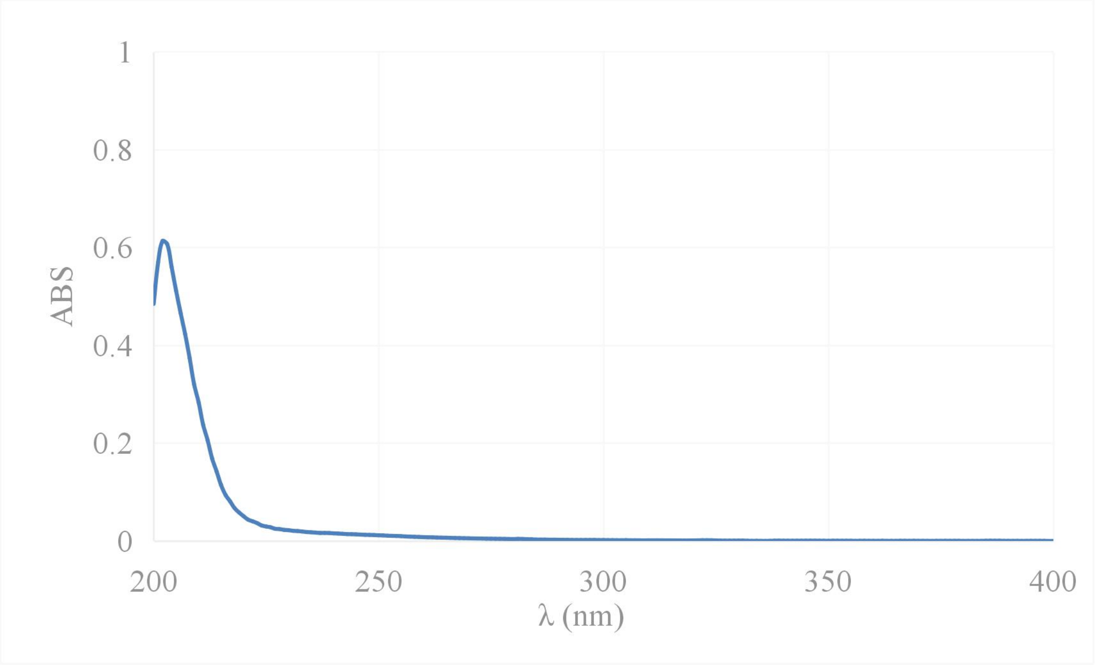

Supplementary Figure 10-10. UV Spectrum for compound **8**

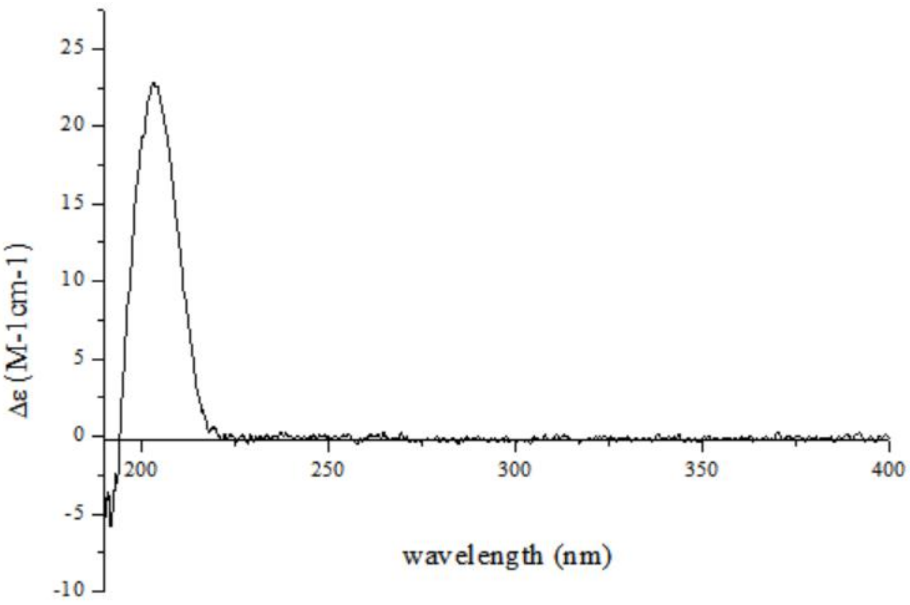

Supplementary Figure 10-11. ECD Spectrum for compound **8**

2. X-ray crystallographic data for 1

Identification code	**1**
Empirical formula	C20H36O4
Formula weight	340.49
Temperature/K	100.01(11)
Crystal system	monoclinic
Space group	I2
a/Å	23.3651(6)
b/Å	10.1181(2)
c/Å	67.6869(15)
α/°	90
β/°	91.113(2)
γ/°	90
Volume/Å3	15998.9(6)
Z	32
ρcalcg/cm3	1.131
μ/mm-1	0.608
F(000)	6016.0
Crystal size/mm3	0.13 × 0.12 × 0.11
Radiation	Cu Kα (λ = 1.54184)
2Θ range for data collection/°	4.026 to 148.16
Index ranges	-29 ≤ h ≤ 28, -8 ≤ k ≤ 12, -82 ≤ l ≤ 82
Reflections collected	58130

Independent reflections	25373 [Rint = 0.1124, Rsigma = 0.1243]
Data/restraints/parameters	25373/121/1807
Goodness-of-fit on F2	1.002
Final R indexes [I>=2σ (I)]	R1 = 0.0818, wR2 = 0.1963
Final R indexes [all data]	R1 = 0.1094, wR2 = 0.2253
Largest diff. peak/hole / e Å-3	0.54/-0.39
Flack parameter	-0.2(2)

3. ECD calculations for 4, 6-8.

Conformational searches for the molecules **4**, **6-8** were carried out using the MMFF94S force field by the Gaussian 16 (Gaussian Inc.) software (Frisch, et al., 2016). DFT calculations were used to optimize the conformers at the B3LYP/6-311+G(d) levels. Subsequently, the stable conformers for **4**, **6-8** with Boltzmann distribution ≥1% were recorded according to Gibbs free energy of the conformers, respectively. The ECD calculations for the stable conformers were performed by Gaussian 16 software. TD-DFT at the B3LYP/6-311++G(2d, p) level in MeOH solution using the IEFPCM model for ECD calculations with a total of 30 excited states for the molecules. A standard deviation of 0.35 eV was applied for ECD simulations for both configurations. Boltzmann statistics were used for final simulations of the ECD for these molecules. The optimized conformation geometries, thermodynamic parameters, and populations of all conformations were presented below.

References

Frisch, M. J.; Trucks, G. W.; Schlegel, H. B.; Scuseria, G. E.; Robb, M. A.; Cheeseman, J. R.; Scalmani, G.; Barone, V.; Mennucci, B.; Petersson, G. A.; Nakatsuji, H.; Caricato, M.; Li, X.; Hratchian, H. P.; Izmaylov, A. F.; Bloino, J.; Zheng, G.; Sonnenberg, J. L.; Hada, M.; Ehara, M.; Toyota, K.; Fukuda, R.; Hasegawa, J.; Ishida, M.; Nakajima, T.; Honda, Y.; Kitao, O.; Nakai, H.; Vreven, T.; Montgomery, J. A., Jr.; Peralta, J. E.; Ogliaro, F.; Bearpark, M.; Heyd, J. J.; Brothers, E.; Kudin, K. N.; Staroverov, V. N.; Kobayashi, R.; Normand, J.; Raghavachari, K.; Rendell, A.; Burant, J. C.; Iyengar, S. S.; Tomasi, J.; Cossi, M.; Rega, N.; Millam, J. M.; Klene, M.; Knox, J. E.; Cross, J. B.; Bakken, V.; Adamo, C.; Jaramillo, J.; Gomperts, R.; Stratmann, R. E.; Yazyev, O.; Austin, A. J.; Cammi, R.; Pomelli, C.; Ochterski, J. W.; Martin, R. L.; Morokuma, K.; Zakrzewski, V. G.; Voth, G. A.; Salvador, P.; Dannenberg, J. J.; Dapprich, S.; Daniels, A. D.; Farkas, O.; Foresman, J. B.; Ortiz, J. V.; Cioslowski, J.; Fox, D. J. Gaussian 16, Revision B.01; Gaussian, Inc.: Wallingford, CT, 2016.

Supplementary Table 1. Energy analysis for (1*S*,4*R*,7*E*,11*S*,12*R*)-**4**

Conformer	E (Hartree)	ΔE (kcal/mol)	Conformation distribution (%)
a	-1161.2890125	0.00	80.48
b	-1161.2866931	1.46	6.88
c	-1161.2865956	1.52	6.21
d	-1161.2864309	1.62	5.21

e	-1161.2838614	3.23	0.34
f	-1161.2838613	3.23	0.34
g	-1161.2830596	3.74	0.15
h	-1161.2830409	3.75	0.14
i	-1161.2824609	4.11	0.08
j	-1161.2820286	4.38	0.05
k	-1161.2820286	4.38	0.05
l	-1161.2820257	4.38	0.05
m	-1161.2806352	5.26	0.01

Supplementary Table 2. Energy analysis for (1*R*,4*S*,7*E*,11*R*,12*S*)-**4**

Conformer	E (Hartree)	ΔE (kcal/mol)	Conformation distribution (%)
a	-1161.2895320	0.00	80.65
b	-1161.2872482	1.43	7.16
c	-1161.2866512	1.81	3.80
d	-1161.2865451	1.87	3.40
e	-1161.2858842	2.29	1.69
f	-1161.2856143	2.46	1.27
g	-1161.2854222	2.58	1.03
h	-1161.2845656	3.12	0.42
i	-1161.2841374	3.39	0.26
j	-1161.2838638	3.56	0.20
k	-1161.2830368	4.08	0.08
l	-1161.2820438	4.70	0.03

Supplementary Table 3. Energy analysis for (1*S*,3*E*,7*E*,11*S*,12*R*)-**6**

Conformer	E (Hartree)	ΔE (kcal/mol)	Conformation distribution (%)
a	-1160.068888	0.00	99.61
b	-1160.063662	3.28	0.39

Supplementary Table 4. Energy analysis for (1*R*,3*E*,7*E*,11*R*,12*S*)-**6**

Conformer	E (Hartree)	ΔE (kcal/mol)	Conformation distribution (%)
a	-1160.056582	0.00	55.64

b	-1160.056309	0.17	41.67
c	-1160.053726	1.79	2.69

Supplementary Table 5. Energy analysis for (1*S*,3*E*,7*E*,11*S*,12*R*)-**7**

Conformer	E (Hartree)	ΔE (kcal/mol)	Conformation distribution (%)
a	-1007.359387	0.00	83.88
b	-1007.357825	0.98	16.01
c	-1007.352979	4.02	0.09
d	-1007.35132	5.06	0.02

Supplementary Table 6. Energy analysis for (1*R*,3*E*,7*E*,11*R*,12*S*)-**7**

Conformer	E (Hartree)	ΔE (kcal/mol)	Conformation distribution (%)
a	-1007.354815	0.00	79.96
b	-1007.353491	0.83	19.63
c	-1007.349527	3.32	0.29
d	-1007.34824	4.12	0.08
e	-1007.347597	4.53	0.04

Supplementary Table 7. Energy analysis for (1*R*,2*E*,4*R*,7*E*,11*R*,12*S*)-**8**

Conformer	E (Hartree)	ΔE (kcal/mol)	Conformation distribution (%)
a	-1008.557651	0.00	22.82
b	-1008.557651	0.00	22.80
c	-1008.557571	0.05	20.95
d	-1008.557295	0.22	15.64
e	-1008.556267	0.87	5.26
f	-1008.556258	0.87	5.21
g	-1008.555992	1.04	3.93
h	-1008.55474	1.83	1.04
i	-1008.554568	1.93	0.87
j	-1008.553697	2.48	0.34
k	-1008.553513	2.60	0.28
l	-1008.553133	2.84	0.19
m	-1008.55306	2.88	0.18

n	-1008.552733	3.09	0.12
o	-1008.552463	3.26	0.09
p	-1008.552447	3.27	0.09
q	-1008.552425	3.28	0.09
r	-1008.552152	3.45	0.07
s	-1008.550819	4.29	0.02
t	-1008.550291	4.62	0.01

Supplementary Table 8. Energy analysis for (1*S*,2*E*,4*S*,7*E*,11*S*,12*R*)-**8**

Conformer	E (Hartree)	ΔE (kcal/mol)	Conformation distribution (%)
a	-1008.5573367	0.00	45.21
b	-1008.5562689	0.67	14.57
c	-1008.5562668	0.67	14.54
d	-1008.5558197	0.95	9.05
e	-1008.5555419	1.13	6.74
f	-1008.5545684	1.74	2.40
g	-1008.5545679	1.74	2.40
h	-1008.5539767	2.11	1.28
i	-1008.5535130	2.40	0.78
j	-1008.5532610	2.56	0.60
k	-1008.5530604	2.68	0.49
l	-1008.5529754	2.74	0.44
m	-1008.5528999	2.78	0.41
n	-1008.5527276	2.89	0.34
o	-1008.5524625	3.06	0.26
p	-1008.5521519	3.25	0.19
q	-1008.5519829	3.36	0.15
r	-1008.5518296	3.46	0.13
